# Supplementary material for: Iterative computational design and crystallographic screening identifies potent inhibitors targeting the Nsp3 macrodomain of SARS-CoV-2
Source: Proc Natl Acad Sci U S A. 2023 Jan 4;120(2):e2212931120. doi: 10.1073/pnas.2212931120 (PMC9926234; doi:10.1073/pnas.2212931120)
Supplement: Supplementary file 1 — Appendix 01 (PDF) [file pnas.2212931120.sapp.pdf]

Supporting Information for

Iterative computational design and crystallographic screening identifies potent inhibitors targeting the Nsp3 Macrodomain of SARS-CoV-2

Stefan Gahbauer[1†], Galen J. Correy[2†], Marion Schuller[3], Matteo P. Ferla[4,5], Yagmur Umay Doruk[6], Moira Rachman[1], Taiasean Wu[7,8], Morgan Diolaiti[6], Siyi Wang[8], R. Jeffrey Neitz[9], Daren Fearon[10,11], Dmytro Radchenko[12,13], Yurii Moroz[13,14], John J. Irwin[1], Adam R. Renslo[6,9], Jenny C. Taylor[4,5], Jason E. Gestwicki[7,9], Frank von Delft[10,11,15,16,17], Alan Ashworth[6], Ivan Ahel[3], Brian K. Shoichet[1]* and James S. Fraser[2]*

[1]Department of Pharmaceutical Chemistry, University of California San Francisco, San Francisco, CA 94158, USA

[2]Department of Bioengineering and Therapeutic Sciences, University of California San Francisco, San Francisco, CA 94158, USA

[3]Sir William Dunn School of Pathology, University of Oxford, South Parks Road, Oxford, OX1 3RE, UK

[4]Wellcome Centre for Human Genetics, University of Oxford, Old Road Campus, Oxford OX3 7BN, UK

[5]National Institute for Health Research Oxford Biomedical Research Centre, Oxford, OX4 2PG, UK

[6]Helen Diller Family Comprehensive Cancer Center, University of California San Francisco, San Francisco, CA 94158, USA

[7]Institute for Neurodegenerative Disease, University of California San Francisco, San Francisco, CA 94158, USA

[8]Chemistry and Chemical Biology Graduate Program, University of California San Francisco, San Francisco, CA 94158, USA

[9]Department of Pharmaceutical Chemistry and Small Molecule Discovery Center, University of California, San Francisco, California 94158, USA

[10]Diamond Light Source Ltd., Harwell Science and Innovation Campus, Didcot, OX11 0DE, UK

[11]Research Complex at Harwell, Harwell Science and Innovation Campus, Didcot OX11 0FA, UK

[12]Enamine Ltd., Chervonotkatska Street 78, Kyiv 02094, Ukraine

[13]Taras Shevchenko National University of Kyiv, Volodymyrska Street 60, Kyiv, 01601, Ukraine

[14]Chemspace, Chervonotkatska Street 78, Kyiv, 02094, Ukraine

[15]Centre for Medicines Discovery, University of Oxford, South Parks Road, Headington, OX3 7DQ, UK

[16]Structural Genomics Consortium, University of Oxford, Old Road Campus, Roosevelt Drive, Headington OX3 7DQ, UK

[17]Department of Biochemistry, University of Johannesburg, Auckland Park 2006, South Africa

*Corresponding author. Email: bshoichet@gmail.com (B.K.S.); jfraser@fraserlab.com (J.S.F.)

†These authors contributed equally to this work

*Brian K. Shoichet and James S. Fraser

Email: bshoichet@gmail.com (B.K.S.); jfraser@fraserlab.com (J.S.F.)

This PDF file includes:

Supplementary Materials and Methods
Figures S1 to S10
Legends for Datasets S1 to S8

Other supporting materials for this manuscript include the following:

Datasets S1 to S8

Supplementary Materials and Methods

Computational docking

In the first docking screen, the crystal structure of SARS-CoV-2 NSP3 Mac1 bound to ADPr (PDB 6W02 (1)) was used as a template for docking. All water molecules except for HOH324, HOH344, HOH383 and HOH406 as well as chain B were removed. Next, the Mac1-ADPr complex with selected water molecules was prepared for docking following the protein prepwizard protocol of Maestro (Schrödinger v. 2019-3) (2). Accordingly, Epik was used to add protons and protonation states were optimized with PROPKA at pH 7 (3). The complex was energetically minimized using the OPLS3e force field. Thereby, the maximum heavy-atom root-mean-square deviation from the initial crystal structure was 0.3 Å. The atomic coordinates of the adenosine substructure within the co-crystallized ADPr molecule were used to generate 45 matching spheres for placement of ligand atoms by the docking program (4). For the calculation of the binding pocket electrostatic potential, the dielectric boundary between the low dielectric protein environment and high dielectric solvent was moved outwards from the protein surface by 1.9 Å using spheres generated by Sphgen. In addition, partial atomic charges of backbone amide hydrogen atoms of residues Ile23 and Phe156 were increased by 0.2 elementary charge units (e) while partial charges of the corresponding backbone carbonyl oxygen atoms were reduced by the same amount, hence, retaining the residues' net charges. Furthermore, the dielectric boundary was extended by 0.4 Å from the protein surface for the generation of ligand desolvation scoring grids (4). At the time we launched the first lead-like docking screen against Mac1, ADPr was the only known ligand of the enzyme. Consequently, we calibrated the docking parameters according to their ability to place and score adenosine, adenine and ribose within the adenosine-binding site against a background of 250 property-matched decoys generated with the DUDE-Z approach (5). In addition, an Extrema set was screened to ensure prioritization of mono-anions and neutral molecules (4).

A total of 330,324,265 molecules with molecular weights ranging from 250 to 350 amu and calculated (c)logP below 3.5 from the ZINC15 lead-like library were screened (6). In total, 316,505,043 compounds were successfully scored, each exploring on average 3,111 orientations and 405 conformations leading to the evaluation of roughly 175 trillion complexes in 65,794 core hours or roughly 66 hours on a 1000-core cluster. The predicted poses of the top-scored 500,000 molecules were filtered for internal molecular strain (total strain <6.5 TEU; maximum single torsion strain <1.8 TEU (7)) and their ability to form hydrogen bonds to residues Asp22, Ile23, Gly48, Val49, Gly130 or Phe156. Molecules with unsatisfied hydrogen bond donors or more than three unsatisfied acceptors were deprioritized (8). Finally, 90 molecules were purchased from Enamine, of which 78 (87%) were successfully synthesized.

For the second docking campaign, the crystal structure of Mac1 in complex with the first-round docking hit ZINC000078036511 (Z6511, PDB 5SOI) was used as the structural template. This ligand showed two possible conformations of the molecule's acidic functional group to either interact with the phosphate binding domain or the oxyanion site, thus offering an interesting template for exploring molecules interacting at both acid-recognizing subsites. Chain B and all water residues were removed and the Z6511-Mac1 complex (using conformation B of the ligand) was prepared according to the protein prepwizard protocol using Maestro (see above) (2). Next, fifty matching spheres for ligand placement by docking were generated based on atomic coordinates obtained from various first-round lead-like docking hits as well as previously described fragments: ZINC000078036511 (PDB 5SOI), ZINC000292637864 (PDB 5SOT), ZINC901381520 (PDB 5S6W), ZINC57162 (PDB 5RV3), ZINC26180281 (PDB 5RSF) and ZINC336438345 (PDB 5RSE) (9). These molecules were manually selected for matching sphere generation since they placed diverse functional groups at key subsites such as the adenine binding pocket, the oxyanion site or the phosphate binding domain. Enrichment calculations and parameter scans (as described in (4)) were performed by assessing the model's ability to assign high ranks to 142 previously identified fragment ligands (9) and 24 first round lead-like docking hits against a background of 2,384 experimentally determined non-binders (2,333 fragments, 51 lead-like molecules, see **Fig. S10**). Enrichment of known binders was improved by increasing the partial atomic charge of the backbone amide hydrogen atom of Ile23 by 0.4 e as well as the partial charges of the backbone

amide hydrogen atoms of Phe156 and Asp157 by 0.2 e. To maintain the residue's net charge, the partial atomic charges of each corresponding backbone carbonyl oxygen atom was decreased by the same amount. This approach of polarizing specific side chains was applied in previous docking campaigns against a wide range of different targets (see (4)). In addition, enrichment of known binders over non-binders was enhanced by modulating the dielectric boundary between the low-dielectric protein and the surrounding implicit water. After scanning a range of extensions for the low-dielectric environment away from the protein surface (1.0-1.9 Å, see (4)), moving the dielectric boundary by 1.8 Å beyond the protein surface yielded the best results.

Using the ZINC15 database, 246,246,485 neutral and monoanionic molecules from the lead-like set were docked against this Mac1 model, resulting in the scoring of 156 trillion complexes where each scored molecule was on average sampled in 3,431 orientations and 428 conformations within 63 hours on a 1000-core computer cluster. In addition, an in-house anion library containing (mostly) negatively charged molecules with molecular weight between 250 and 400 amu from the 22B Enamine REAL database was screened. In total, 39 million anions were identified by performing SMART pattern searches in RDKit (www.rdkit.org) of carboxylic acid and 33 bioisosteres. In the docking screen, 37,556,136 molecules were scored, each sampled in 4,134 orientations and 343 conformations on average resulting in the evaluation of 19.5 trillion complexes in approximately 20 hours on a 1000-core computer cluster. A final set of ca. 16 million mostly anionic molecules from the February-2020 release of Enamine REAL was docked against Mac1. Within 10,703 core hours, 15,957,174 molecules were scored by evaluating a total of 12 trillion complexes where each molecule sampled on average 5,142 orientations and 495 conformations.

The top 1 million scored compounds from each screen were investigated for intramolecular strain (total strain <7.5 TEU, maximum single torsion strain <2.5 TEU (7)) and hydrogen bonding with Asp22, Ile23, Gly48, Val49, Phe156 and Asp157. Molecules with unsatisfied hydrogen bond donors or more than three unsatisfied acceptors were not considered for experimental evaluation. The second docking campaign led to 54 molecules that we selected for synthesis at Enamine of which 46 (85%) were obtained. The small analog set designed to probe neutral alternatives of negatively moieties binding in the oxyanion subsite were docked using the parameters from the second large-scale docking campaign. Molecules were protonated using ChemAxon Jchem 2019.15 (https://chemaxon.com/) at pH 7.4, rendered into 3D with Corina (v.3.6.0026, Molecular Networks GmbH, https://mn-am.com/products/corina/) and conformational libraries were generated with Omega (v.2.5.1.4, OpenEye Scientific Software; https://www.eyesopen.com/omega).

A third docking screen was performed against the Z4305-stabilized, everted conformation of Mac1 (PDB 5SOP). Before docking to the open structure, MDMix (10) (that utilizes AMBER18 (11)) was performed to assess binding hotspots in this less explored state. For this, the protein was solvated in pre-equilibrated mixtures of 20% ethanol and water, as well as 20% methanol and water. Three replicates of 50 ns simulations (six simulations total) were performed. Settings for minimization, equilibration and the production phase were set to default (10). After the simulation, all trajectories in the three independent simulations for each solvent mixture were aligned, after which the observed density was converted to binding free energies using the inverse Boltzmann relationship. Low energy regions were visualized and inferred to be probable binding hotspots.

The crystal structure of Mac1 in complex with Z4305 was prepared for docking following the same steps as above, i.e. protonation, minimization and grid preparation. The dielectric boundary between the low dielectric protein environment and high dielectric solvent was moved outwards from the protein surface by 1.9 Å. Forty-five matching spheres were generated based on 26 atomic coordinates of Z4305 and Z5531 as well as 19 randomly placed spheres covering the oxyanion subsite and the surface near Ser128. Partial atomic charges of backbone amide hydrogen atoms of residues Ile23 were increased by 0.4 elementary charge units, while backbone amide hydrogen atoms of residues Phe156, Asp157 and Ser128 were increased by 0.2 elementary charge units. Partial charges of the corresponding backbone carbonyl oxygen atoms were reduced by the same amount. The described docking parameters were evaluated by control calculations the same way as described above for the second docking screen.

Using a new virtual library, ZINC22 (https://cartblanche22.docking.org), a collection of 60,732,663 monoanionic lead-like compounds (heavy atom count 17 to 25) were screened. Within 40 hours on a 1000-core computer cluster, roughly 57 million compounds were scored, each sampled in approx. 5,336 orientations and 359 conformations resulting in more than 54 trillion complexes. The molecules that reached a total score threshold of -35 kcal/mol (comprising 4.3 M molecules) were filtered for internal molecular strain (<6.5 TEU; maximum single torsion strain <1.8 TEU) after which 1.7 M molecules remained. Next, molecules with more than one unsatisfied hydrogen bond donor or more than three unsatisfied acceptors were removed. Four independent sets were clustered by similarity for visual inspection, namely compounds able to interact with i) Asp22, Asp157 and Ser128, ii) Asp22, Phe156 and Ser128, iii) Ile23, Phe156 and Ser128, and iv) Asp22, Ile23 and Ser128, which led to 2, 249, 1761 and 2,249 compounds, respectively, ultimately leading to 70 being purchased from Enamine, of which 56 (80%) could be synthesized.

Crystallization and ligand soaking

Crystals of SARS-CoV-2 NSP3 Mac1 were grown using an expression construct that crystallized in the $P4_3$ space group, as described previously (9) (**Dataset S2**). This construct crystallizes with two molecules in the asymmetric unit: the active site of protomer A is accessible to ligands while the active site of protomer B is obstructed by a crystal lattice interaction (9). The $P4_3$ crystal system was chosen because the crystals grow readily, diffract to atomic resolution and tolerate soaks in 10% DMSO for at least 6 hours (9). Briefly, crystals were grown by microseeding in 96-well sitting drop plates (SWISSCI, 3W96T-UVP), using 30 µl of 28% PEG 3000 and 100 mM CHES pH 9.5 in the reservoir and crystallization drops containing 100 nl seeds, 100 nl reservoir and 200 nl protein (40 mg/ml in 150 mM NaCl, 20 mM Tris pH 8.5, 5% glycerol and 2 mM DTT). Crystals grew to maximum size in ~24 hours at 19°C. Compounds were prepared in DMSO to 100 mM, or to the maximum concentration allowed by solubility (see **Dataset S2** for compound concentrations). Compounds in DMSO were added to crystallization drops using acoustic dispensing with an Echo 650 liquid handler (Labcyte) (12). Soaks were performed with either 40 or 80 nl of compound per crystallization drop, giving a nominal concentration of 10 or 20 mM (see **Dataset S2**). After incubating for 2-4.5 hours at room temperature, crystals were vitrified in liquid nitrogen using a Nanuq cryocooling device (Mitegen). No additional cryoprotectant was added prior to vitrification. Although there was no observed decrease in diffraction quality with increased soak time (**Fig. S9**), certain compounds (namely Z8601 and LRH-0003) induced substantial disintegration of crystals after two hours, possibly linked to disruption of the crystal lattice by binding of compounds to the protomer B active site (9). Despite the crystal disintegration, reflections were recorded to <1 Å for crystals soaked with both compounds (**Dataset S2**).

X-ray diffraction data collection and data reduction,

Diffraction datasets were collected at beamline 8.3.1 at the Advanced Light Source, beamlines 12-1 and 12-2 at the Stanford Synchrotron Radiation Lightsource, or beamline 17-ID-2 at the National Synchrotron Light Source II. The data collection parameters used at each beamline are listed in **Dataset S2**. X-ray diffraction images were indexed, integrated and scaled with XDS (13), using a reference $P4_3$ dataset to ensure consistent indexing. The high resolution limit for each dataset was chosen based on a $CC_{1/2}$ value of ~0.3 in the highest resolution shell (14). The diffraction resolution of crystals frequently exceeded the maximum resolution achievable with the experimental set-up; for these datasets, the high resolution limit was set to achieve ~95% completeness in the highest resolution shell. Data were merged with Aimless (15), and free R flags were copied from a reference $P4_3$ dataset. Structure factors intensities for all datasets have been uploaded to Zenodo in MTZ format (DOI: 10.5281/zenodo.6856943). For some compounds, datasets were collected from multiple crystals. Data collection and reduction statistics for all datasets summarized are in **Dataset S2**.

Ligand identification, modeling and refinement

All datasets were initially refined with the Dimple pipeline (16) run through CCP4 (17) using a starting model refined from a crystal soaked only in DMSO (dataset UCSF-P0110 in **Dataset S2**). Ligands were identified using PanDDA version 0.2.14 (18), with a ground-state map calculated

using 34 datasets collected from crystals soaked only in DMSO. PanDDA was run an additional two times with ground-state maps calculated using 35 or 62 datasets from the ligand-soaked crystals where no ligands were detected. This procedure led to the identification of an additional 19 binding events, four of which were not identified in the first PanDDA run. Datasets used for ground-state map calculation for each of the PanDDA runs are annotated in **Dataset S2**. For ligands with multiple crystals/datasets, only the highest occupancy event was modeled. Ligands were modeled into PanDDA event maps using COOT version 0.8.9.2 (19) with ligand restraints generated using *phenix.elbow* (20) or ACEDRG (21) from a SMILES strings, or from coordinates generated using LigPrep version 2022-1 (22). Based on the background density correction (BDC) values, ligand occupancies ranged from ~10-90% (**Dataset S2**). Many of the ligands had multiple conformations and/or isomers present. The isomers modeled, and the estimated ratios based on PanDDA event maps, are listed in **Dataset S2**. Datasets were collected from soaks performed with two batches of Z8539_0002; one of the datasets was modeled with the (*R*,*R*) and (*S*,*S*) isomers (PDB 5SQD), while the other was only modeled with the (*R*,*S*) isomer (PDB 5SSN). Two compounds, LRH-0022 (PDB 5SRH) and LRH-0031 (PDB 5SRI), were only modeled with their pyrimido-indole core.

For all ligands, we modeled changes in protein structure and water in the ligand binding sites into PanDDA event maps. Alternative conformations were included for residues where the heavy-atom RMSD value of the ligand-bound model to the ground-state model was greater than 0.15 Å. This cut-off was chosen with reference to the RMSD values for the 34 ground-state structures, where 99.7% of residues had RMSD values <0.15 Å (**Fig. S9**). In these multi-conformer models, the ground-state model was assigned the alternative occupancy identifier (altloc) A and the ligand-bound state was assigned altloc B (and C/D when overlapping conformations/isomers were present). Water molecules modeled into PanDDA event maps were assigned altloc B, and ground-state water molecules were included within 2.5 Å of ligand-bound state ligands or water (assigned altloc A).

Refinement of the ligand-bound multi-conformer models was performed with *phenix.refine* using five refinement macrocycles (23). Coordinates and atomic displacement parameters (ADPs) were refined for all protein heavy atoms, and hydrogens were refined using a riding model. Based on previous observations (18), the occupancy of the ligand-bound and ground-states were set to 2*(1-BDC) and 1-2*(1-BDC) respectively, and occupancy refinement was switched off. Water molecules were automatically added to peaks in the mF_O-DF_C difference density map >3.5 σ using *phenix.refine*. To prevent the multi-conformer water molecules being removed by the automatic solvent picking, the ligand- and ground-state waters were renamed from HOH to WWW. After one round of refinement, maps and coordinates were inspected, and additional water molecules were placed manually using COOT into peaks in the mF_O-DF_C difference map. Based on positive/negative peaks in the mF_O-DF_C difference maps after refinement, the occupancies for some ligands were adjusted (initial and adjusted occupancies are listed in **Dataset S2**). Next, a second round of refinement was performed with ADPs refined anisotropically for non-hydrogen atoms, with automatic water picking, and the refinement of water coordinates, switched off. Data refinement statistics are summarized in **Dataset S2**. Coordinates, structure factor intensities and PanDDA event maps for all datasets have been deposited in the Protein Data Bank under the group deposition IDs G_1002236, G_1002238 and G_1002239. Additionally, the PanDDA input and output files have been uploaded to Zenodo (DOI: 10.5281/zenodo.6856943).

Homogeneous Time Resolved Fluorescence assay

Compounds were dispensed into ProxiPlate-384 Plus (PerkinElmer) assay plates using an Echo 525 liquid handler (Labcyte). Binding assays were conducted in a final volume of 16 µl with 12.5 nM NSP3 Mac1 protein, 400 nM peptide ARTK(Bio)QTARK(Aoa-RADP)S (Cambridge Peptides), 1:20000 Anti-His6-Eu3+ cryptate (HTRF donor, PerkinElmer) and 1:125 Streptavidin-XL665 (HTRF acceptor, PerkinElmer) in assay buffer (25 mM 4-(2-hydroxyethyl)-1-piperazineethanesulfonic acid (HEPES) pH 7.0, 20 mM NaCl, 0.05% bovine serum albumin and 0.05% Tween-20). TARG1 and MacroD2 binding were measured at 100 nM and 12.5 nM, respectively. Assay reagents were dispensed manually into plates using a multichannel pipette while macrodomain protein and

peptide were first dispensed and incubated for 30 min at room temperature. This was followed by addition of the HTRF reagents and incubation at room temperature for 1 h. Fluorescence was measured using a PHERAstar microplate reader (BMG) using the HTRF module with dual emission protocol (A = excitation of 320 nm, emission of 665 nm, and B = excitation of 320 nm, emission of 620 nm) or a Synergy H1 (Biotek) using the HTRF filter set (A = excitation 330/80 nm, emission of 620/10 nm, and B = excitation of 330/80 nm and emission of 665/8 nm). Raw data were processed to give an HTRF ratio (channel A/B × 10,000), which was used to generate IC_{50} curves. The IC_{50} values were determined by nonlinear regression using GraphPad Prism v.8 (GraphPad Software, CA, USA).

MDR1-MDCK II cell permeability

Canine MDR1 knockout, human MDR1 knockin MDCKII cell (MDR1-MDCKII) (Sigma-Aldrich, MTOX1303) suspension (400 µl) was added to each well of high throughput screening multiwell insert system plates. Test compounds were prepared as 20 mM DMSO stocks. The test compound (300 µl) was dissolved in transport buffer (9.5 g/l Hanks' balanced salt solution and 0.35 g/l $NaHCO_3$ with 0.81 mM $MgSO_4$, 1.26 mM $CaCl_2$, 25 mM HEPES, pH adjusted to 7.4) and added into filter wells whereas 1000 µl of transport buffer was added to transport analysis plate wells in order to determine apical (A) to basolateral (B) transport. Basolateral to apical transport was measured by adding 1000 µl of the test compound solution into the transport analysis plate wells whereas 300 µl of buffer was used to fill the filter plate wells. Final concentrations of test compounds were 10 µM. Plates were incubated for 90 min at 37°C under continuous shaking (100 rpm), 75 µl aliquots were taken from the donor and receiver compartments for LC-MS/MS analysis. Samples were mixed with acetonitrile followed by protein sedimentation by centrifugation at 1000 rpm for 10 min. HPLC coupled with tandem mass spectroscopy was performed using the Shimadzu Prominence HPLC system coupled with the API 5000 (PE Sciex) spectrometer. Both the positive and negative ion modes of the TurboIonSpray ion source were used. The apparent permeability (Papp) was computed using the equation 1, where V_A is the volume of transport buffer in acceptor wel, *Area* is the surface area of the insert, *Time* is the assay time, $[drug]_{acc}$ is the peak area of test compound in acceptor well, and $[drug]_{initial,d}$ is the initial amount of the test compound in a donor well.

$$Papp = \frac{V_A}{Area \times Time} \times \frac{[compound]_{acc}}{[compound]_{initial,d}} \qquad (1)$$

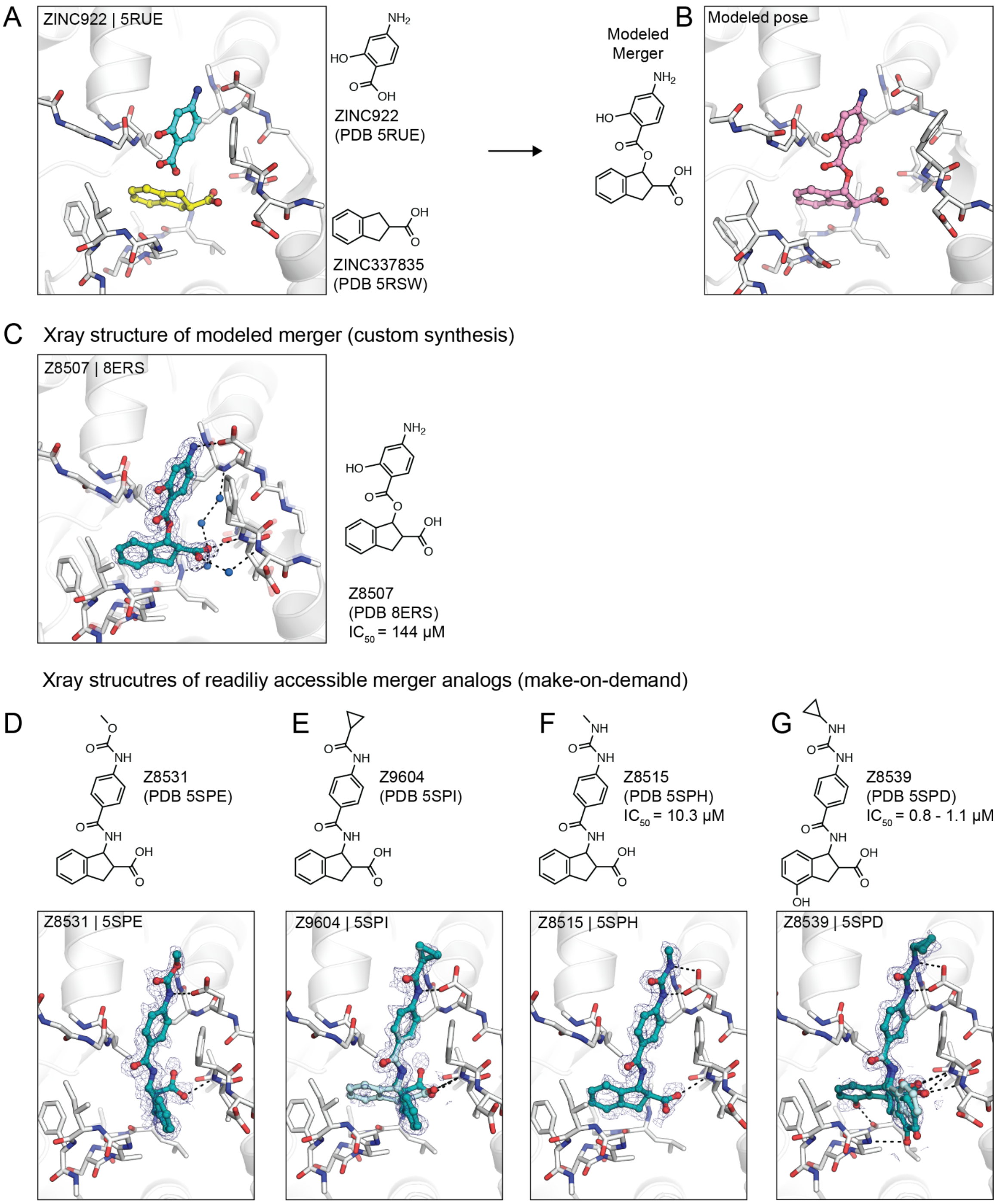

Figure S1. Overview of fragment linking approach that led to Z8539. A) Alignment of X-ray crystal structures of the fragments ZINC922 and ZINC337835. **B)** Linked fragments designed by *Fragmenstein* modeled into the Mac1 active site. **C)** X-ray crystal structure of linked Fragmenstein compound Z8507. **D-G)** 2D and X-ray crystal structures of purchased and tested analogs of the *Fragmenstein*-derived merger. PanDDA event maps are shown for ligands (blue mesh contoured at 2 σ).

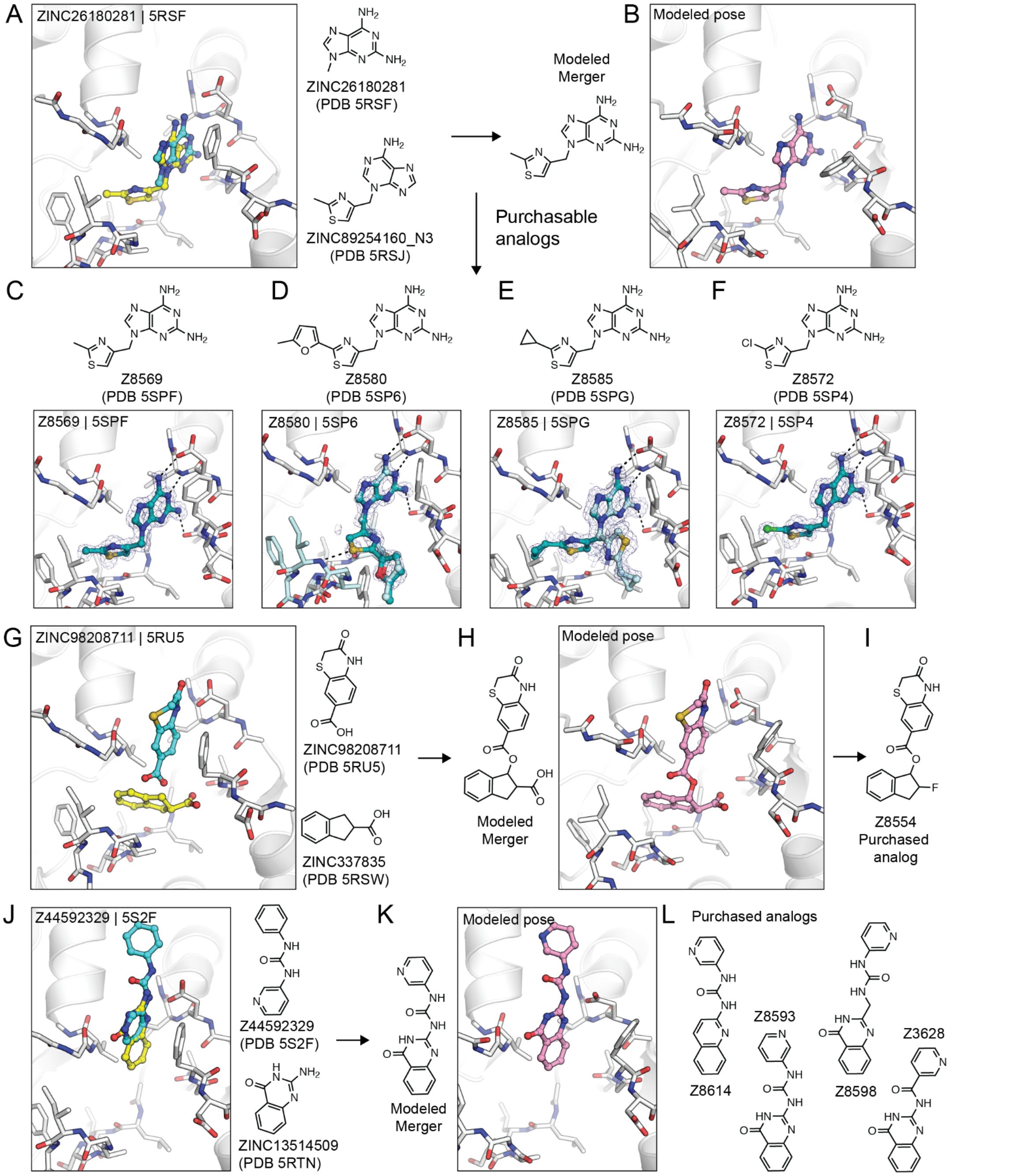

Figure S2. Fragment linking and merging with *Fragmenstein*. A) Overlay of the ZINC26180281 and ZINC89254160_N3 fragments identified in the initial screen (24). **B)** Modeled ZINC26180281-ZINC89254160_N3 merger. **C-F)** 2D and X-ray crystal structures of purchased and tested analogs of the ZINC26180281-ZINC89254160_N3 merger. PanDDA event maps are shown around the ligands (blue mesh contoured at 2 σ). **G)** Overlay of the ZINC98208711 and ZINC337835 fragments. **H)** Modeled ZINC98208711-ZINC337835 merger. **I)** Purchased and tested analog of the ZINC98208711-ZINC337835 merger. **J)** Overlay of the Z44592329 and ZINC13514509 fragments. K) Modeled Z44592329-ZINC13514509 merger. L) Purchased and tested analogs of the Z44592329-ZINC13514509 merger.

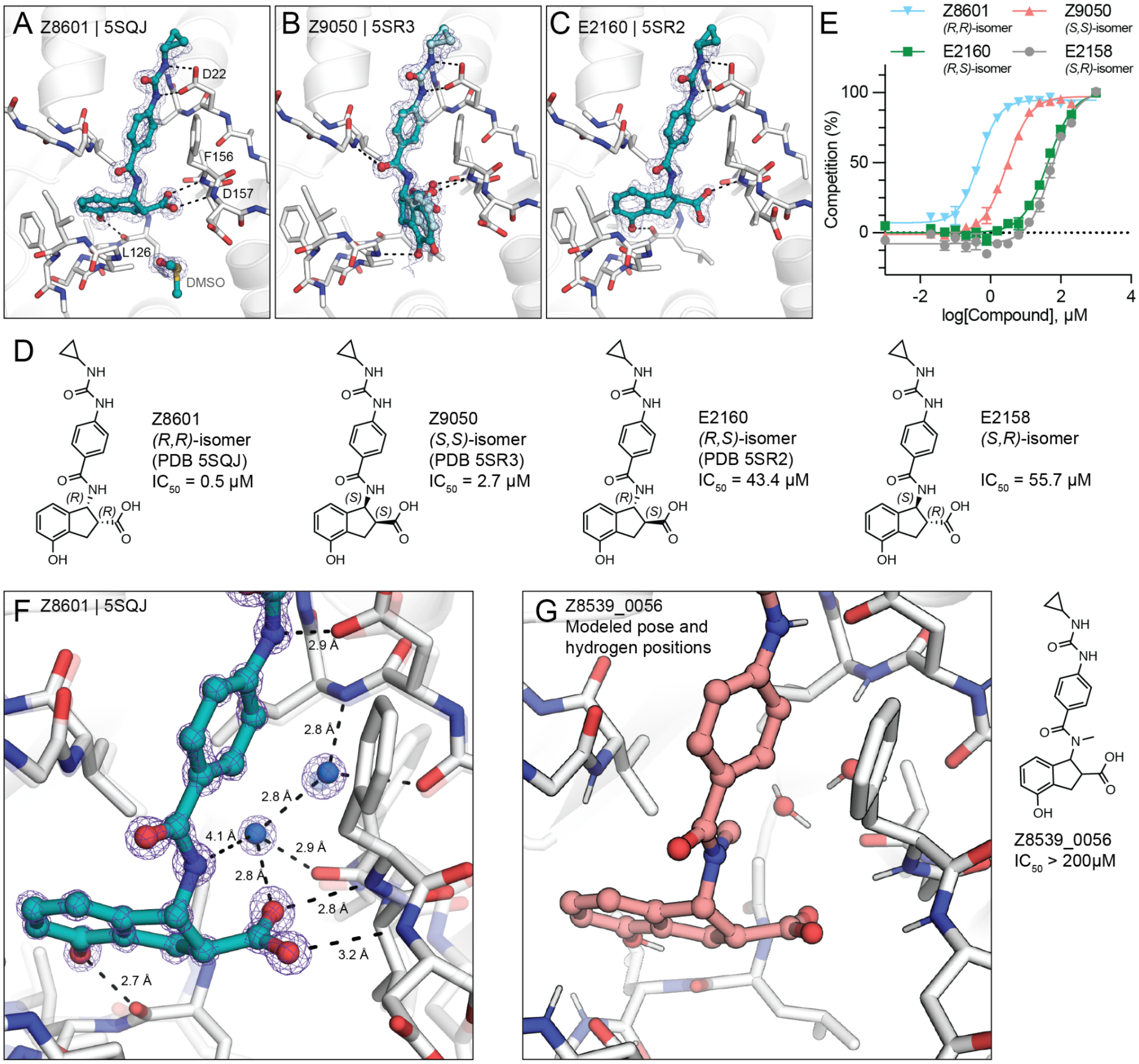

Figure S3. Chiral separation and testing of the Z8539 stereoisomers. A-C) Crystal structures of three of the four possible Z8539 stereoisomers. PanDDA event maps are shown around the ligands (blue mesh contoured at 2 σ). **D)** 2D structures and HTRF-derived IC_{50} values of Z8539 stereoisomers. **E)** HTRF-based binding curves of Z8539 stereoisomers. Water-mediated interaction between Mac1 and Z8601. **F)** X-ray crystal structure of Mac1 bound to the (*R*,*R*) isomer of Z8539. The $2mF_O\text{-}DF_C$ electron density is shown around the ligand and nearby solvent after refinement (purple mesh contoured at 3 σ). The ligand-bound state (teal/white sticks) was refined at 78% occupancy and the apo state (transparent sticks and spheres) was refined at 22% occupancy. **G)** Same view as **(F)**, but showing the modeled binding pose of the inactive, methylated analog Z8539_0056 with nearby solvent.

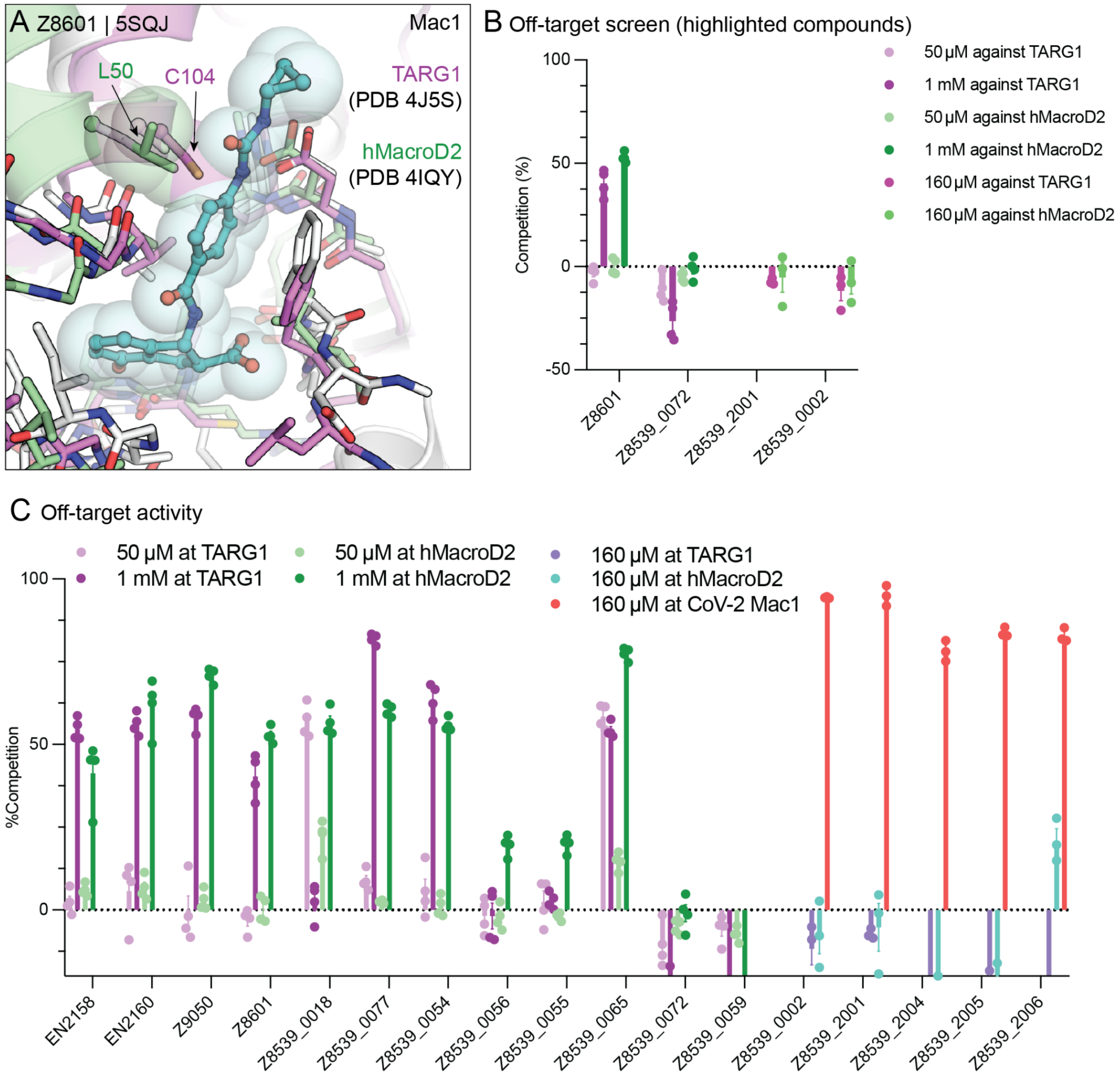

Figure S4. Assessment of ligand selectivity against the human macrodomains MacroD2 and TARG1. A) Alignment of the crystal structure of Mac1 bound to Z8601 (PDB 5SQJ) with the structures of TARG1 (25) and MacroD2 (26) bound to ADPr **B,C)** Off-target activities of Z8539 analogs against human macrodomains TARG1 and MacroD2.

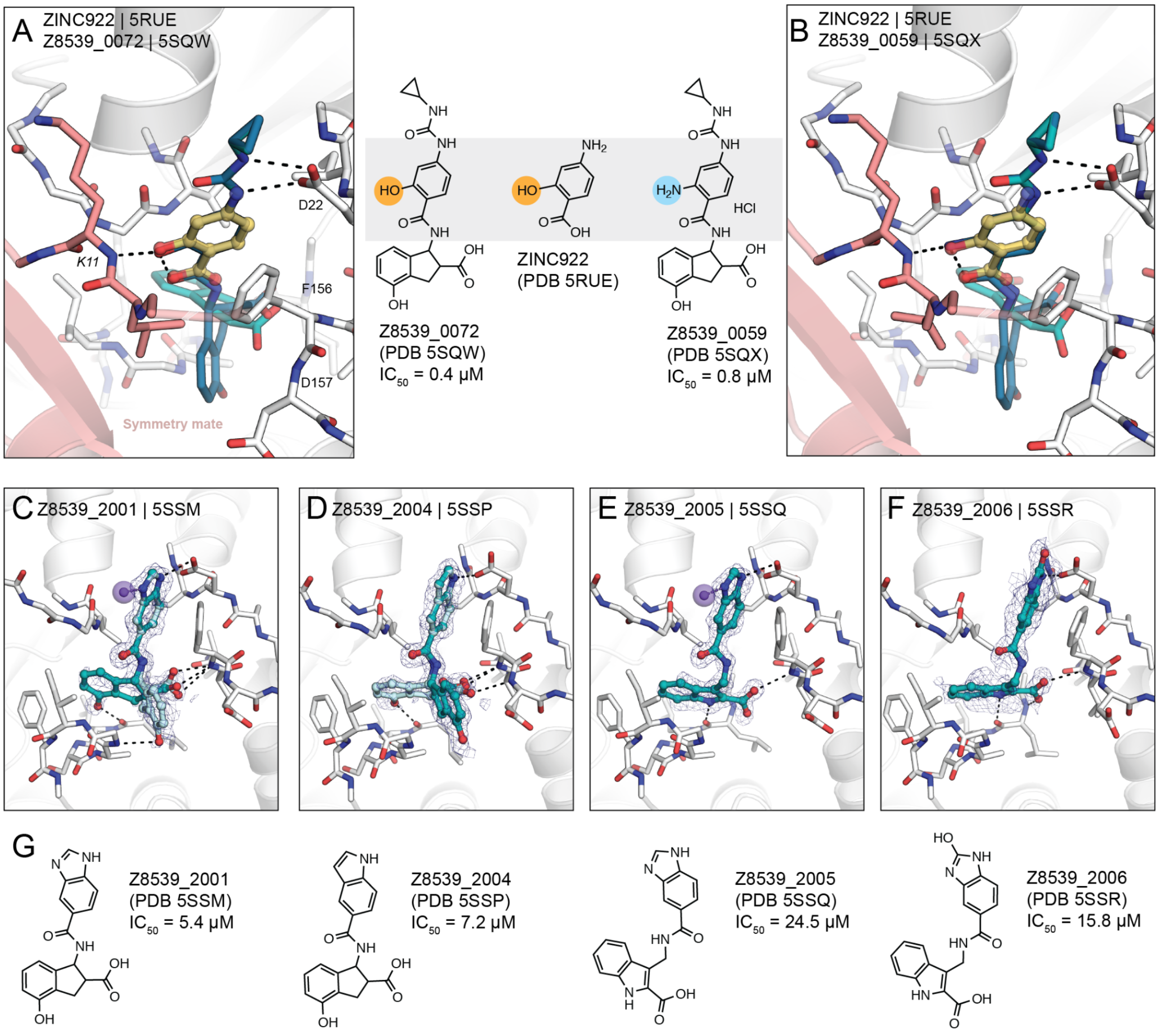

Figure S5. Crystal packing interactions of Z8539 derivatives. A-B) Interactions of Z8539_0072 or Z8539_0059 within the crystal lattice, respectively. As observed for the initial fragment hit ZINC922, the added hydroxyl (or amine) group forms a hydrogen to the Lys11 of the symmetry mate. **C-F)** Crystal structure of Z8539_2001, Z8539_2004, Z8539_2005 and Z8539_2006, respectively. PanDDA event maps are shown for the ligands (contoured at 2 σ). **G)** Corresponding 2D structures and HTRF-derived IC_{50} values.

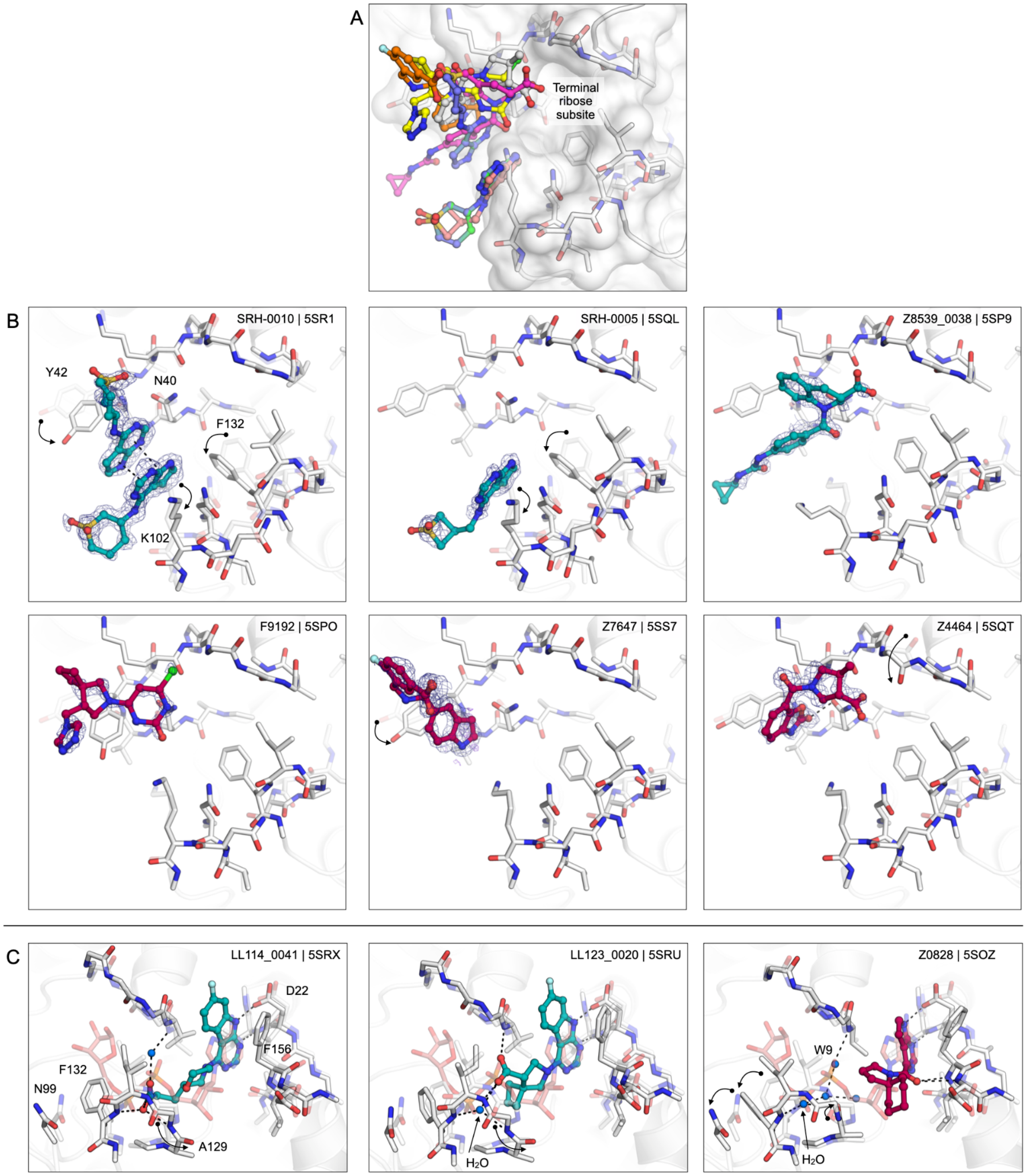

Figure S6. Ligands binding in the catalytic site of Mac1. A) Overlay of the six ligands identified binding in the catalytic site of Mac1. **B)** Structures of the six ligands with conformational changes relative to the apo structure (transparent white sticks) annotated with black arrows. PanDDA event maps are shown around the ligands (blue mesh contoured at 2 σ). **C)** LL114_0041 stabilizes an alternative, flipped conformation of Ala129 relative to the apo state (transparent white sticks). The flipped conformation matches the ADPr-bound structure (ADPr is shown with transparent red sticks). The flipped conformation is also stabilized in the structure with LL123_0020 bound, but a water molecule occupies the key phosphate binding site. A similar rearrangement of the water network is seen for Z0828.

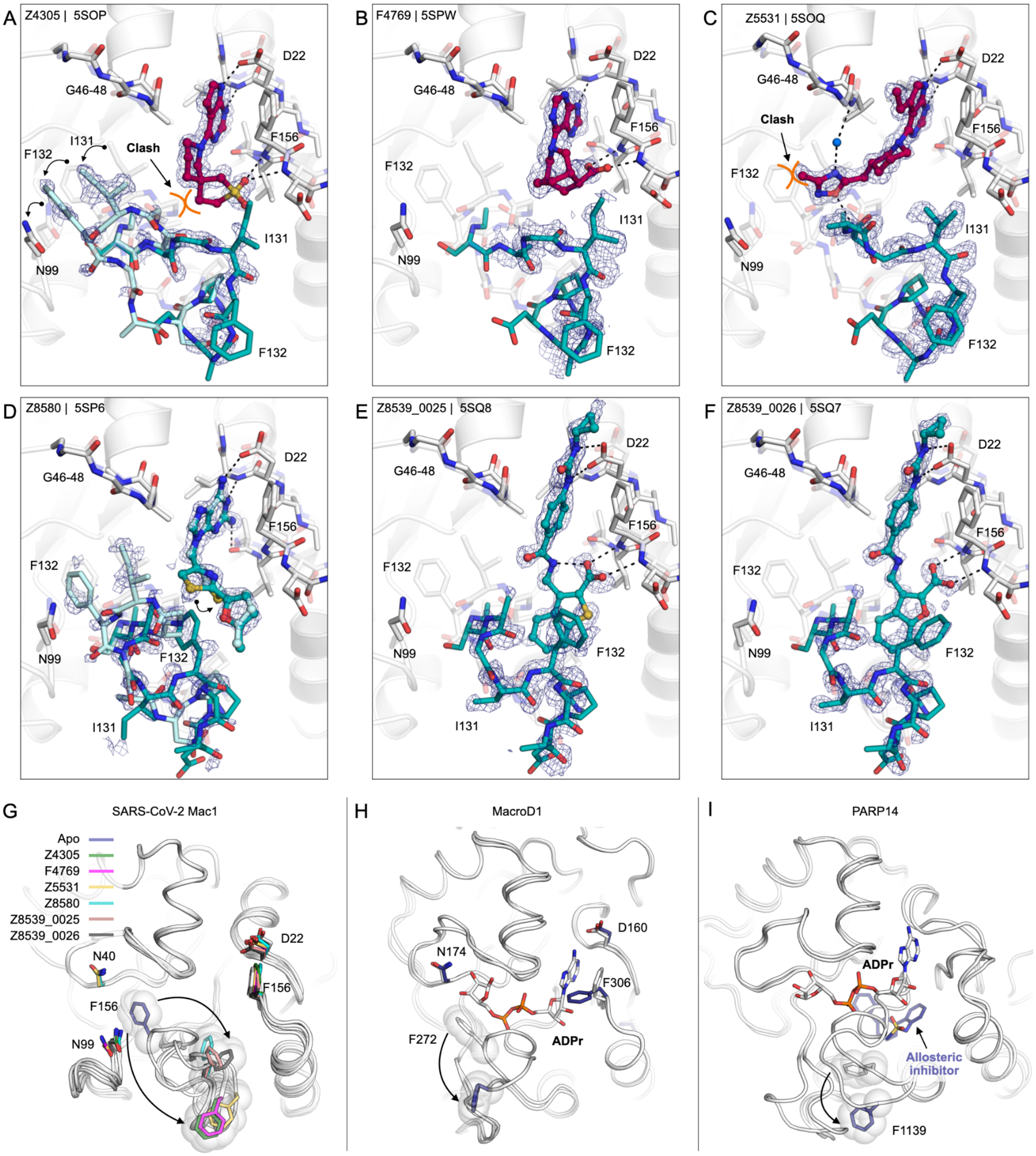

Figure S7. Everted structures of Mac1. A-F) X-ray crystal structures of the six ligands that stabilize the everted state of Mac1. PanDDA event maps are shown around the ligands and residues 129-135 (blue mesh contoured at 2 σ). Two conformations of the 129-135 loop were modeled in the structures of Z4305 and Z8580 (shown with teal/cyan sticks). **G)** Overlay of the six everted state structures with the apo state. Sticks are colored according to the ligand bound, and the ligands are omitted for clarity. **H)** Structure of MacroD1 showing Phe272 in an everted state (PDB 2X47 (27), blue sticks). The everted structure is overlaid with an ADPr-bound structure (PDB 6LH4 (28), white sticks). **I)** Structure of PARP14 macrodomain 2 bound to an allosteric inhibitor (PDB 5O2D (29), blue sticks). The structure is overlaid with an ADPr-bound structure (PDB 3Q71 (30), white sticks).

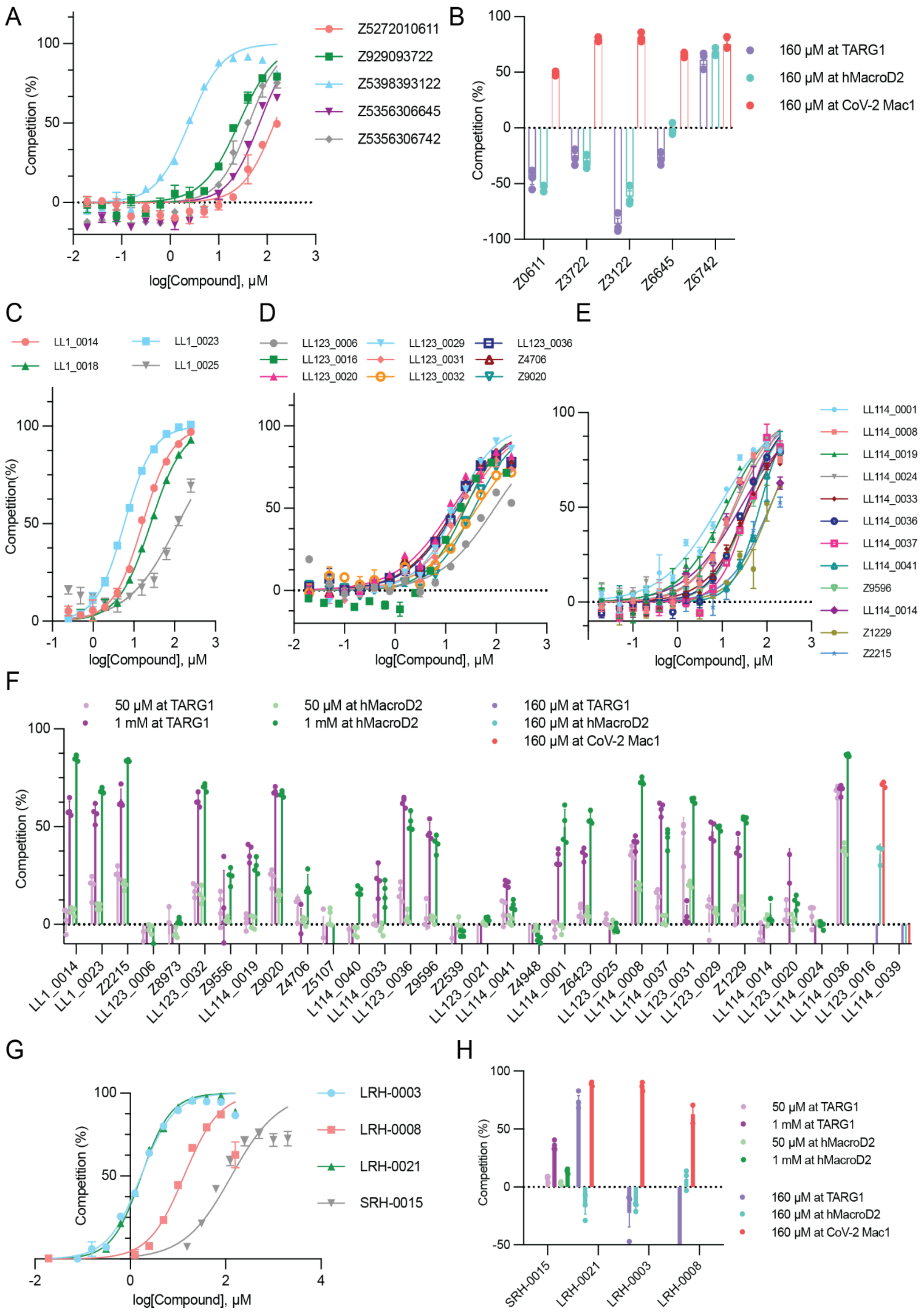

Figure S8. Assessing activity and selectivity of Mac1 ligands. A) HTRF-based peptide displacement assay of hits found against the everted structure of Mac1. Data are presented as the mean ± SEM of three technical repeats. **B)** Off-target activities of open-state hits. **C-E)** HTRF-based binding curves of compounds from the LL1 series. **F)** Off-target activities of LL1 compounds. **G)** HTRF-based binding curves of neutral probe set compounds. **H)** Off-target activities of neutral probe set hits.

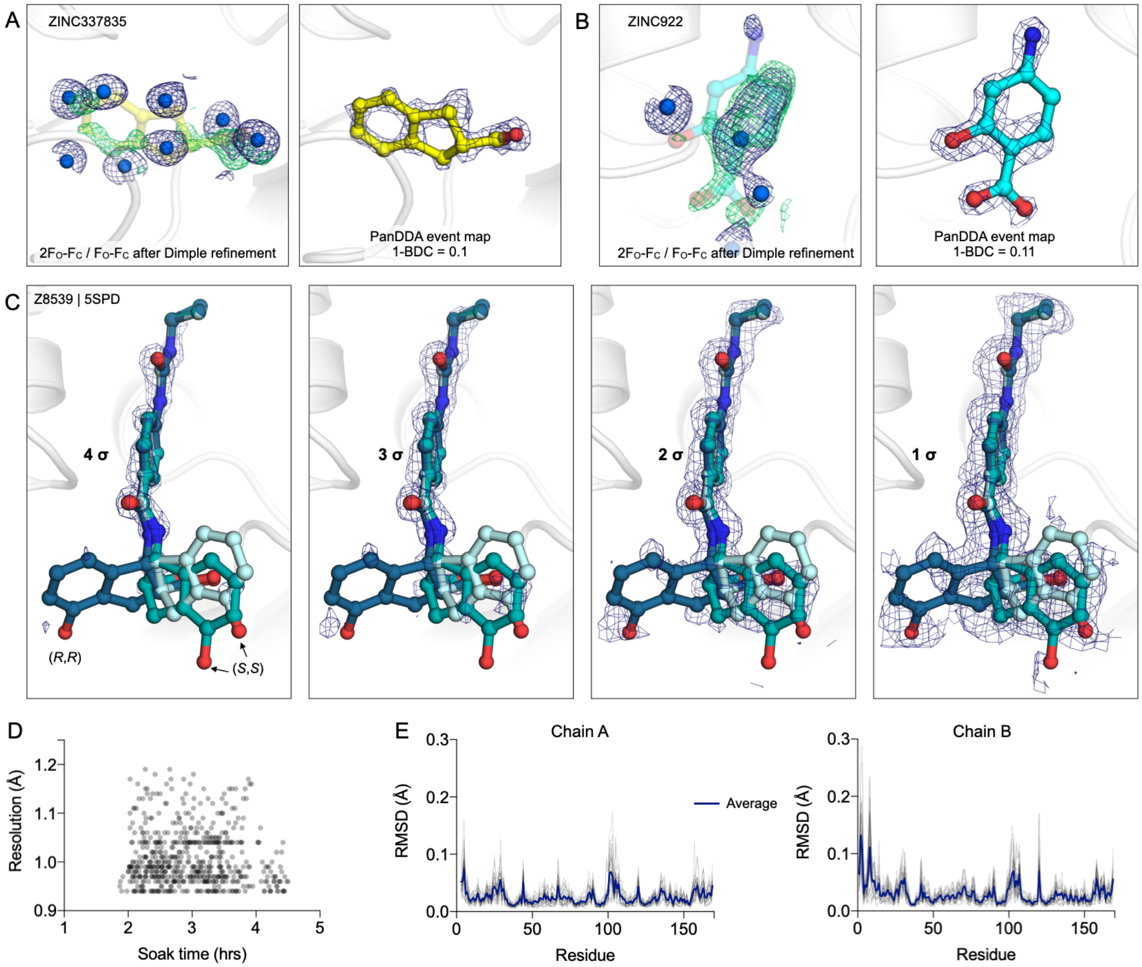

Figure S9. PanDDA reveals clear density for low occupancy states. A) Electron density maps after Dimple refinement (left) and PanDDA (right) from a crystal soaked with ZINC337835 (9). There is weak positive difference density consistent with the fragment in the F_O-F_C map (green mesh contoured at 2 σ around the fragment), however this is largely obscured by modeled solvent in the 2F_O-F_C map (blue mesh contoured at 1 σ around the fragment). In contrast, the fragment is unambiguously present in the PanDDA event map (blue mesh contoured at 2 σ around the fragment). **B)** Same as **(A)** but showing Dimple and PanDDA maps for ZINC922. **C)** PanDDA event maps helped to resolve conformational/chemical heterogeneity for the stereoisomeric mixture of Z8539 soaked into Mac1 crystals. Each panel shows the three modeled conformations of Z8539 [(*R*,*R*) and two conformations of (*S*,*S*)] with the PanDDA event density contoured from 4-1 σ. The 1-BDC value for the ligand binding event was 0.2, which is consistent with an occupancy of 40% (18). Given the relative intensity of the PanDDA map, this suggests that the (*R*,*R*) isomer is likely present at <10% occupancy. **D)** Plot showing resolution of all 591 datasets collected as a function of soak time. **E)** Plot showing per-residue heavy-atom RMSD of each of the 34 models obtained from crystals soaked in DMSO after alignment to the refinement starting model.

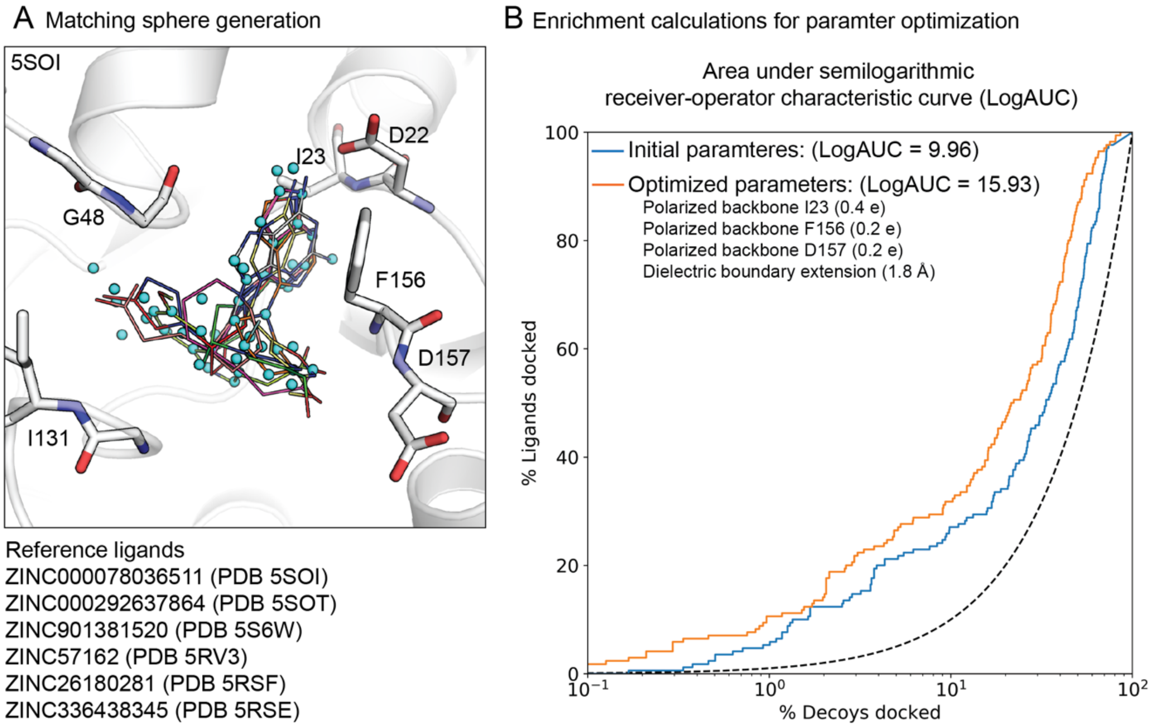

Figure S10. Modeling and parameter optimization for the second docking screen. A) Fifty matching spheres (cyan) for ligand placement were generated based on atomic coordinates of six previously identified molecules occupying different sub-pockets within the active site. **B)** Enrichment calculations guiding parameter optimization. The area under the semilogarithmic receiver-operator characteristic curve allows to assess the models' performances to enrich known actives against a background of decoys, especially to focus on early enrichment.

Dataset S1 (separate file). Spreadsheet showing all compounds tested in this work.

Dataset S2 (separate file). Spreadsheet documenting the X-ray crystallography results reported in this work.

Dataset S3 (separate file). Images showing crystal structures of Mac1 in complex with compounds from the Z8539 series.

Dataset S4 (separate file). Images showing crystal structures of Mac1 in complex with docking hits.

Dataset S5 (separate file). Images showing crystal structures of Mac1 in complex with analogs of docking hits (LL1 series).

Dataset S6 (separate file). Images showing design of neutral probe set.

Dataset S7 (separate file). Images showing the raw DSF data.

Dataset S8 (separate file). Spreadsheet showing the mean and standard deviation for all calculated Tmas from DSF.

SI References

1. K. Michalska, *et al.*, Crystal structures of SARS-CoV-2 ADP-ribose phosphatase: from the apo form to ligand complexes. *IUCrJ* **7**, 814–824 (2020).

2. G. Madhavi Sastry, M. Adzhigirey, T. Day, R. Annabhimoju, W. Sherman, Protein and ligand preparation: parameters, protocols, and influence on virtual screening enrichments. *J. Comput. Aided Mol. Des.* **27**, 221–234 (2013).

3. M. H. M. Olsson, C. R. Søndergaard, M. Rostkowski, J. H. Jensen, PROPKA3: Consistent Treatment of Internal and Surface Residues in Empirical pKa Predictions. *J. Chem. Theory Comput.* **7**, 525–537 (2011).

4. B. J. Bender, *et al.*, A practical guide to large-scale docking. *Nat. Protoc.* **16**, 4799–4832 (2021).

5. R. M. Stein, *et al.*, Property-Unmatched Decoys in Docking Benchmarks. *J. Chem. Inf. Model.* **61**, 699–714 (2021).

6. T. Sterling, J. J. Irwin, ZINC 15 – Ligand Discovery for Everyone. *Journal of Chemical Information and Modeling* **55**, 2324–2337 (2015).

7. S. Gu, M. S. Smith, Y. Yang, J. J. Irwin, B. K. Shoichet, Ligand Strain Energy in Large Library Docking. *J. Chem. Inf. Model.* **61**, 4331–4341 (2021).

8. A. V. Fassio, *et al.*, Prioritizing virtual screening with interpretable interaction fingerprints. *bioRxiv*, 2022.05.25.493419 (2022).

9. M. Schuller, *et al.*, Fragment binding to the Nsp3 macrodomain of SARS-CoV-2 identified through crystallographic screening and computational docking. *Sci Adv* **7** (2021).

10. D. Alvarez-Garcia, X. Barril, Molecular simulations with solvent competition quantify water displaceability and provide accurate interaction maps of protein binding sites. *J. Med. Chem.* **57**, 8530–8539 (2014).

11. J. Wang, R. M. Wolf, J. W. Caldwell, P. A. Kollman, D. A. Case, Development and testing of a general amber force field. *J. Comput. Chem.* **25**, 1157–1174 (2004).

12. P. M. Collins, *et al.*, Gentle, fast and effective crystal soaking by acoustic dispensing. *Acta Crystallogr D Struct Biol* **73**, 246–255 (2017).

13. W. Kabsch, XDS. *Acta Crystallogr. D Biol. Crystallogr.* **66**, 125–132 (2010).

14. P. A. Karplus, K. Diederichs, Linking crystallographic model and data quality. *Science* **336**, 1030–1033 (2012).

15. P. R. Evans, G. N. Murshudov, How good are my data and what is the resolution? *Acta Crystallogr. D Biol. Crystallogr.* **69**, 1204–1214 (2013).

16. R. Keegan, M. Wojdyr, G. Winter, A. Ashton, DIMPLE: a difference map pipeline for the rapid screening of crystals on the beamline. *Acta Crystallogr. A Found. Adv.* **71**, s18–s18 (2015).

17. M. D. Winn, *et al.*, Overview of the CCP4 suite and current developments. *Acta Crystallogr. D Biol. Crystallogr.* **67**, 235–242 (2011).

18. N. M. Pearce, *et al.*, A multi-crystal method for extracting obscured crystallographic states from conventionally uninterpretable electron density. *Nat. Commun.* **8**, 15123 (2017).

19. P. Emsley, B. Lohkamp, W. G. Scott, K. Cowtan, Features and development of Coot. *Acta Crystallogr. D Biol. Crystallogr.* **66**, 486–501 (2010).

20. N. W. Moriarty, R. W. Grosse-Kunstleve, P. D. Adams, electronic Ligand Builder and Optimization Workbench (eLBOW): a tool for ligand coordinate and restraint generation. *Acta Crystallogr. D Biol. Crystallogr.* **65**, 1074–1080 (2009).

21. F. Long, *et al.*, AceDRG: a stereochemical description generator for ligands. *Acta Crystallogr D Struct Biol* **73**, 112–122 (2017).

22. L. L. C. Schrödinger, *LigPrep*.

23. P. V. Afonine, *et al.*, Towards automated crystallographic structure refinement with phenix.refine. *Acta Crystallogr. D Biol. Crystallogr.* **68**, 352–367 (2012).

24. M. Schuller, *et al.*, Fragment Binding to the Nsp3 Macrodomain of SARS-CoV-2 Identified Through Crystallographic Screening and Computational Docking. *bioRxiv* (2020) https:/doi.org/10.1101/2020.11.24.393405.

25. R. Sharifi, *et al.*, Deficiency of terminal ADP-ribose protein glycohydrolase TARG1/C6orf130 in neurodegenerative disease. *EMBO J.* **32**, 1225–1237 (2013).

26. G. Jankevicius, *et al.*, A family of macrodomain proteins reverses cellular mono-ADP-ribosylation. *Nat. Struct. Mol. Biol.* **20**, 508–514 (2013).

27. D. Chen, *et al.*, Identification of macrodomain proteins as novel O-acetyl-ADP-ribose deacetylases. *J. Biol. Chem.* **286**, 13261–13271 (2011).

28. X. Yang, *et al.*, Molecular basis for the MacroD1-mediated hydrolysis of ADP-ribosylation. *DNA Repair* **94**, 102899 (2020).

29. M. Schuller, *et al.*, Discovery of a Selective Allosteric Inhibitor Targeting Macrodomain 2 of Polyadenosine-Diphosphate-Ribose Polymerase 14. *ACS Chem. Biol.* **12**, 2866–2874 (2017).

30. A. H. Forst, *et al.*, Recognition of mono-ADP-ribosylated ARTD10 substrates by ARTD8 macrodomains. *Structure* **21**, 462–475 (2013).
