# Supplementary material for: Iterative computational design and crystallographic screening identifies potent inhibitors targeting the Nsp3 macrodomain of SARS-CoV-2
Source: Proc Natl Acad Sci U S A. 2023 Jan 4;120(2):e2212931120. doi: 10.1073/pnas.2212931120 (PMC9926234; doi:10.1073/pnas.2212931120)

Figure	# tested	# active	# inactive	% active	actives	inactives
2	13	7	6	53.8	Z9604, Z8515, Z8531, Z8539, Z8572, Z8580, Z8585	Z3628, Z8554, Z8569, Z8593, Z8598, Z8614
3	18	8	10	44.44	Z8539_0029, Z8539_0027, Z8539_0030, Z8539_0046, Z8539_0041, Z8539_0014, Z8539_0002, Z8539_0023	Z8539_0026, Z8539_0025, Z8539_0037, Z8539_0038, Z8539_0039, Z8539_0009, Z8539_0076, Z8539_0007, Z8539_0011, Z8539_0006
5, all	123	38	85	30.9	See by-screen below	
5, screen 1	78	30	48	38.4	Z9020, Z1628, Z1635, Z3291, Z4141, Z5722, Z5356, Z2051, Z0856, Z9550, Z4305, Z4911, Z1027, Z9920, Z5531, Z1015, Z3896, Z8074, Z8186, Z3271, Z5972, Z0893, Z0078, Z4066, Z9652, Z5086, Z4620, Z3789, Z7873, Z1121	Z8637, Z6511, Z8648, Z5167, Z6395, Z2024, Z4135, Z0329, Z4508, Z9710, Z1086, Z5547, Z2380, Z5505, Z8885, Z7864, Z7665, Z3280, Z7041, Z7570, Z6891, Z8680, Z6019, Z7655, Z7766, Z3167, Z5728, Z8387, Z2995, Z9310, Z1211, Z3700, Z5262, Z0132, Z0953, Z4827, Z6028, Z0288, Z1347, Z6468, Z5289, Z7156, Z1186, Z0828, Z6923, Z7450, Z9572, Z6511
5, screen 2	45	8	37	17.8	F2804, Z8207, Z3432, F6831, R7335, Z9274, F4769, R1104	Z1964, R6134, F9046, F7028, Z4196, F0346, Z8835, Z8619, F8284, Z3247, F9192, R3575, R8538, R5324, Z1747, Z8095, F4848, Z4464, R4764, R2032, Z9614, Z1110, Z9028, R8908, R2488, Z4273, Z0063, Z7098, R4401, Z1527, Z6402, Z2483, Z6960, R8539, R8407, Z6512, R0658. (Not tested: Z1314)
7	9	5	4	55.56	LL1_0023, LL1_0025, LL1_0018, LL1_0014, LL1_0027	LL1_0016, LL1_0010, LL1_0005, LL1_0017

Actives are defined as any compound for which both of the following conditions were met: (1) ΔTma and compound concentration were positively correlated by Spearman, with a p-value less than or equal to 0.05, and thermal shift and (2) the maximum ΔTma was equal to or exceeded 0.5 ºC.

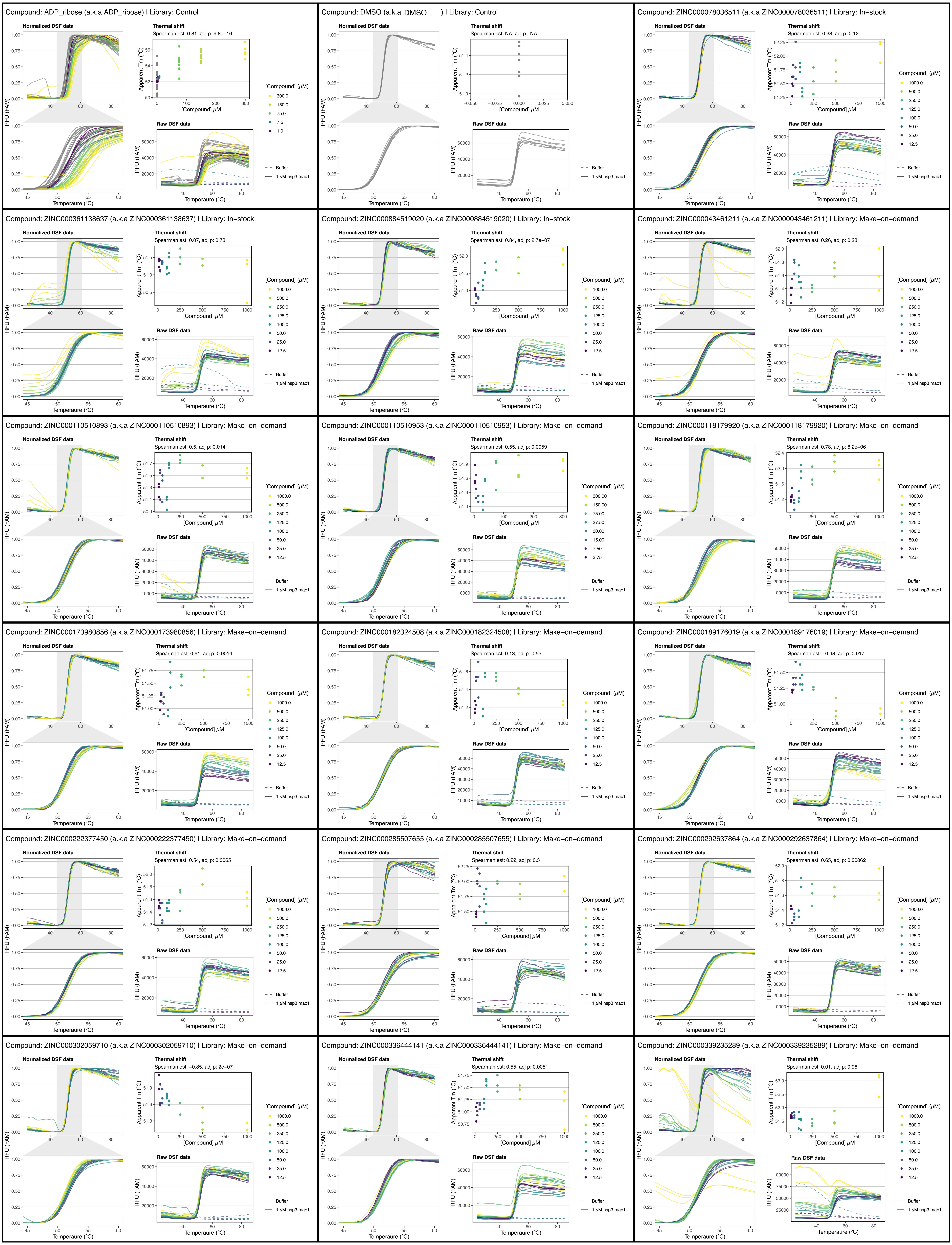
Compound: ADP_ribose (a.k.a ADP_ribose) | Library: Control
Normalized DSF data
Thermal shift
Spearman est: 0.81, adj p: 9.8e−16
Raw DSF data
Compound: DMSO (a.k.a DMSO) | Library: Control
Spearman est: NA, adj p: NA
Compound: ZINC000078036511 (a.k.a ZINC000078036511) | Library: In−stock
Spearman est: 0.33, adj p: 0.12
Compound: ZINC000361138637 (a.k.a ZINC000361138637) | Library: In−stock
Spearman est: 0.07, adj p: 0.73
Compound: ZINC000884519020 (a.k.a ZINC000884519020) | Library: In−stock
Spearman est: 0.84, adj p: 2.7e−07
Compound: ZINC000043461211 (a.k.a ZINC000043461211) | Library: Make−on−demand
Spearman est: 0.26, adj p: 0.23
Compound: ZINC000110510893 (a.k.a ZINC000110510893) | Library: Make−on−demand
Spearman est: 0.5, adj p: 0.014
Compound: ZINC000110510953 (a.k.a ZINC000110510953) | Library: Make−on−demand
Spearman est: 0.55, adj p: 0.0059
Compound: ZINC000118179920 (a.k.a ZINC000118179920) | Library: Make−on−demand
Spearman est: 0.78, adj p: 6.2e−06
Compound: ZINC000173980856 (a.k.a ZINC000173980856) | Library: Make−on−demand
Spearman est: 0.61, adj p: 0.0014
Compound: ZINC000182324508 (a.k.a ZINC000182324508) | Library: Make−on−demand
Spearman est: 0.13, adj p: 0.55
Compound: ZINC000189176019 (a.k.a ZINC000189176019) | Library: Make−on−demand
Spearman est: −0.48, adj p: 0.017
Compound: ZINC000222377450 (a.k.a ZINC000222377450) | Library: Make−on−demand
Spearman est: 0.54, adj p: 0.0065
Compound: ZINC000285507655 (a.k.a ZINC000285507655) | Library: Make−on−demand
Spearman est: 0.22, adj p: 0.3
Compound: ZINC000292637864 (a.k.a ZINC000292637864) | Library: Make−on−demand
Spearman est: 0.65, adj p: 0.00062
Compound: ZINC000302059710 (a.k.a ZINC000302059710) | Library: Make−on−demand
Spearman est: −0.85, adj p: 2e−07
Compound: ZINC000336444141 (a.k.a ZINC000336444141) | Library: Make−on−demand
Spearman est: 0.55, adj p: 0.0051
Compound: ZINC000339235289 (a.k.a ZINC000339235289) | Library: Make−on−demand
Spearman est: 0.01, adj p: 0.96
RFU (FAM)
Temperature (°C)
Apparent Tm (°C)
[Compound] μM
[Compound] (μM)
Buffer
1 μM nsp3 mac1

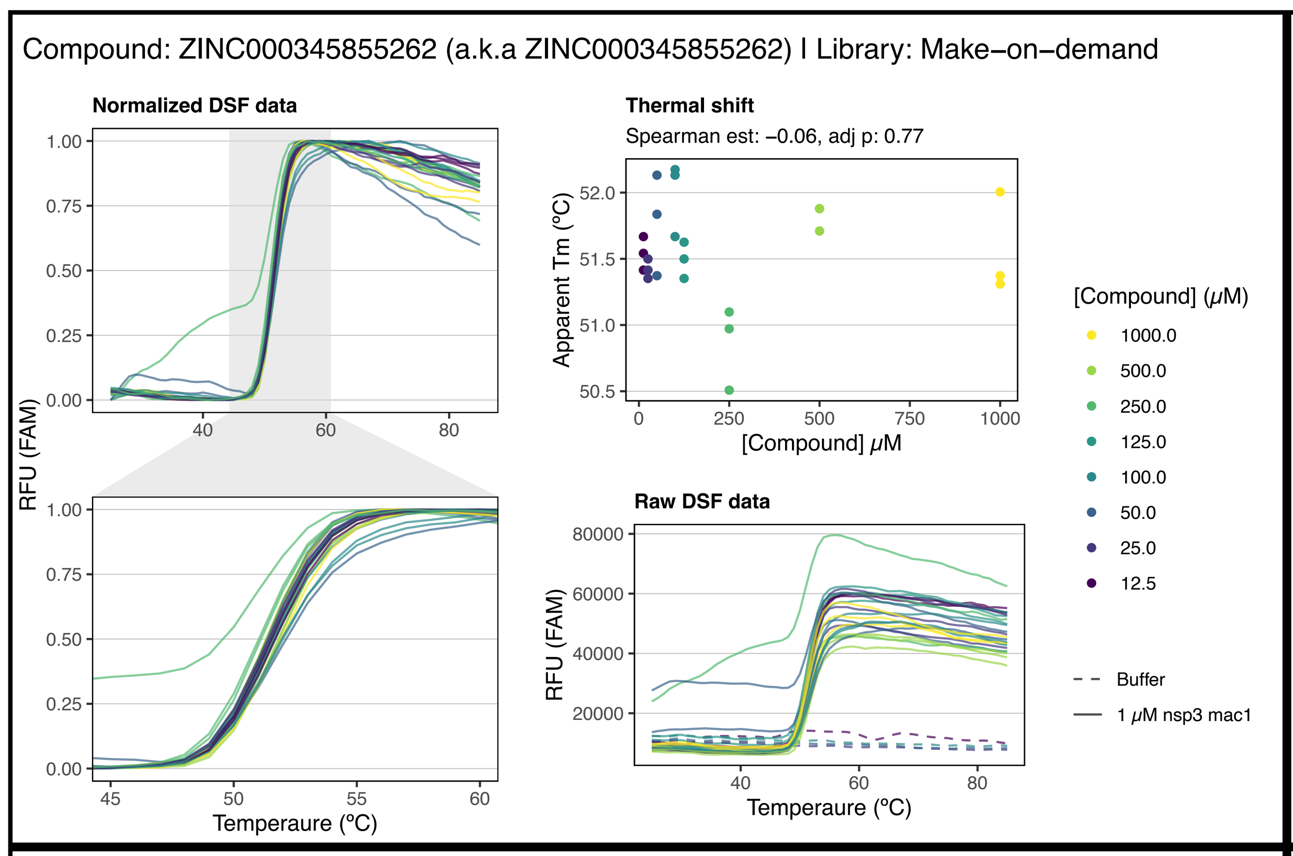
Compound: ZINC000345855262 (a.k.a ZINC000345855262) | Library: Make−on−demand
Normalized DSF data
Thermal shift
Spearman est: −0.06, adj p: 0.77
Apparent Tm (°C)
[Compound] μM
Raw DSF data
RFU (FAM)
Temperature (°C)
[Compound] (μM)
1000.0
500.0
250.0
125.0
100.0
50.0
25.0
12.5
Buffer
1 μM nsp3 mac1

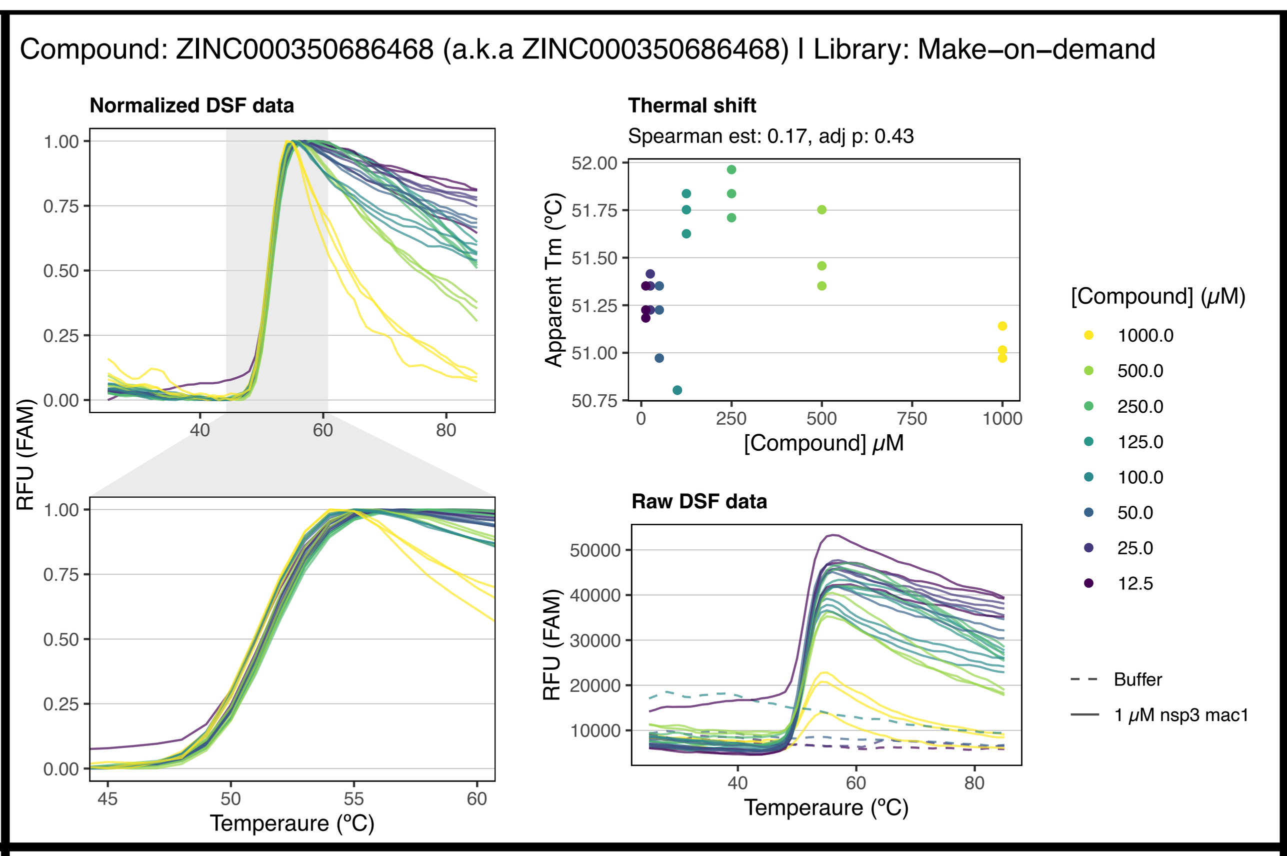
Compound: ZINC000350686468 (a.k.a ZINC000350686468) | Library: Make−on−demand
Normalized DSF data
Thermal shift
Spearman est: 0.17, adj p: 0.43
Apparent Tm (°C)
[Compound] μM
Raw DSF data
RFU (FAM)
Temperature (°C)
[Compound] (μM)
1000.0
500.0
250.0
125.0
100.0
50.0
25.0
12.5
Buffer
1 μM nsp3 mac1

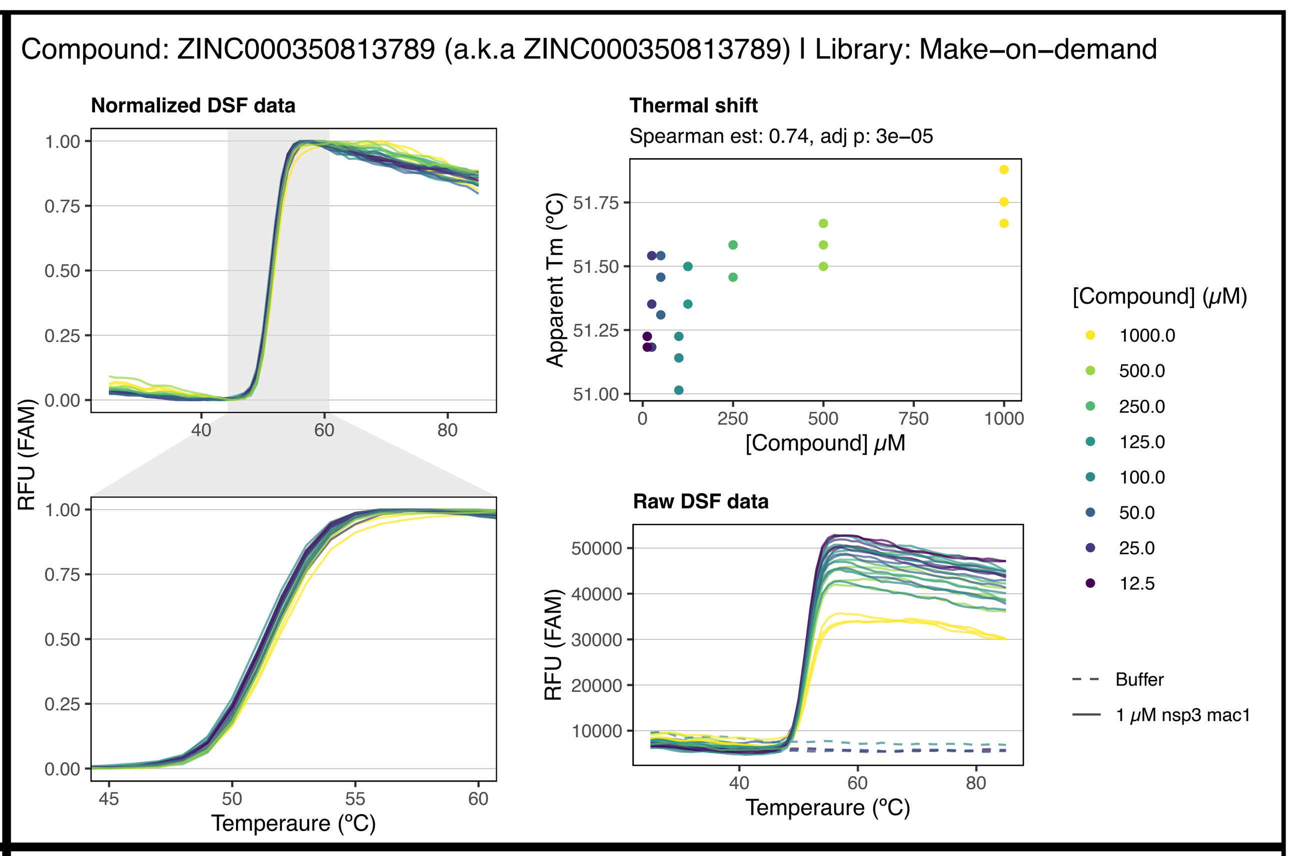
Compound: ZINC000350813789 (a.k.a ZINC000350813789) | Library: Make−on−demand
Normalized DSF data
Thermal shift
Spearman est: 0.74, adj p: 3e−05
Apparent Tm (°C)
[Compound] μM
Raw DSF data
RFU (FAM)
Temperature (°C)
[Compound] (μM)
1000.0
500.0
250.0
125.0
100.0
50.0
25.0
12.5
Buffer
1 μM nsp3 mac1

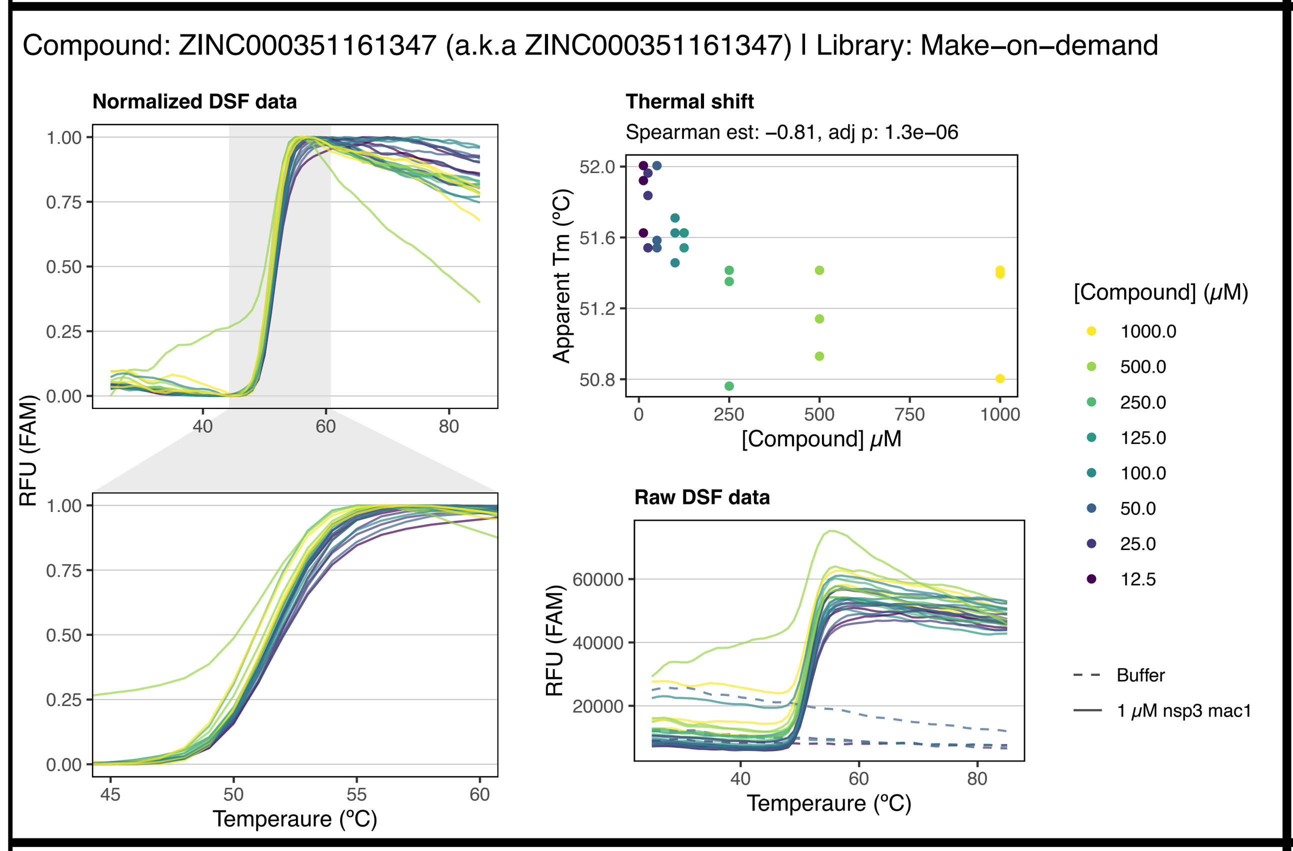
Compound: ZINC000351161347 (a.k.a ZINC000351161347) | Library: Make−on−demand
Normalized DSF data
Thermal shift
Spearman est: −0.81, adj p: 1.3e−06
Apparent Tm (°C)
[Compound] μM
Raw DSF data
RFU (FAM)
Temperature (°C)
[Compound] (μM)
1000.0
500.0
250.0
125.0
100.0
50.0
25.0
12.5
Buffer
1 μM nsp3 mac1

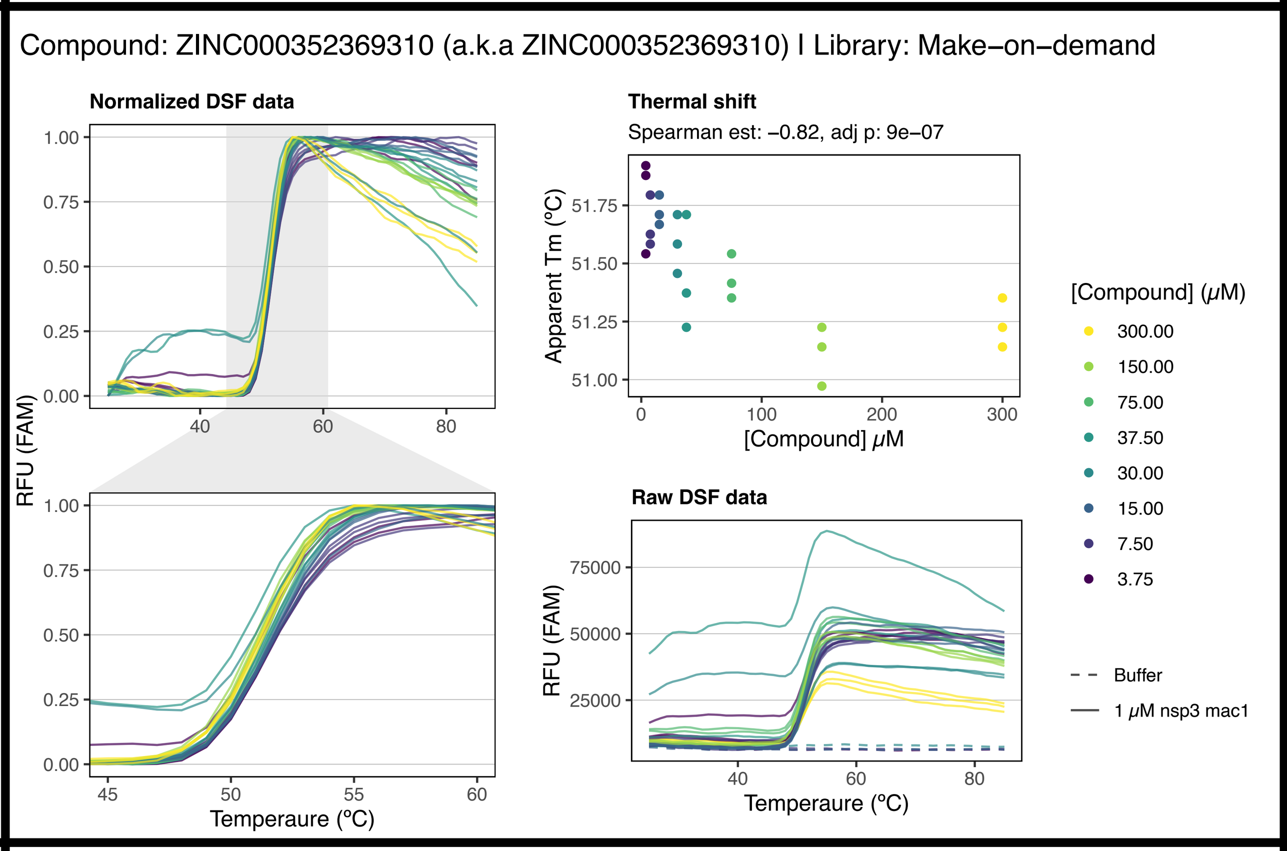
Compound: ZINC000352369310 (a.k.a ZINC000352369310) | Library: Make−on−demand
Normalized DSF data
Thermal shift
Spearman est: −0.82, adj p: 9e−07
Apparent Tm (°C)
[Compound] μM
Raw DSF data
RFU (FAM)
Temperature (°C)
[Compound] (μM)
300.00
150.00
75.00
37.50
30.00
15.00
7.50
3.75
Buffer
1 μM nsp3 mac1

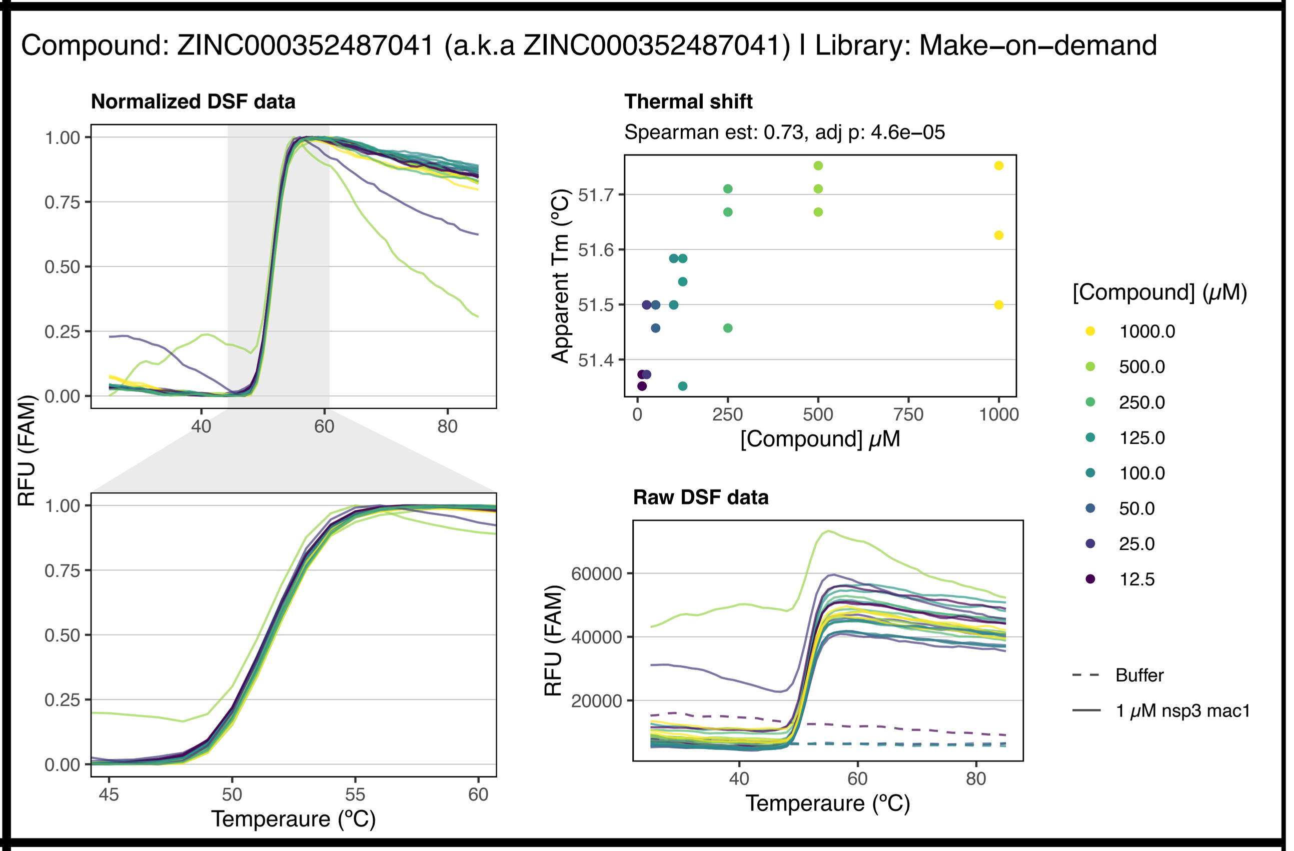
Compound: ZINC000352487041 (a.k.a ZINC000352487041) | Library: Make−on−demand
Normalized DSF data
Thermal shift
Spearman est: 0.73, adj p: 4.6e−05
Apparent Tm (°C)
[Compound] μM
Raw DSF data
RFU (FAM)
Temperature (°C)
[Compound] (μM)
1000.0
500.0
250.0
125.0
100.0
50.0
25.0
12.5
Buffer
1 μM nsp3 mac1

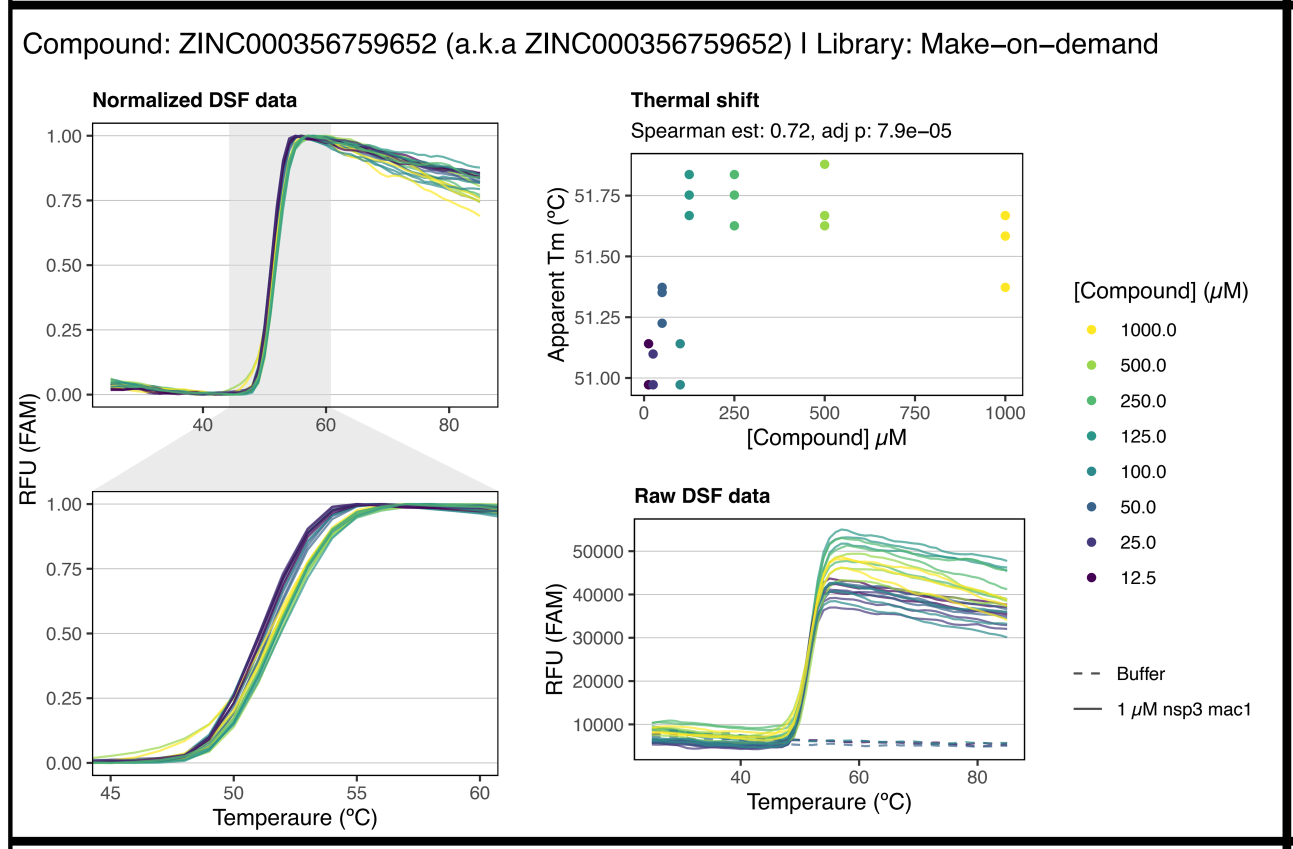
Compound: ZINC000356759652 (a.k.a ZINC000356759652) | Library: Make−on−demand
Normalized DSF data
Thermal shift
Spearman est: 0.72, adj p: 7.9e−05
Apparent Tm (°C)
[Compound] μM
Raw DSF data
RFU (FAM)
Temperature (°C)
[Compound] (μM)
1000.0
500.0
250.0
125.0
100.0
50.0
25.0
12.5
Buffer
1 μM nsp3 mac1

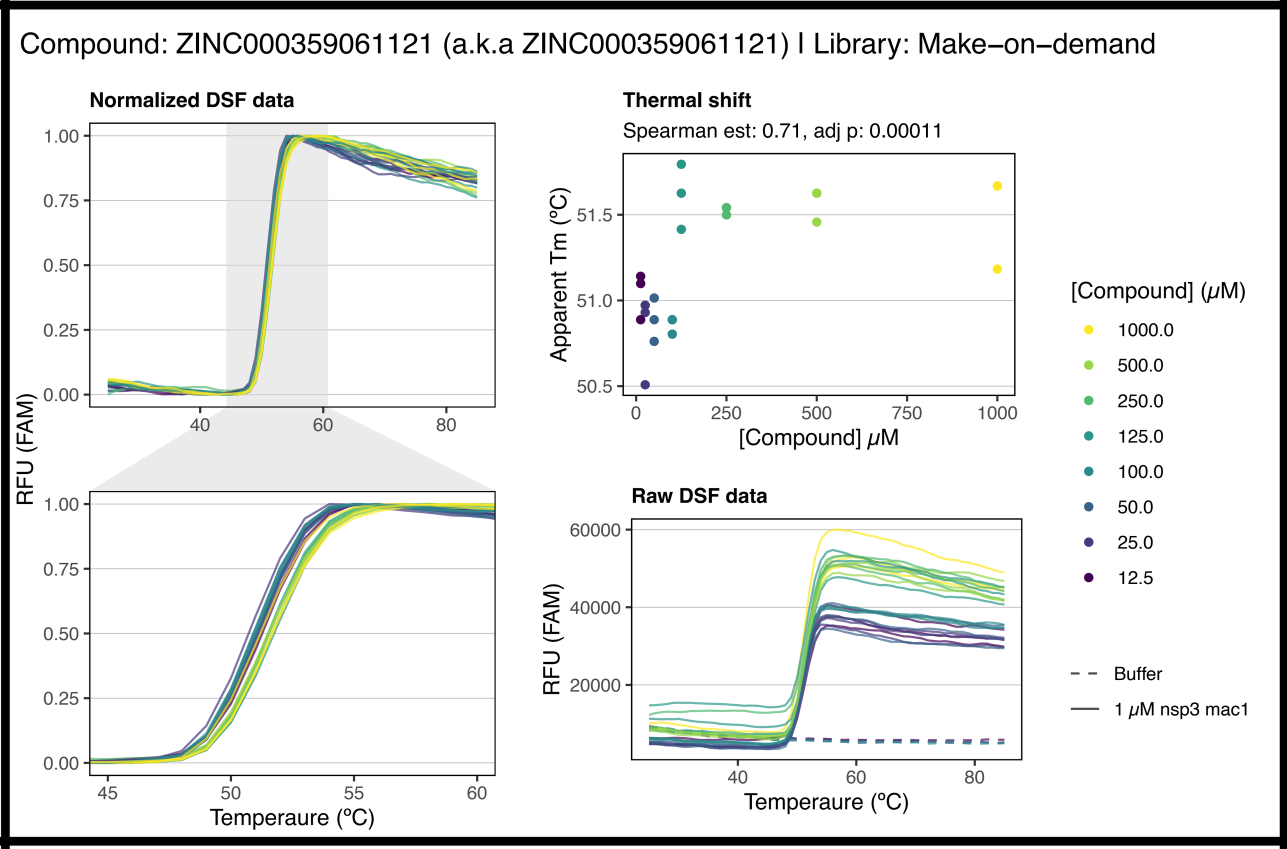
Compound: ZINC000359061121 (a.k.a ZINC000359061121) | Library: Make−on−demand
Normalized DSF data
Thermal shift
Spearman est: 0.71, adj p: 0.00011
Apparent Tm (°C)
[Compound] μM
Raw DSF data
RFU (FAM)
Temperature (°C)
[Compound] (μM)
1000.0
500.0
250.0
125.0
100.0
50.0
25.0
12.5
Buffer
1 μM nsp3 mac1

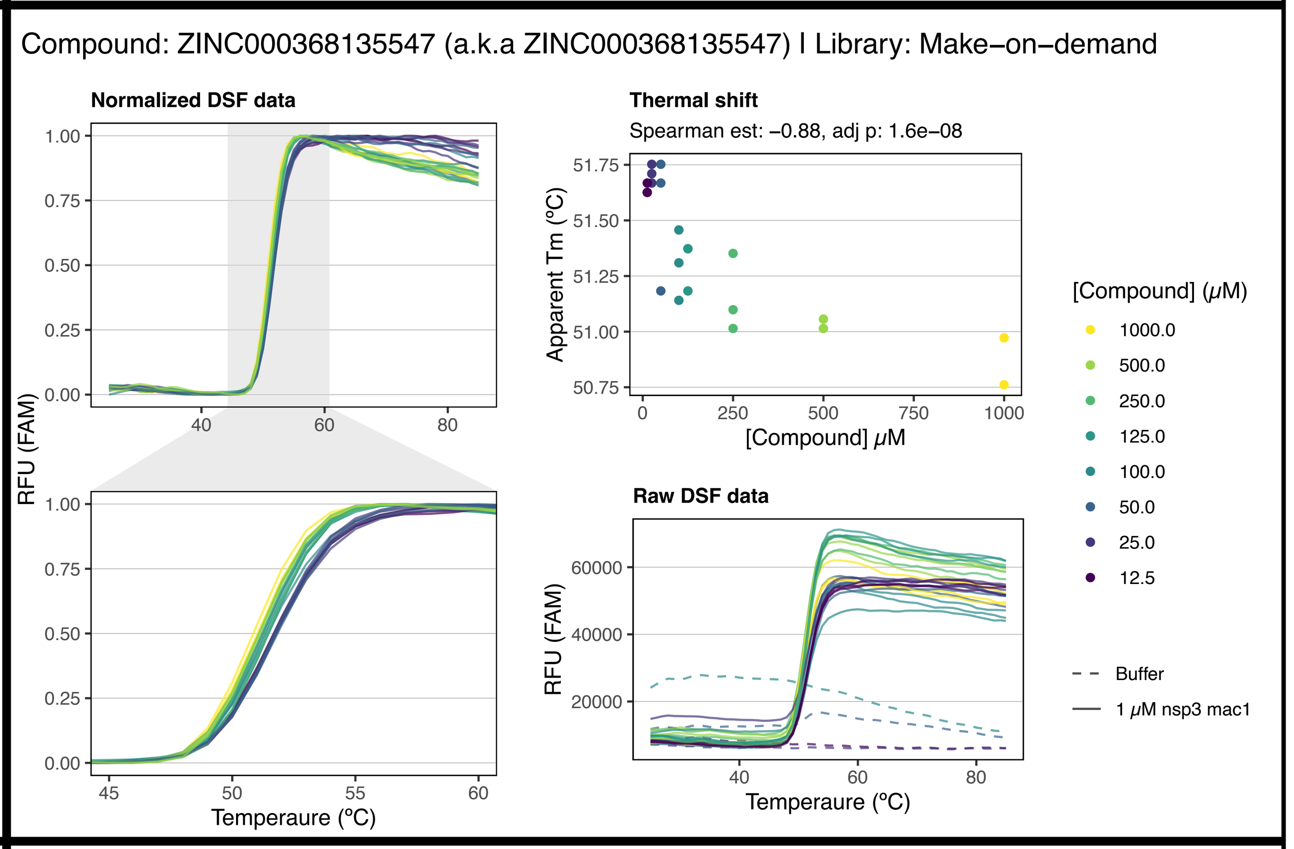
Compound: ZINC000368135547 (a.k.a ZINC000368135547) | Library: Make−on−demand
Normalized DSF data
Thermal shift
Spearman est: −0.88, adj p: 1.6e−08
Apparent Tm (°C)
[Compound] μM
Raw DSF data
RFU (FAM)
Temperature (°C)
[Compound] (μM)
1000.0
500.0
250.0
125.0
100.0
50.0
25.0
12.5
Buffer
1 μM nsp3 mac1

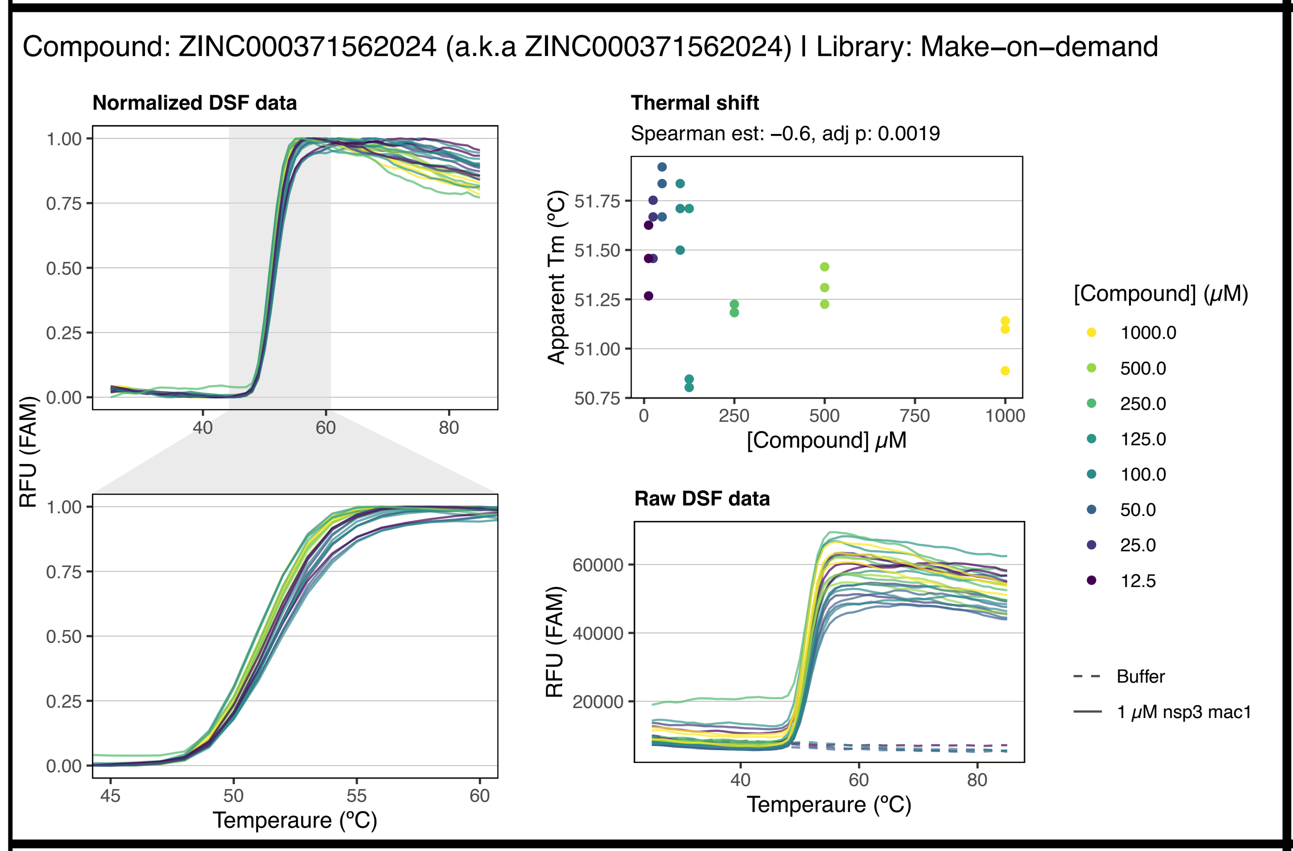
Compound: ZINC000371562024 (a.k.a ZINC000371562024) | Library: Make−on−demand
Normalized DSF data
Thermal shift
Spearman est: −0.6, adj p: 0.0019
Apparent Tm (°C)
[Compound] μM
Raw DSF data
RFU (FAM)
Temperature (°C)
[Compound] (μM)
1000.0
500.0
250.0
125.0
100.0
50.0
25.0
12.5
Buffer
1 μM nsp3 mac1

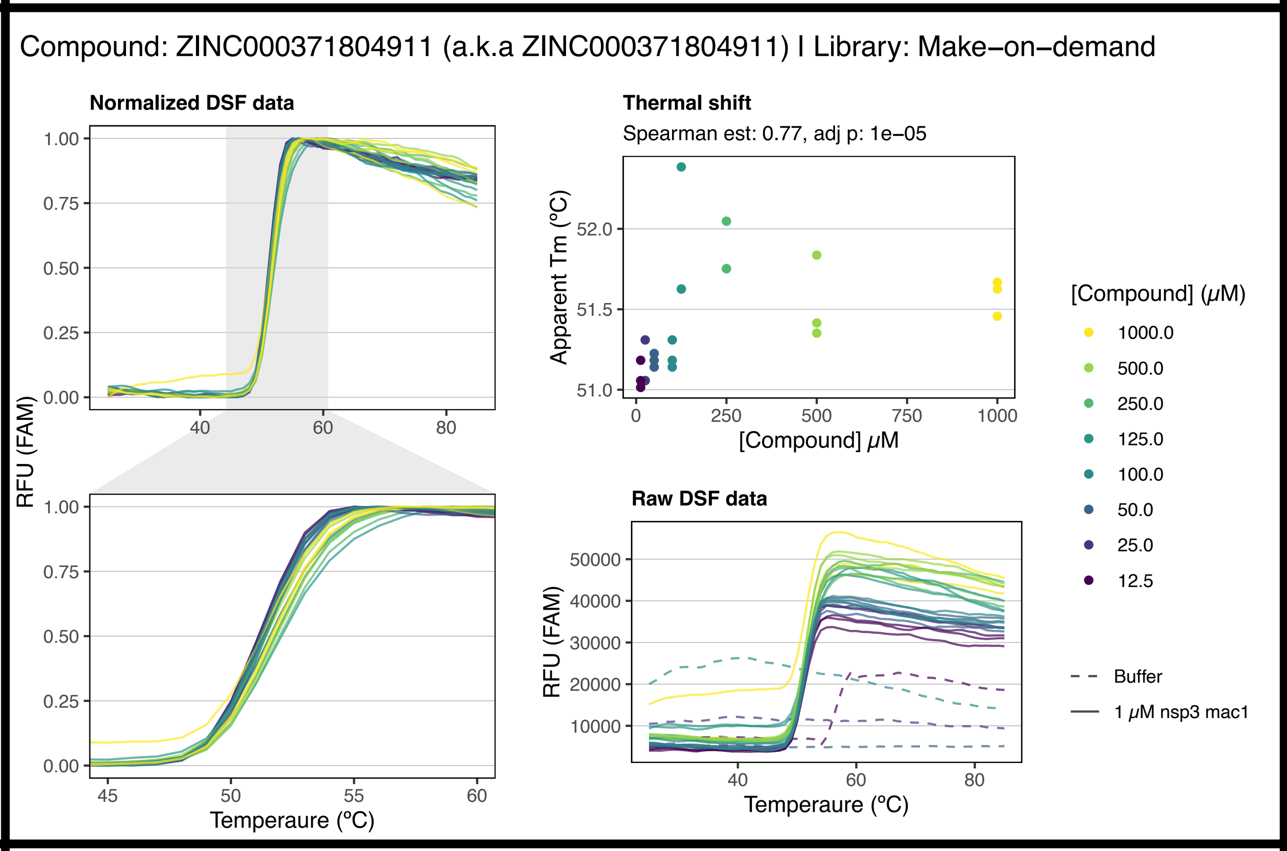
Compound: ZINC000371804911 (a.k.a ZINC000371804911) | Library: Make−on−demand
Normalized DSF data
Thermal shift
Spearman est: 0.77, adj p: 1e−05
Apparent Tm (°C)
[Compound] μM
Raw DSF data
RFU (FAM)
Temperature (°C)
[Compound] (μM)
1000.0
500.0
250.0
125.0
100.0
50.0
25.0
12.5
Buffer
1 μM nsp3 mac1

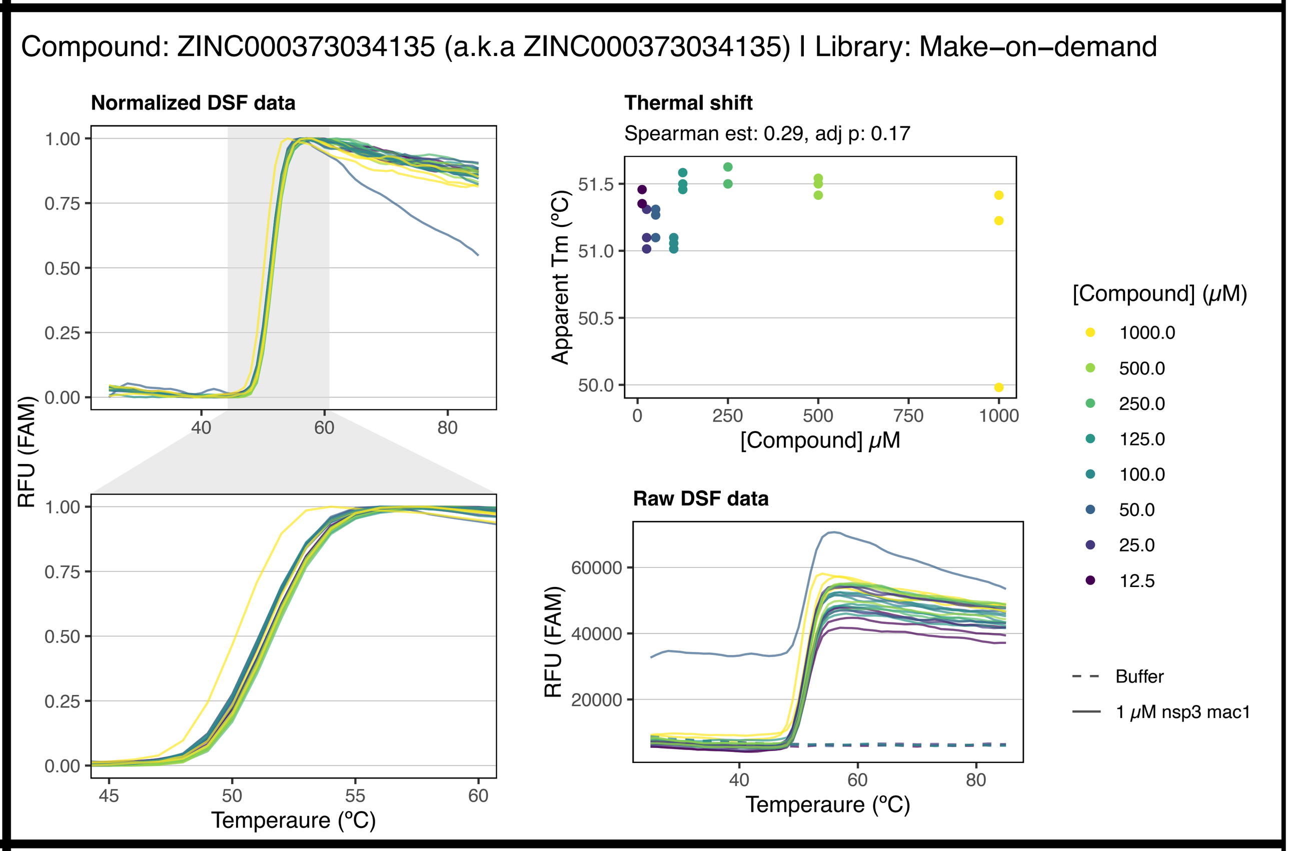
Compound: ZINC000373034135 (a.k.a ZINC000373034135) | Library: Make−on−demand
Normalized DSF data
Thermal shift
Spearman est: 0.29, adj p: 0.17
Apparent Tm (°C)
[Compound] μM
Raw DSF data
RFU (FAM)
Temperature (°C)
[Compound] (μM)
1000.0
500.0
250.0
125.0
100.0
50.0
25.0
12.5
Buffer
1 μM nsp3 mac1

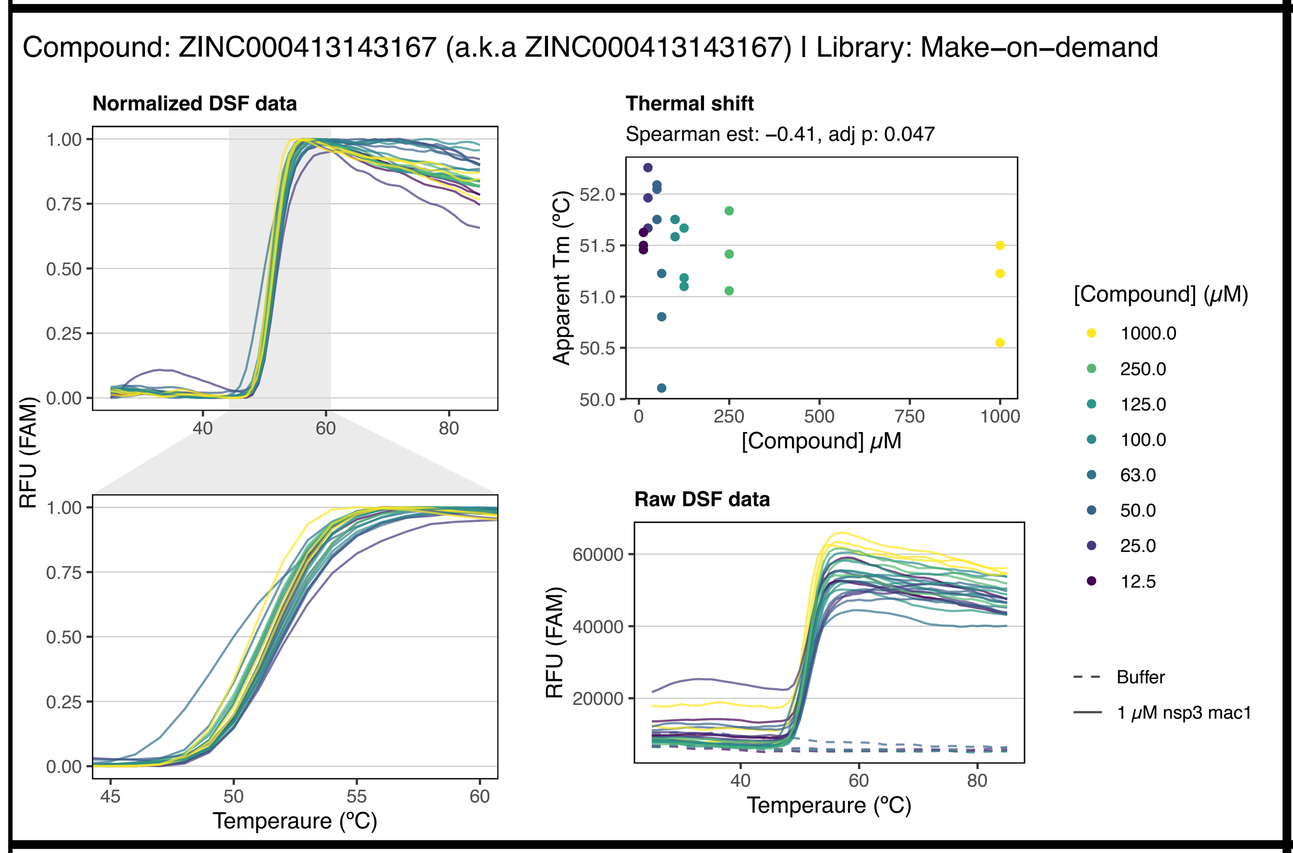
Compound: ZINC000413143167 (a.k.a ZINC000413143167) | Library: Make−on−demand
Normalized DSF data
Thermal shift
Spearman est: −0.41, adj p: 0.047
Apparent Tm (°C)
[Compound] μM
Raw DSF data
RFU (FAM)
Temperature (°C)
[Compound] (μM)
1000.0
250.0
125.0
100.0
63.0
50.0
25.0
12.5
Buffer
1 μM nsp3 mac1

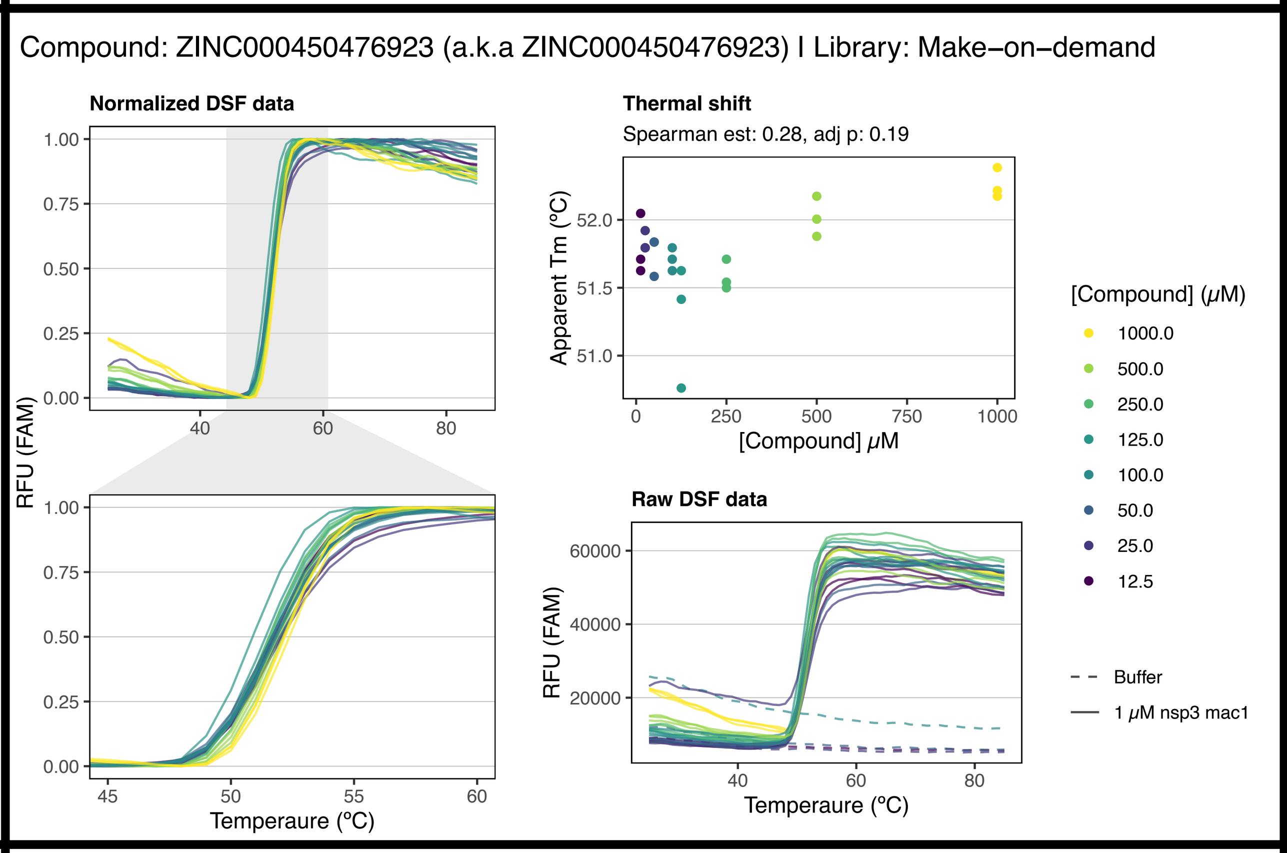
Compound: ZINC000450476923 (a.k.a ZINC000450476923) | Library: Make−on−demand
Normalized DSF data
Thermal shift
Spearman est: 0.28, adj p: 0.19
Apparent Tm (°C)
[Compound] μM
Raw DSF data
RFU (FAM)
Temperature (°C)
[Compound] (μM)
1000.0
500.0
250.0
125.0
100.0
50.0
25.0
12.5
Buffer
1 μM nsp3 mac1

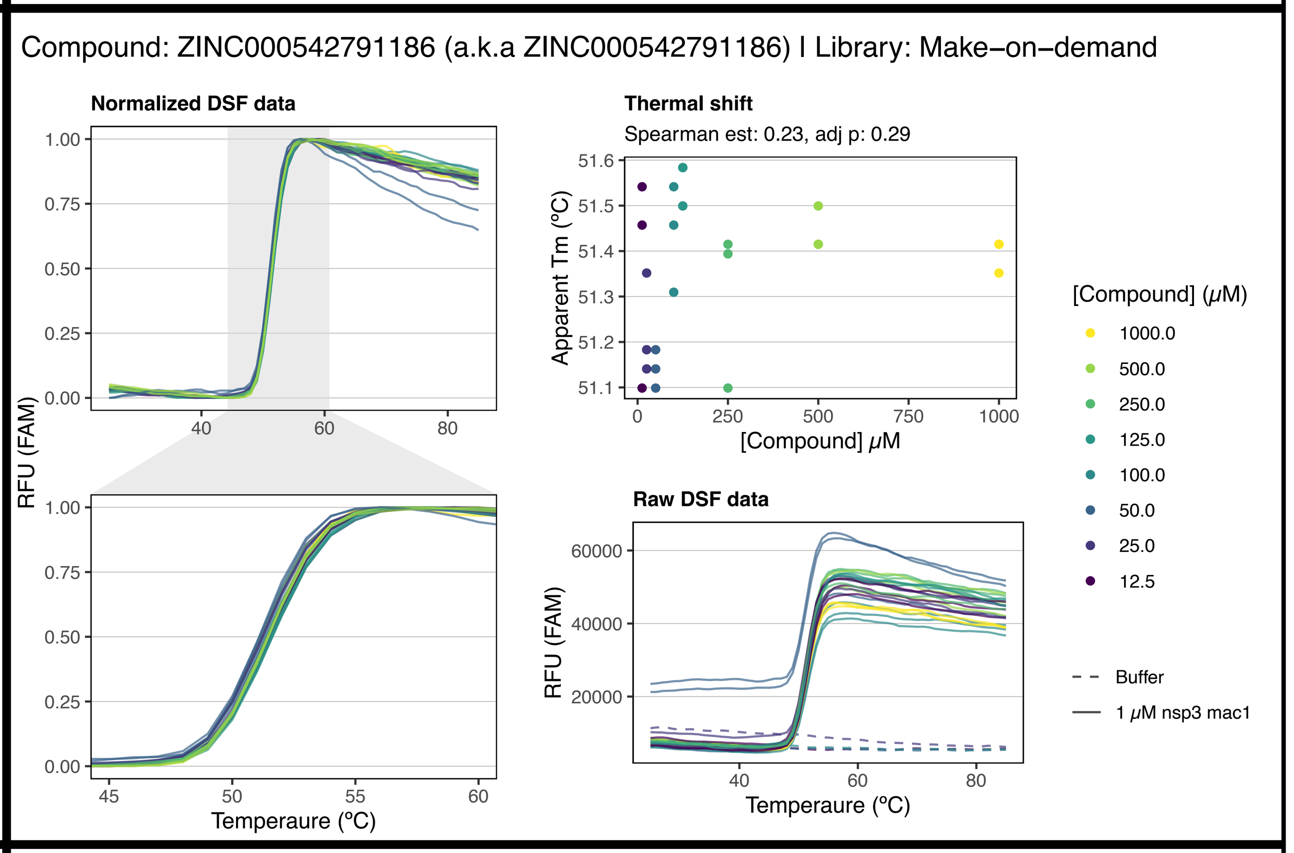
Compound: ZINC000542791186 (a.k.a ZINC000542791186) | Library: Make−on−demand
Normalized DSF data
Thermal shift
Spearman est: 0.23, adj p: 0.29
Apparent Tm (°C)
[Compound] μM
Raw DSF data
RFU (FAM)
Temperature (°C)
[Compound] (μM)
1000.0
500.0
250.0
125.0
100.0
50.0
25.0
12.5
Buffer
1 μM nsp3 mac1

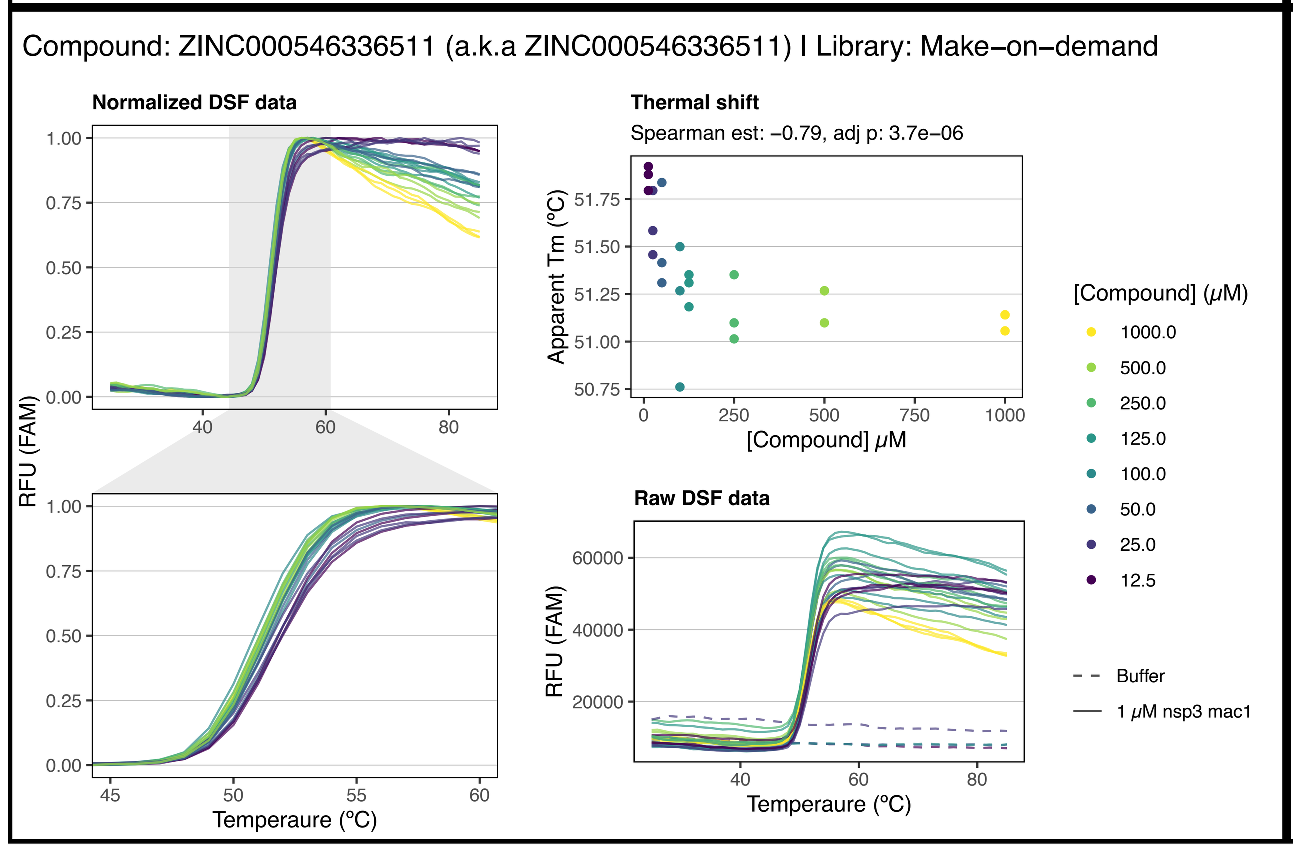
Compound: ZINC000546336511 (a.k.a ZINC000546336511) | Library: Make−on−demand
Normalized DSF data
Thermal shift
Spearman est: −0.79, adj p: 3.7e−06
Apparent Tm (°C)
[Compound] μM
Raw DSF data
RFU (FAM)
Temperature (°C)
[Compound] (μM)
1000.0
500.0
250.0
125.0
100.0
50.0
25.0
12.5
Buffer
1 μM nsp3 mac1

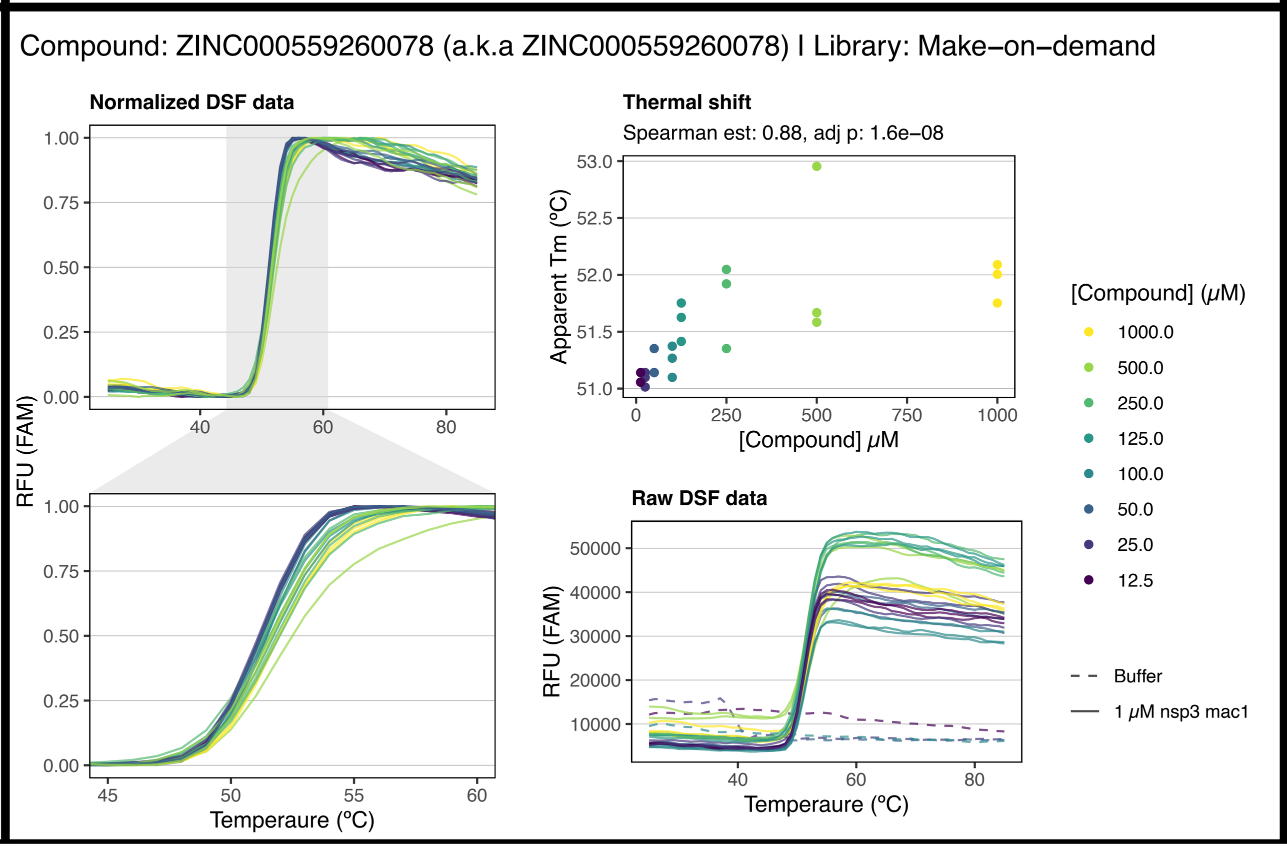
Compound: ZINC000559260078 (a.k.a ZINC000559260078) | Library: Make−on−demand
Normalized DSF data
Thermal shift
Spearman est: 0.88, adj p: 1.6e−08
Apparent Tm (°C)
[Compound] μM
Raw DSF data
RFU (FAM)
Temperature (°C)
[Compound] (μM)
1000.0
500.0
250.0
125.0
100.0
50.0
25.0
12.5
Buffer
1 μM nsp3 mac1

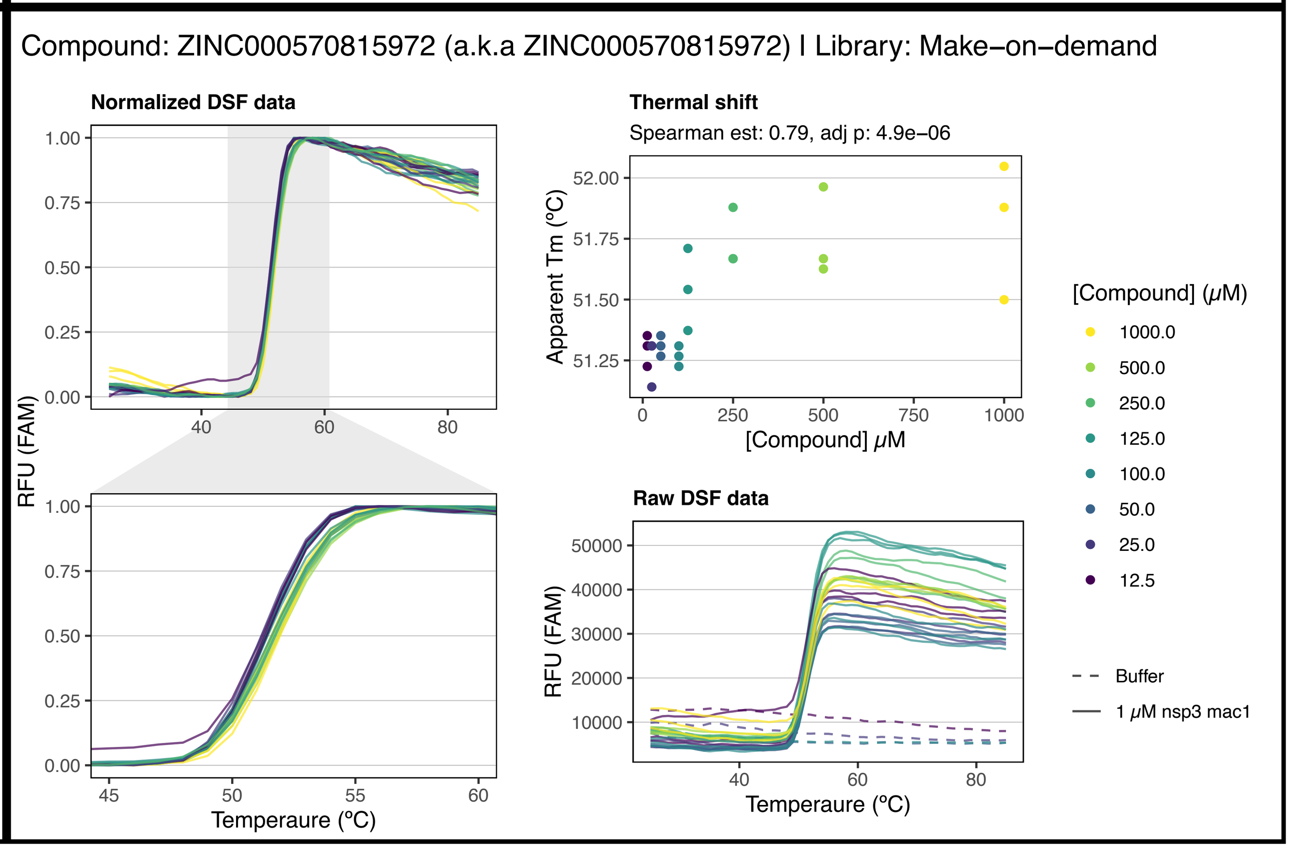
Compound: ZINC000570815972 (a.k.a ZINC000570815972) | Library: Make−on−demand
Normalized DSF data
Thermal shift
Spearman est: 0.79, adj p: 4.9e−06
Apparent Tm (°C)
[Compound] μM
Raw DSF data
RFU (FAM)
Temperature (°C)
[Compound] (μM)
1000.0
500.0
250.0
125.0
100.0
50.0
25.0
12.5
Buffer
1 μM nsp3 mac1

Compound: ZINC000579359572 (a.k.a ZINC000579359572) | Library: Make−on−demand

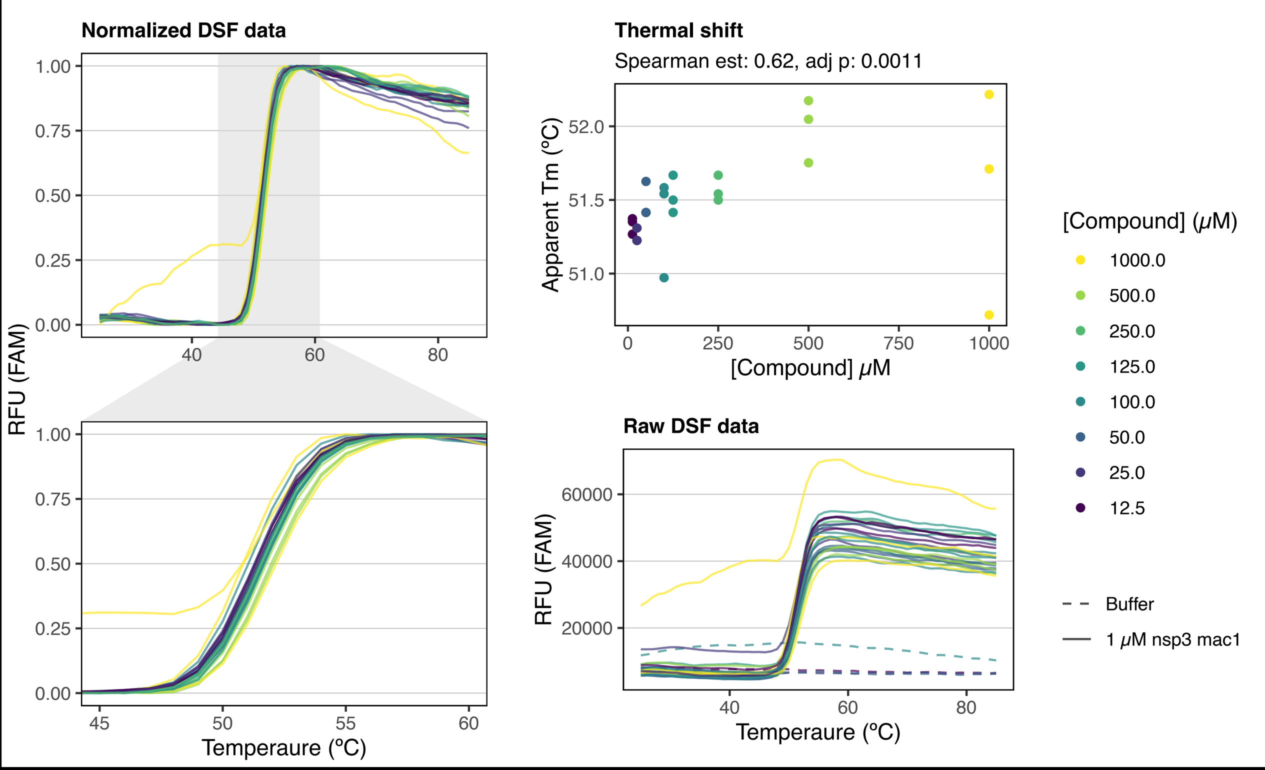

Compound: ZINC000641881628 (a.k.a ZINC000641881628) | Library: Make−on−demand

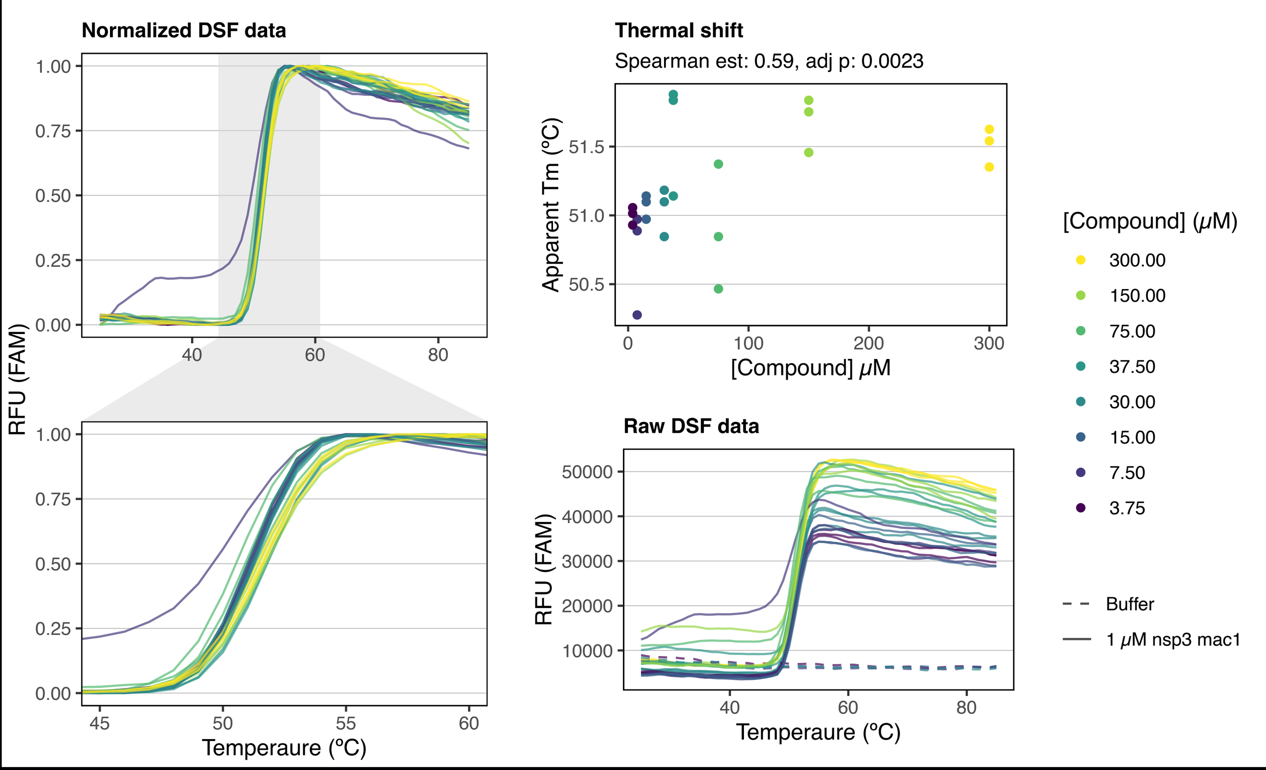

Compound: ZINC000642067873 (a.k.a ZINC000642067873) | Library: Make−on−demand

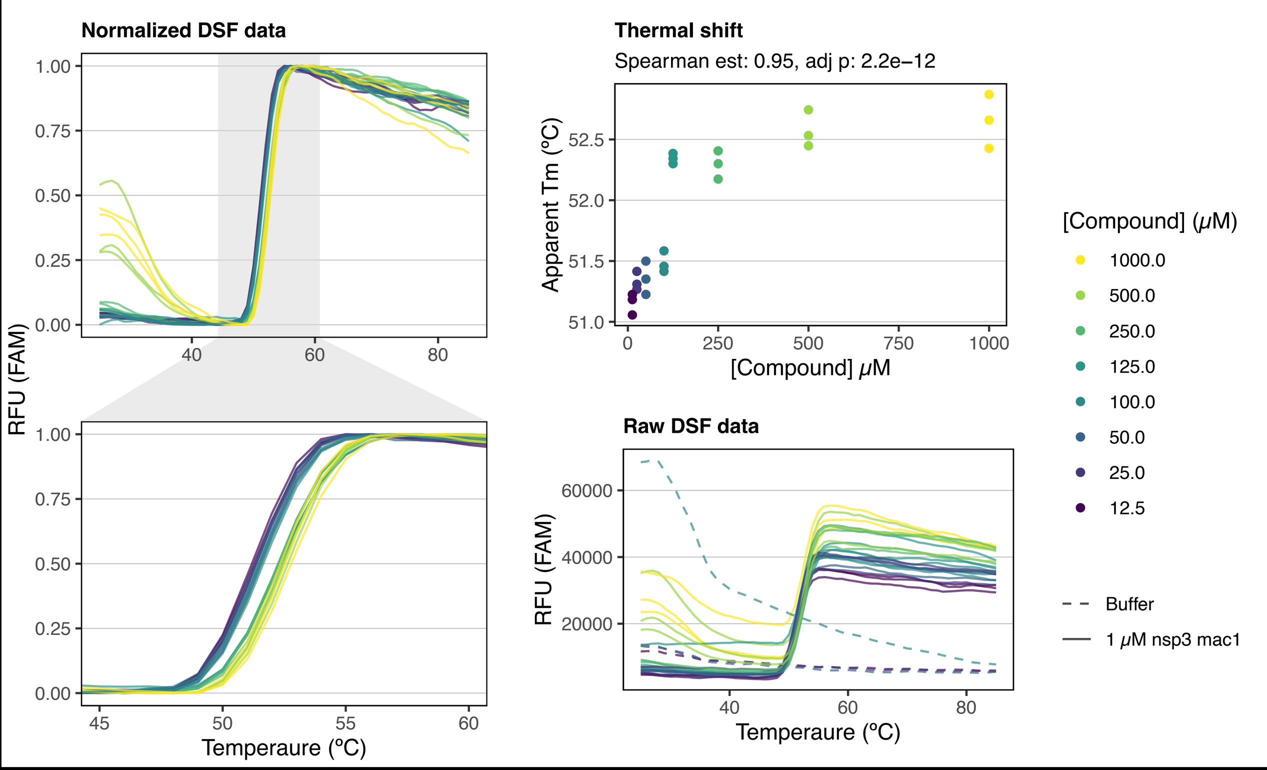

Compound: ZINC000649348648 (a.k.a ZINC000649348648) | Library: Make−on−demand

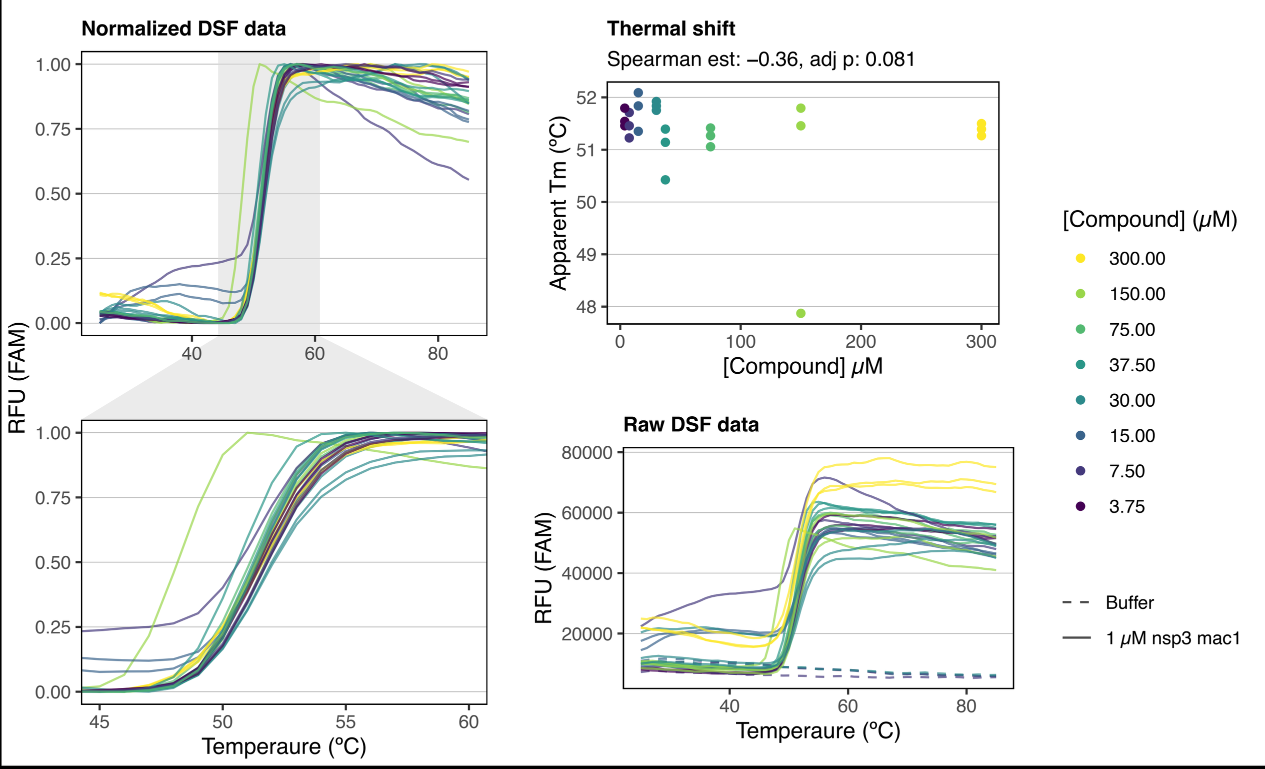

Compound: ZINC000664632051 (a.k.a ZINC000664632051) | Library: Make−on−demand

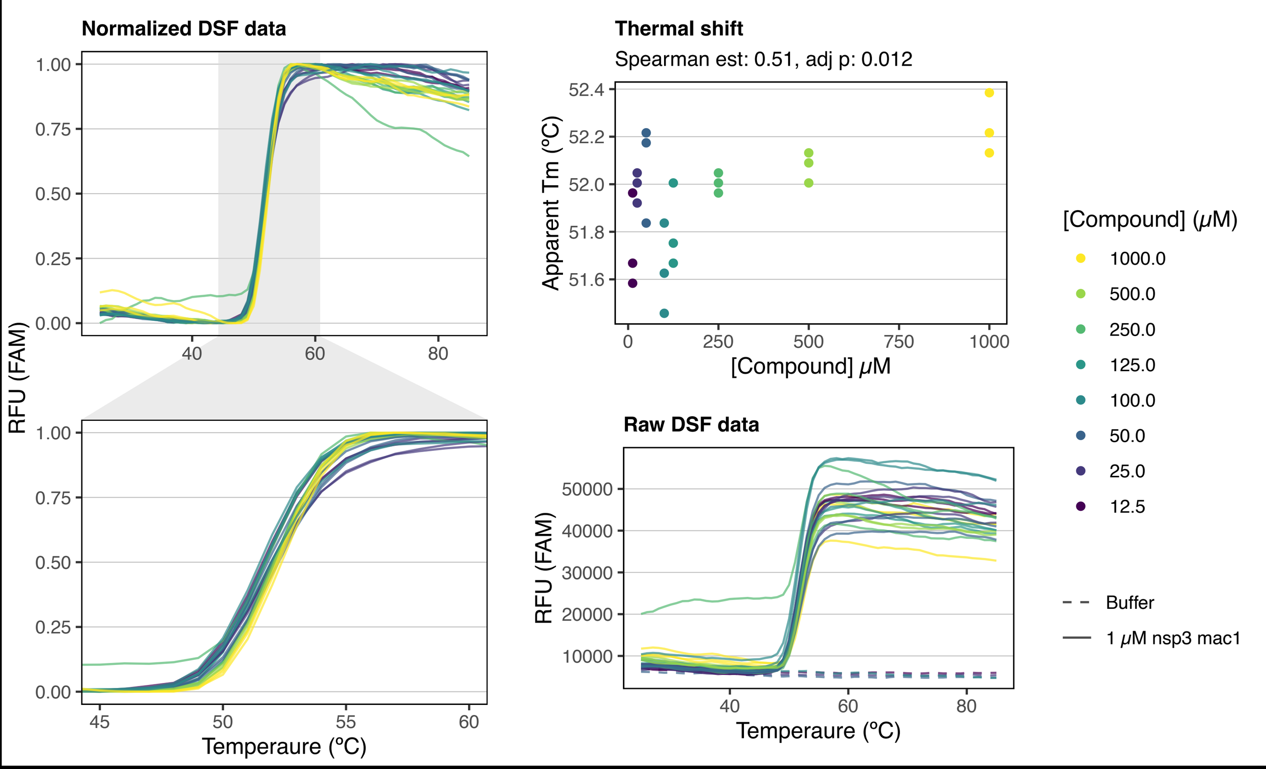

Compound: ZINC000679635167 (a.k.a ZINC000679635167) | Library: Make−on−demand

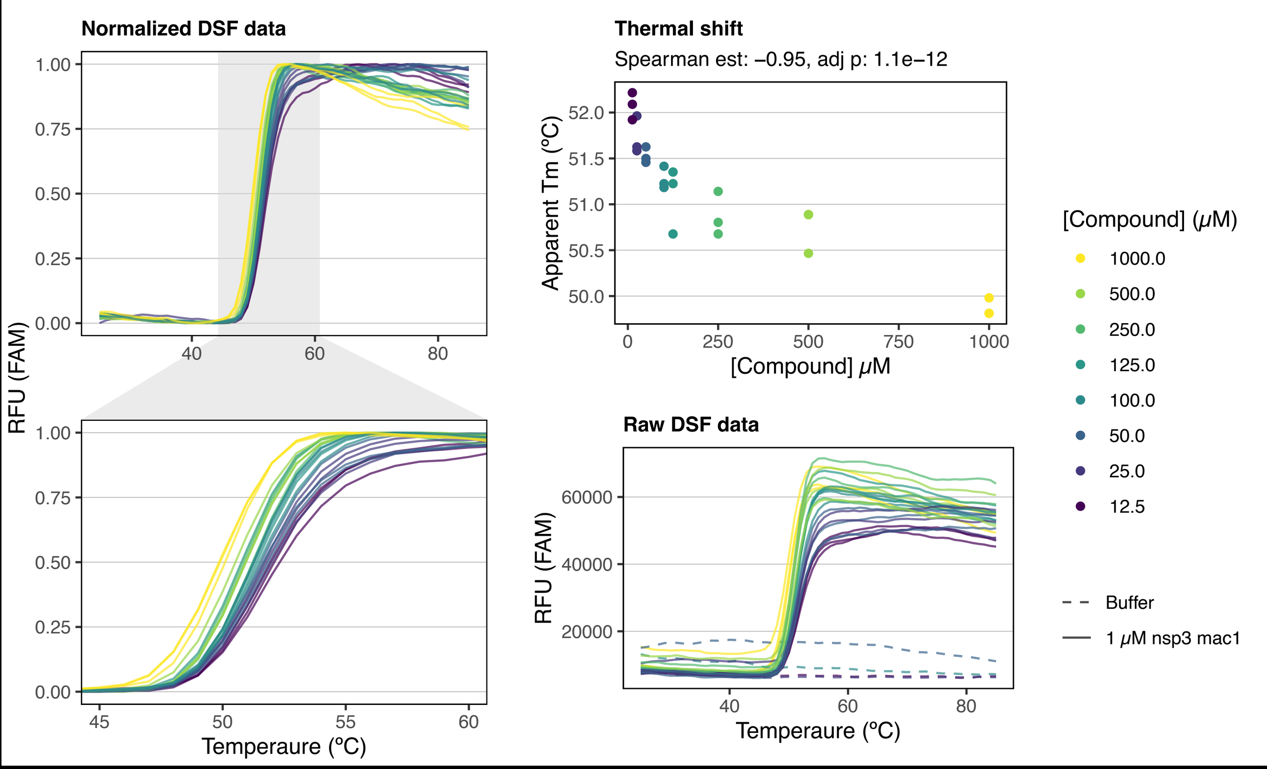

Compound: ZINC000681764827 (a.k.a ZINC000681764827) | Library: Make−on−demand

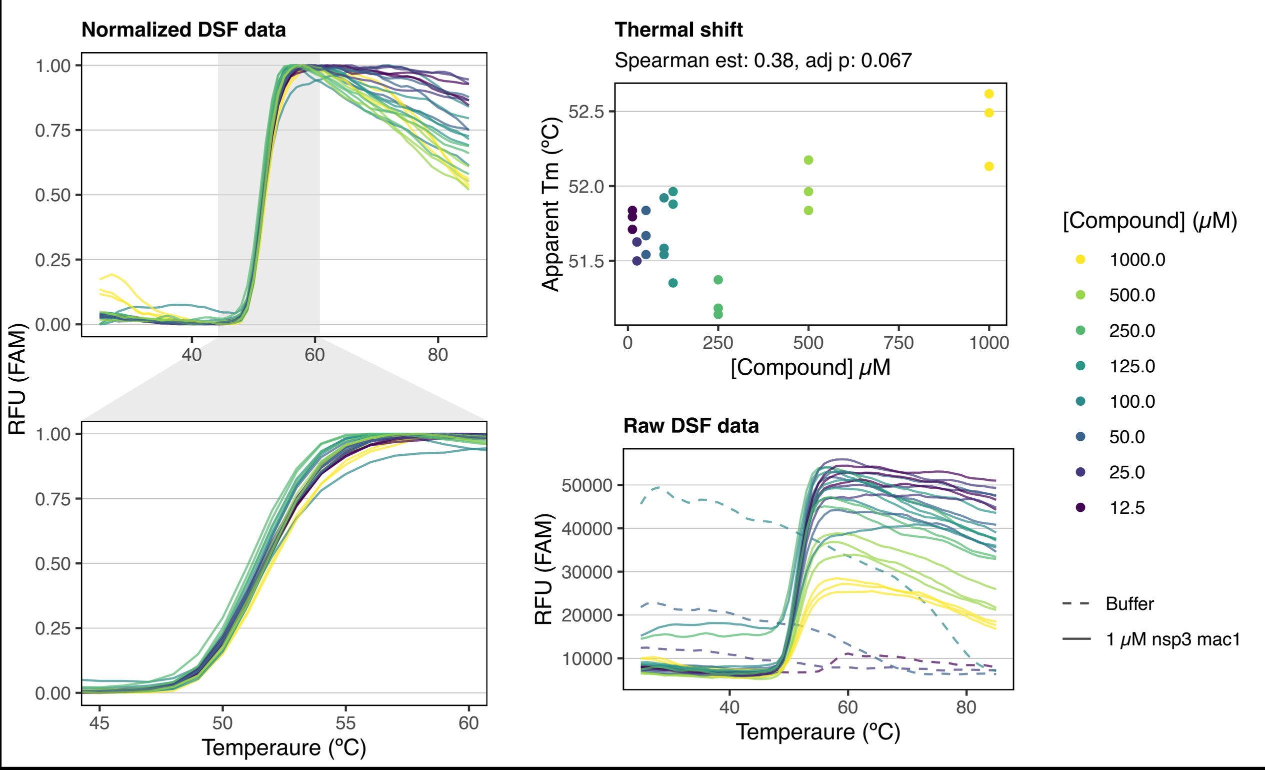

Compound: ZINC000733430132 (a.k.a ZINC000733430132) | Library: Make−on−demand

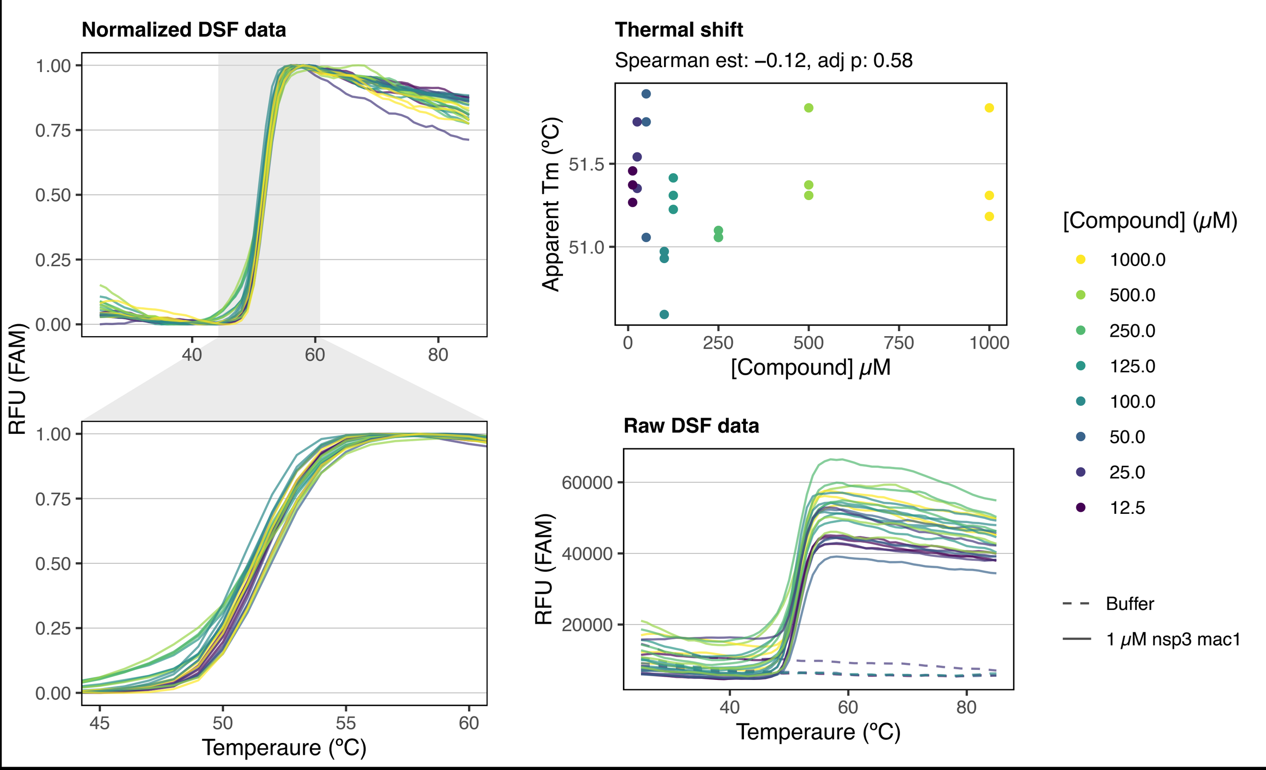

Compound: ZINC000745413896 (a.k.a ZINC000745413896) | Library: Make−on−demand

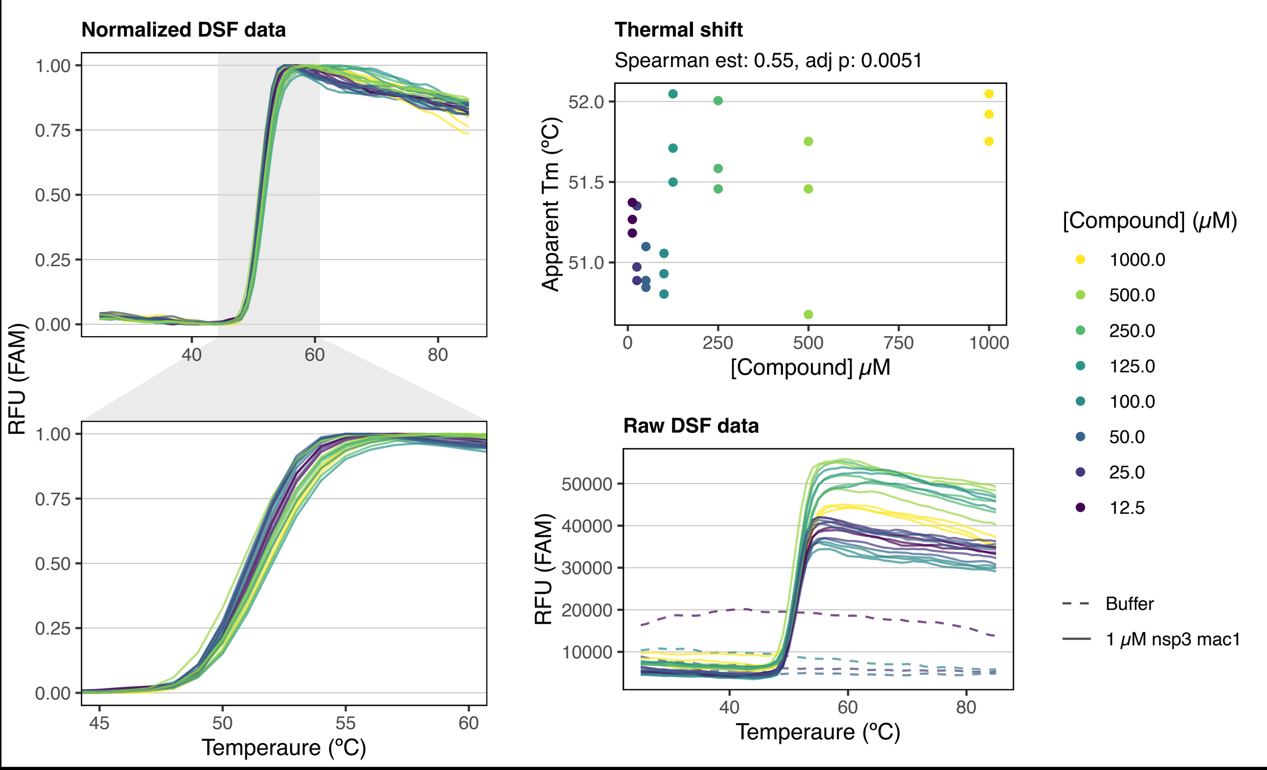

Compound: ZINC000772152995 (a.k.a ZINC000772152995) | Library: Make−on−demand

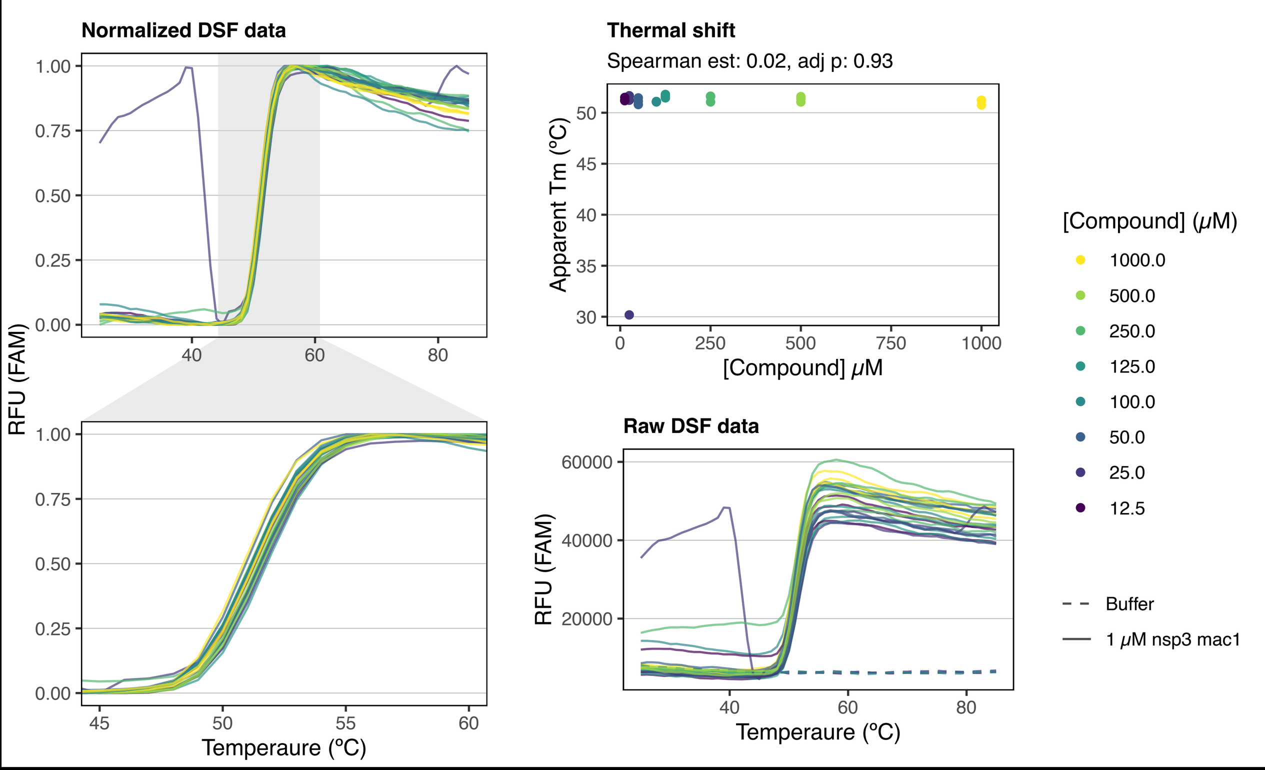

Compound: ZINC000773730329 (a.k.a ZINC000773730329) | Library: Make−on−demand

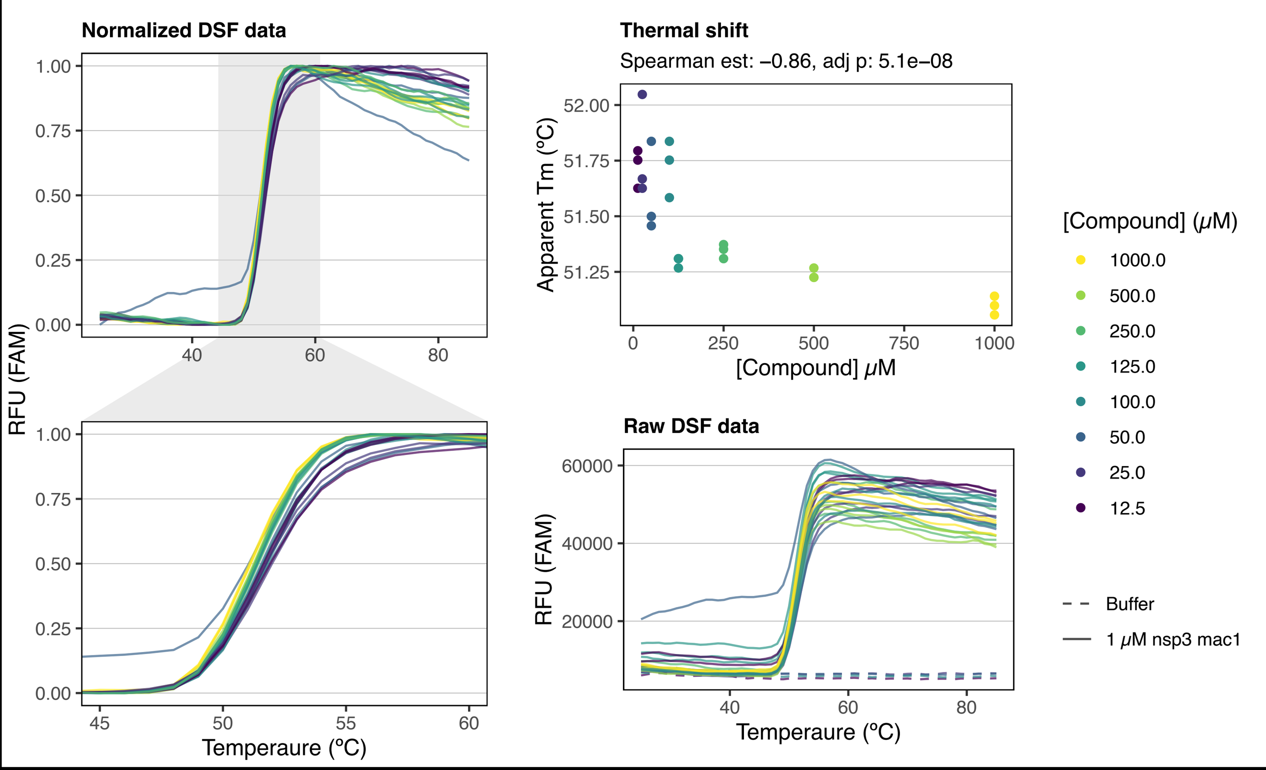

Compound: ZINC000779122380 (a.k.a ZINC000779122380) | Library: Make−on−demand

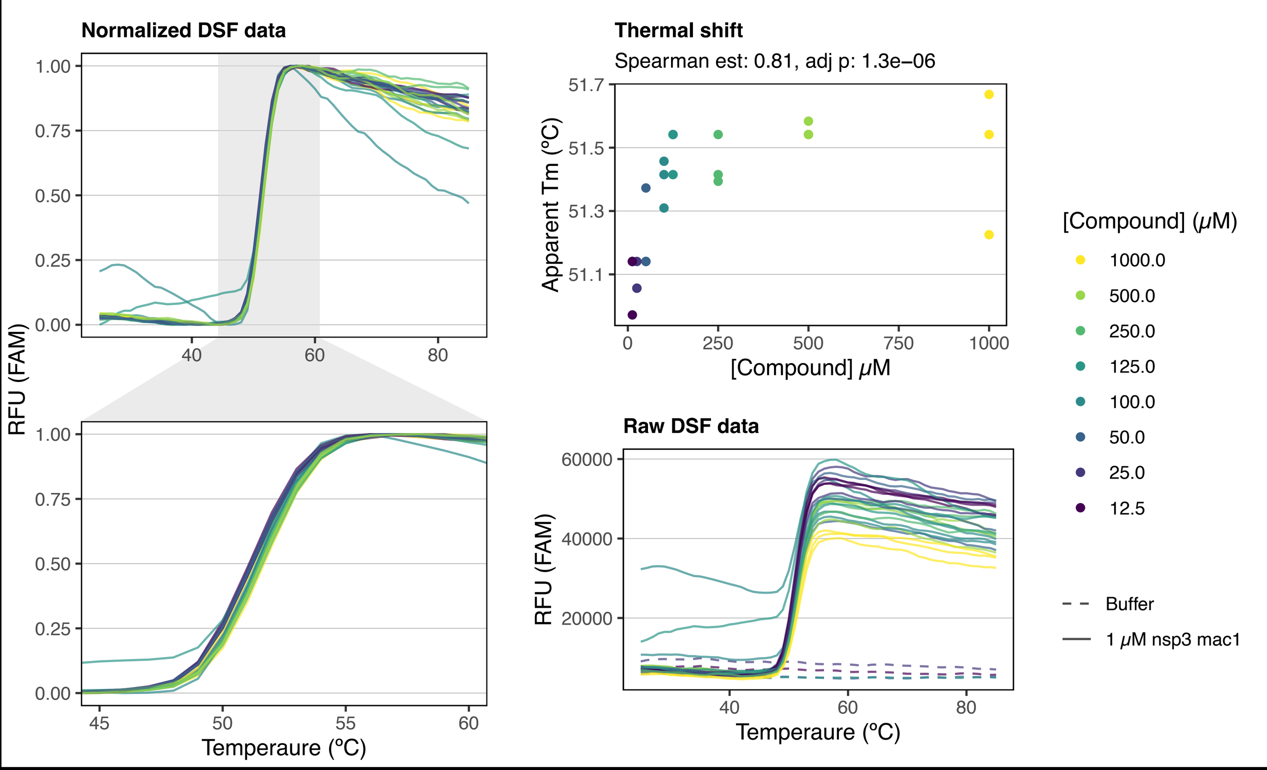

Compound: ZINC000798346395 (a.k.a ZINC000798346395) | Library: Make−on−demand

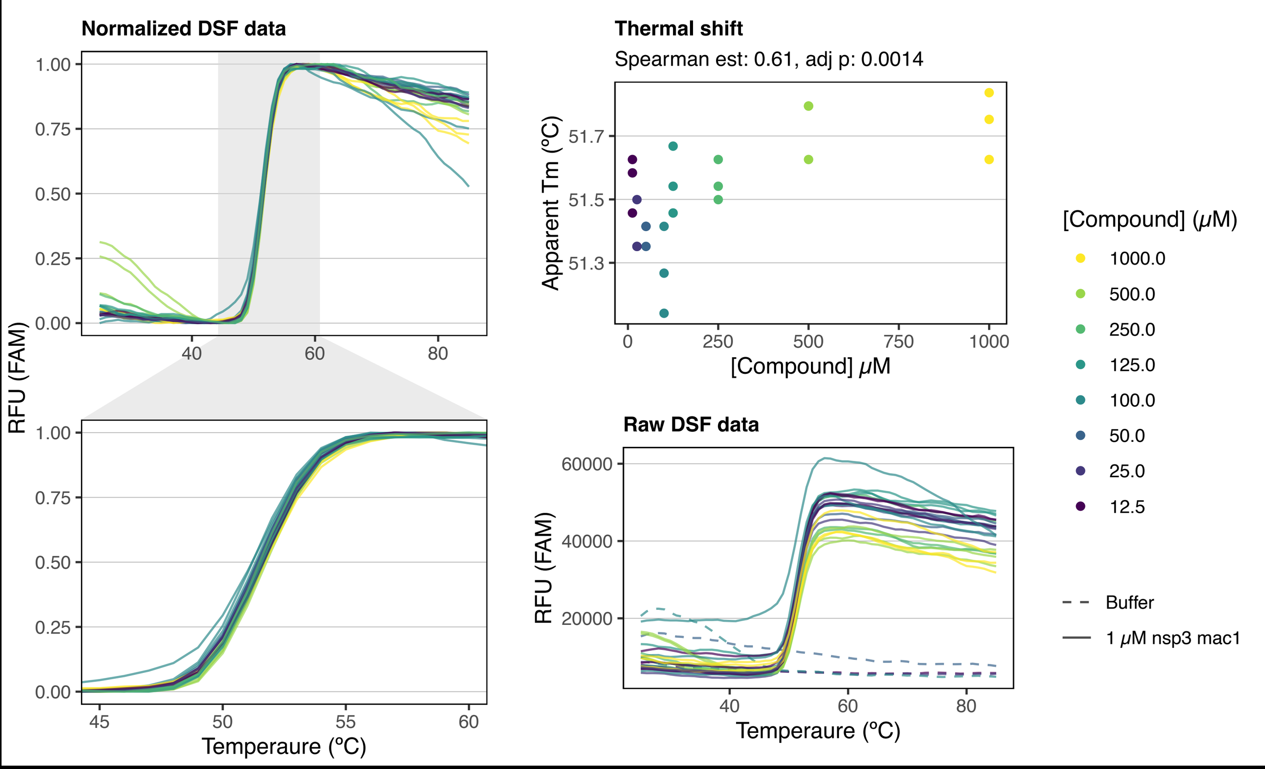

Compound: ZINC000820965086 (a.k.a ZINC000820965086) | Library: Make−on−demand

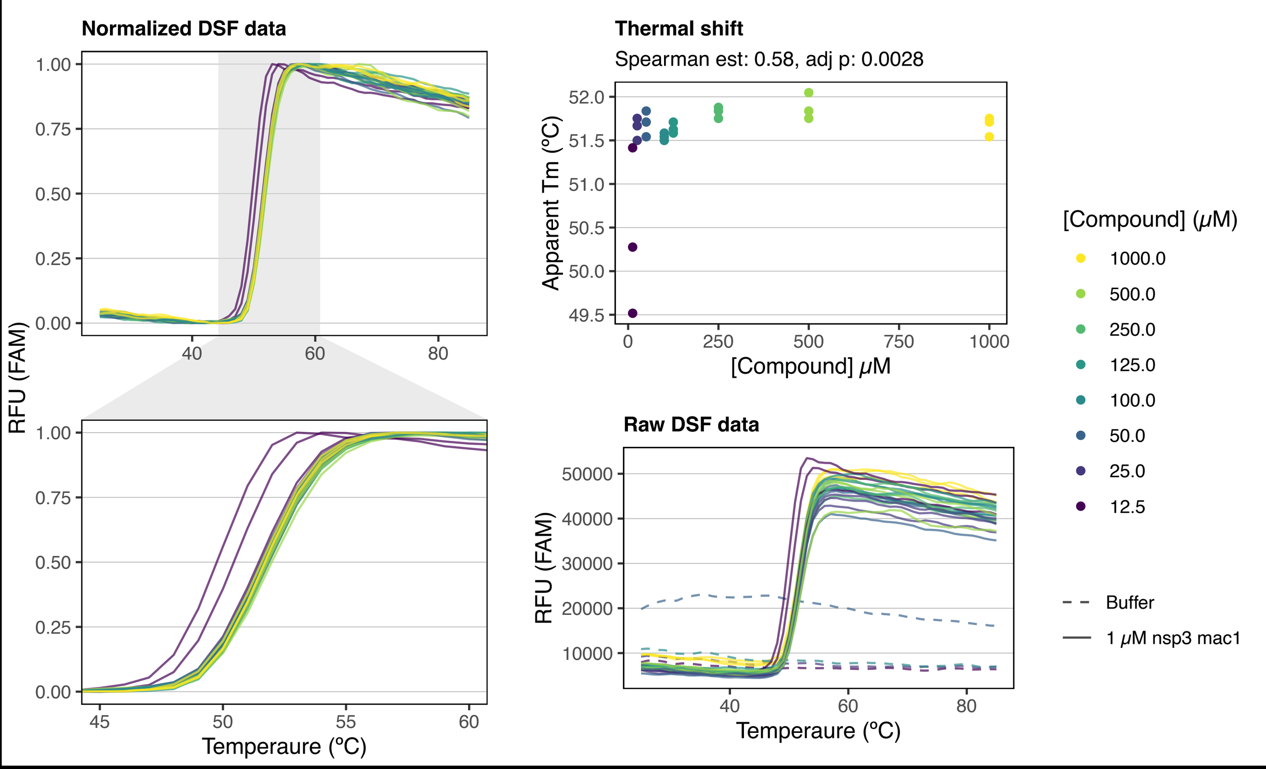

Compound: ZINC000827900828 (a.k.a ZINC000827900828) | Library: Make−on−demand

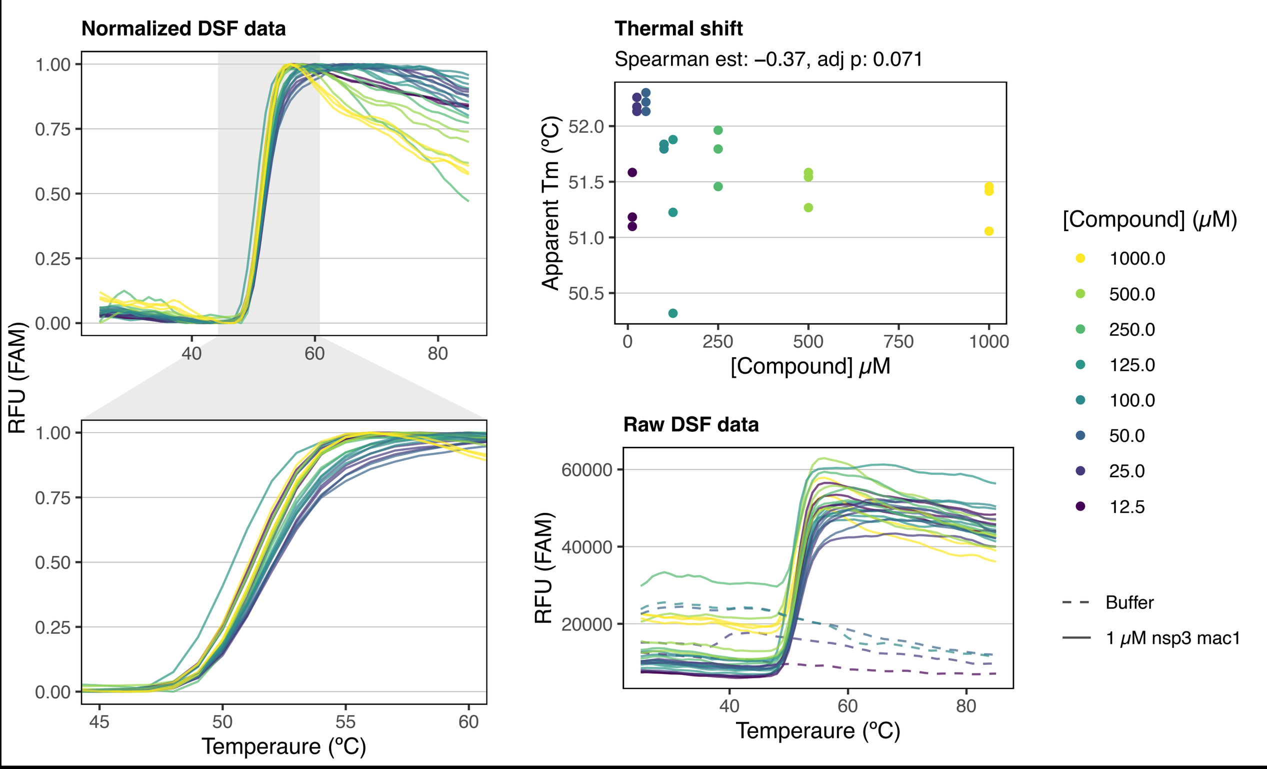

Compound: ZINC000835985505 (a.k.a ZINC000835985505) | Library: Make−on−demand

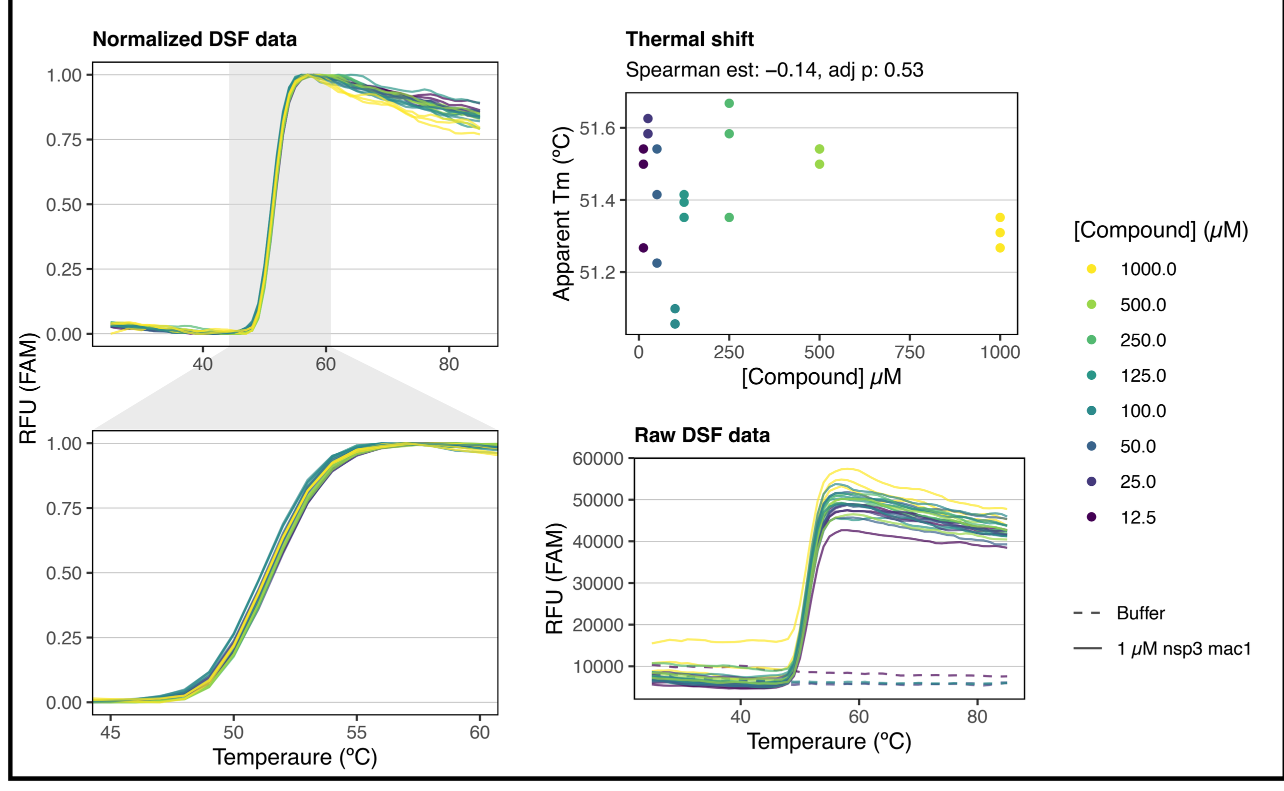

Compound: ZINC000865679550 (a.k.a ZINC000865679550) | Library: Make−on−demand

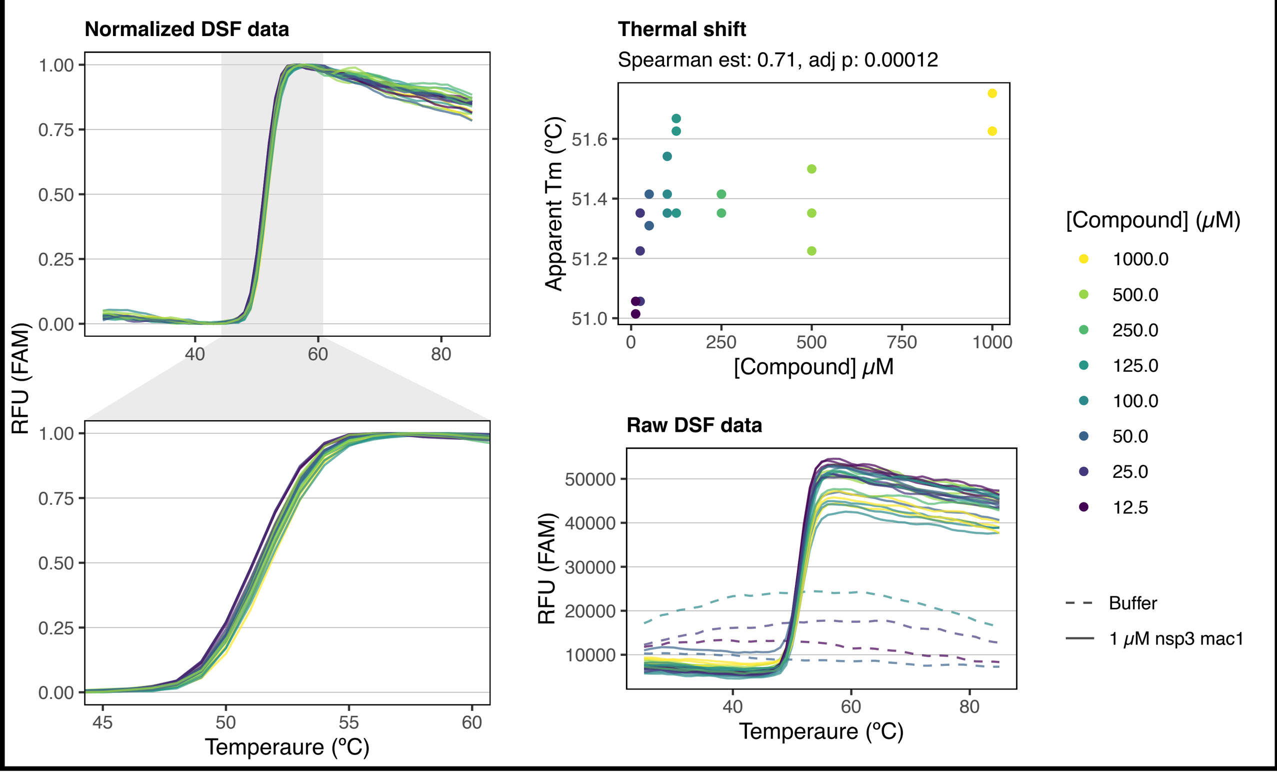

Compound: ZINC000866018680 (a.k.a ZINC000866018680) | Library: Make−on−demand

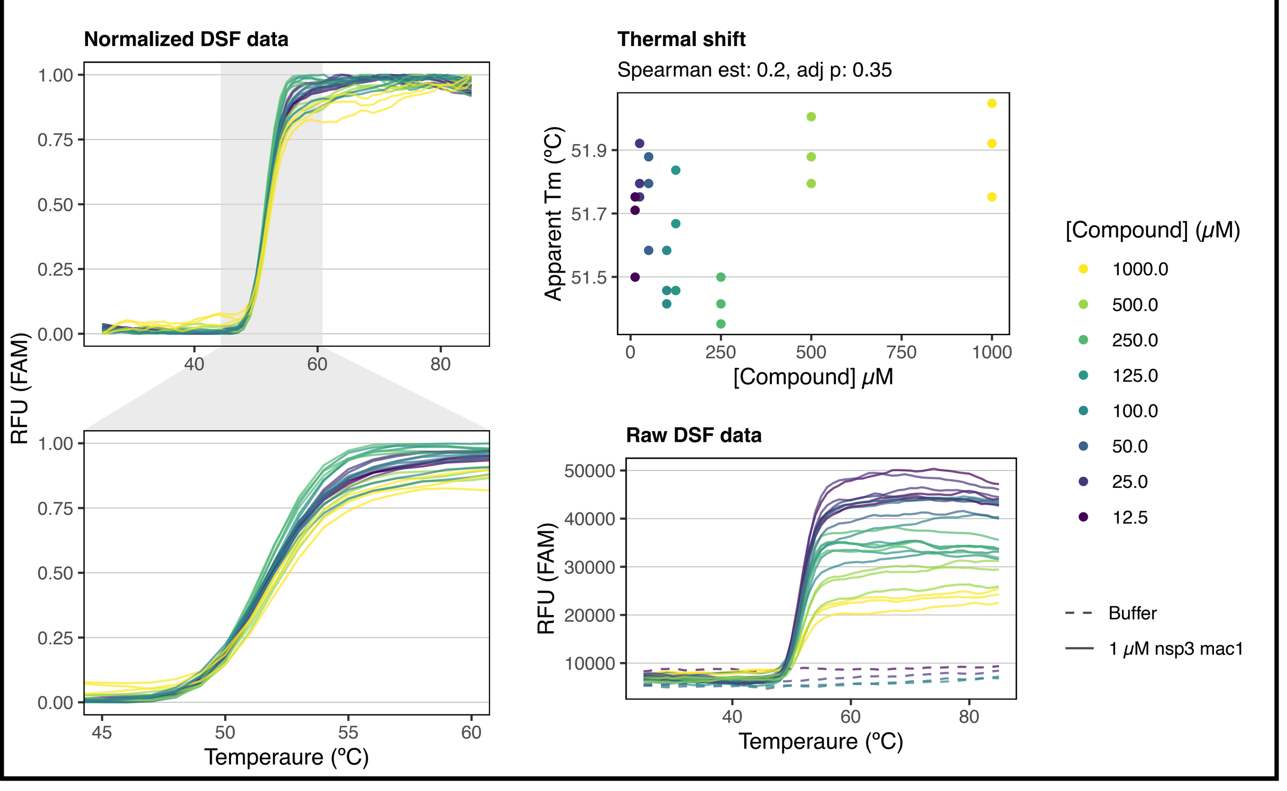

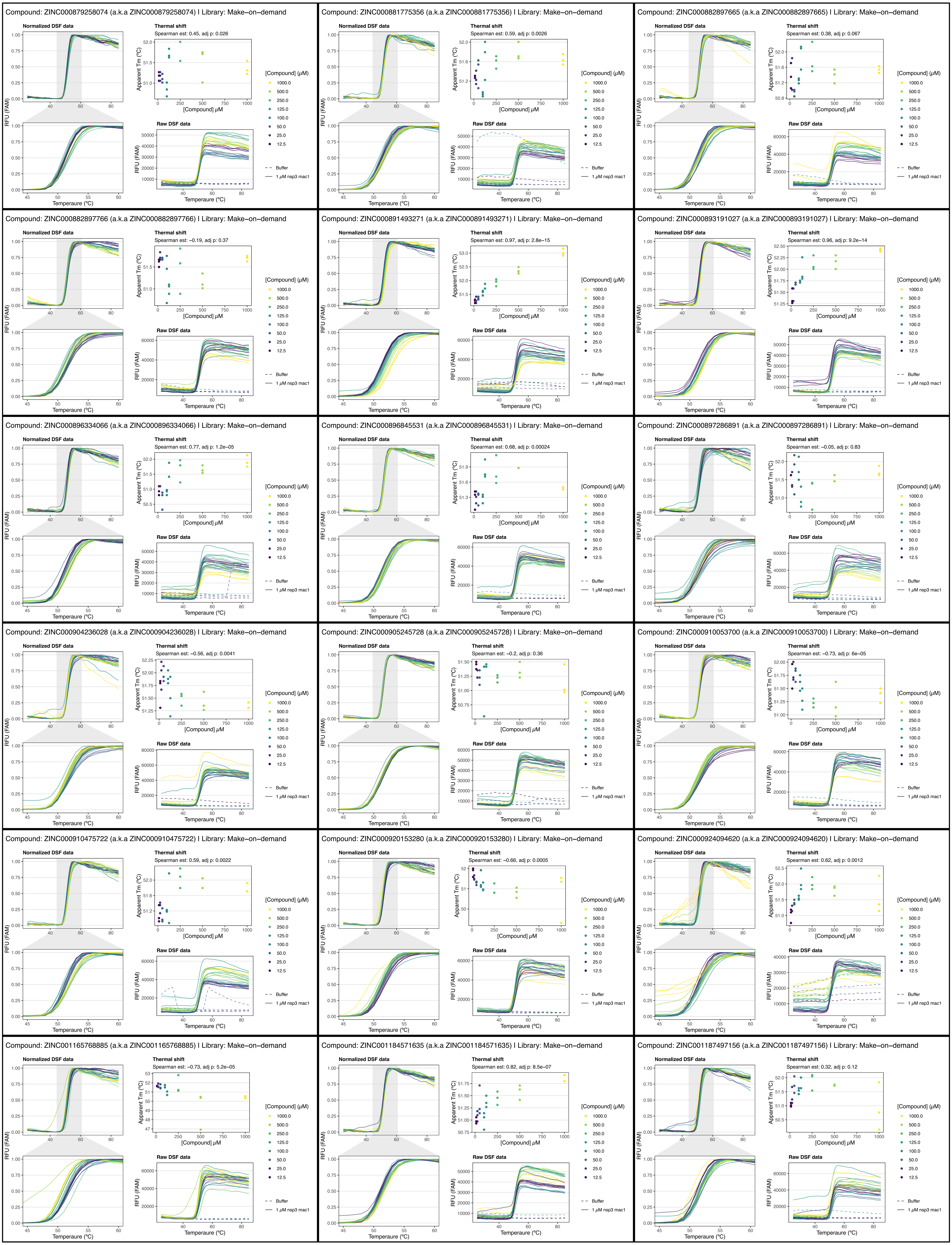
Compound: ZINC000879258074 (a.k.a ZINC000879258074) | Library: Make−on−demand
Normalized DSF data
Thermal shift
Spearman est: 0.45, adj p: 0.026
Raw DSF data
Compound: ZINC000881775356 (a.k.a ZINC000881775356) | Library: Make−on−demand
Spearman est: 0.59, adj p: 0.0026
Compound: ZINC000882897665 (a.k.a ZINC000882897665) | Library: Make−on−demand
Spearman est: 0.38, adj p: 0.067
Compound: ZINC000882897766 (a.k.a ZINC000882897766) | Library: Make−on−demand
Spearman est: −0.19, adj p: 0.37
Compound: ZINC000891493271 (a.k.a ZINC000891493271) | Library: Make−on−demand
Spearman est: 0.97, adj p: 2.8e−15
Compound: ZINC000893191027 (a.k.a ZINC000893191027) | Library: Make−on−demand
Spearman est: 0.96, adj p: 9.2e−14
Compound: ZINC000896334066 (a.k.a ZINC000896334066) | Library: Make−on−demand
Spearman est: 0.77, adj p: 1.2e−05
Compound: ZINC000896845531 (a.k.a ZINC000896845531) | Library: Make−on−demand
Spearman est: 0.68, adj p: 0.00024
Compound: ZINC000897286891 (a.k.a ZINC000897286891) | Library: Make−on−demand
Spearman est: −0.05, adj p: 0.83
Compound: ZINC000904236028 (a.k.a ZINC000904236028) | Library: Make−on−demand
Spearman est: −0.56, adj p: 0.0041
Compound: ZINC000905245728 (a.k.a ZINC000905245728) | Library: Make−on−demand
Spearman est: −0.2, adj p: 0.36
Compound: ZINC000910053700 (a.k.a ZINC000910053700) | Library: Make−on−demand
Spearman est: −0.73, adj p: 6e−05
Compound: ZINC000910475722 (a.k.a ZINC000910475722) | Library: Make−on−demand
Spearman est: 0.59, adj p: 0.0022
Compound: ZINC000920153280 (a.k.a ZINC000920153280) | Library: Make−on−demand
Spearman est: −0.66, adj p: 0.0005
Compound: ZINC000924094620 (a.k.a ZINC000924094620) | Library: Make−on−demand
Spearman est: 0.62, adj p: 0.0012
Compound: ZINC001165768885 (a.k.a ZINC001165768885) | Library: Make−on−demand
Spearman est: −0.73, adj p: 5.2e−05
Compound: ZINC001184571635 (a.k.a ZINC001184571635) | Library: Make−on−demand
Spearman est: 0.82, adj p: 8.5e−07
Compound: ZINC001187497156 (a.k.a ZINC001187497156) | Library: Make−on−demand
Spearman est: 0.32, adj p: 0.12
[Compound] (μM)
Buffer
1 μM nsp3 mac1
Temperature (°C)
RFU (FAM)
Apparent Tm (°C)

Compound: ZINC001337833291 (a.k.a ZINC001337833291) | Library: Make−on−demand

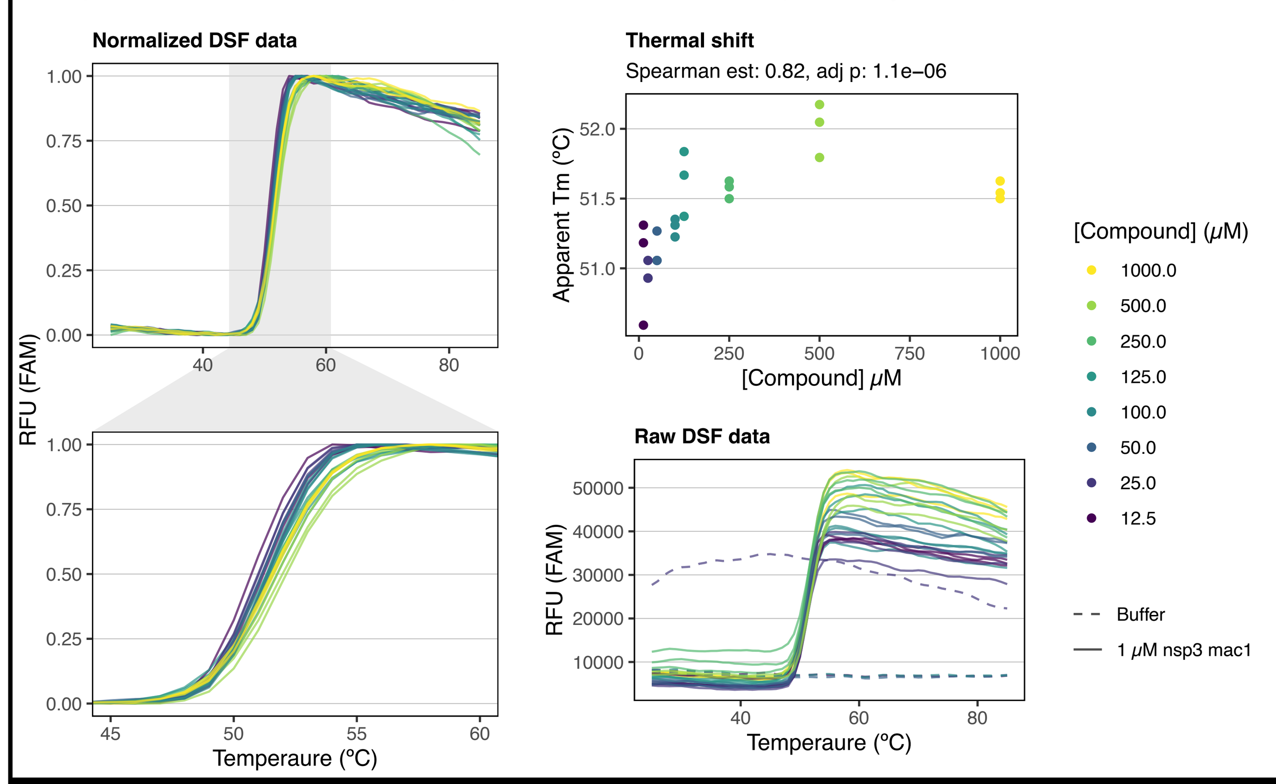

Compound: ZINC001364194305 (a.k.a ZINC001364194305) | Library: Make−on−demand

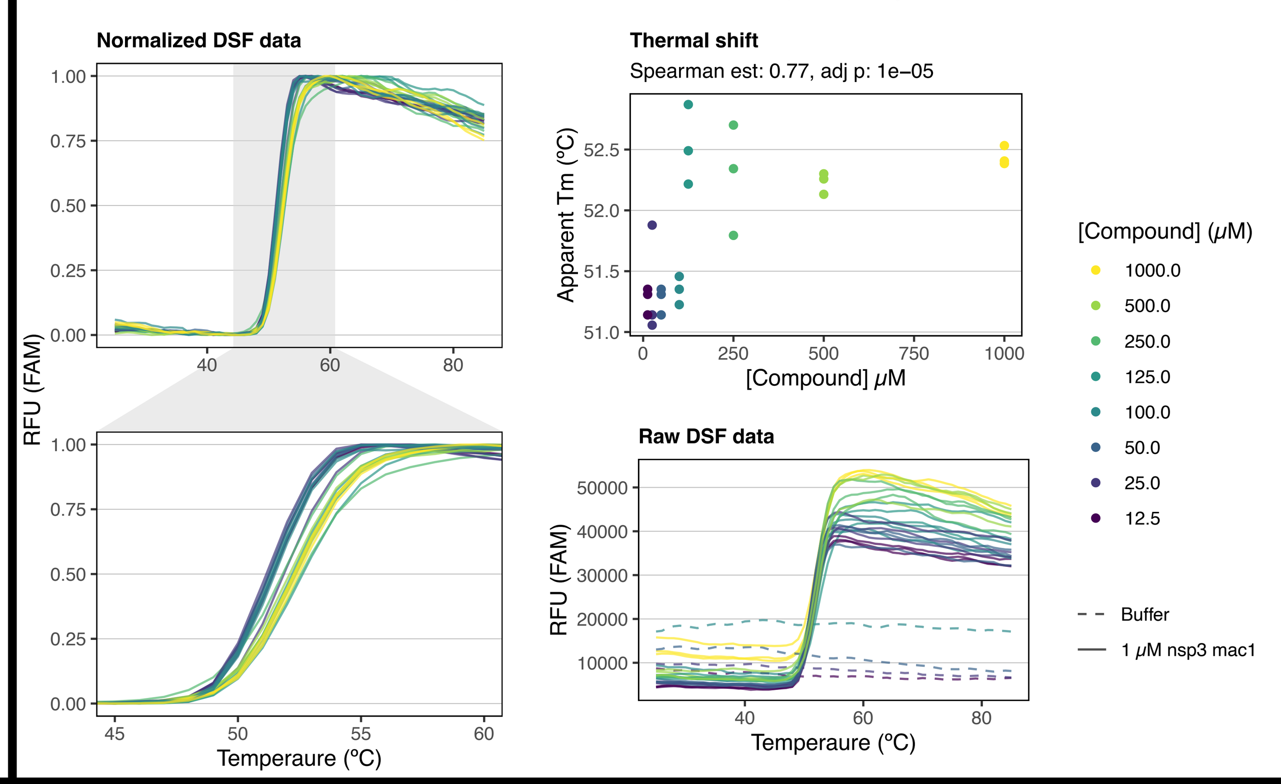

Compound: ZINC001364630288 (a.k.a ZINC001364630288) | Library: Make−on−demand

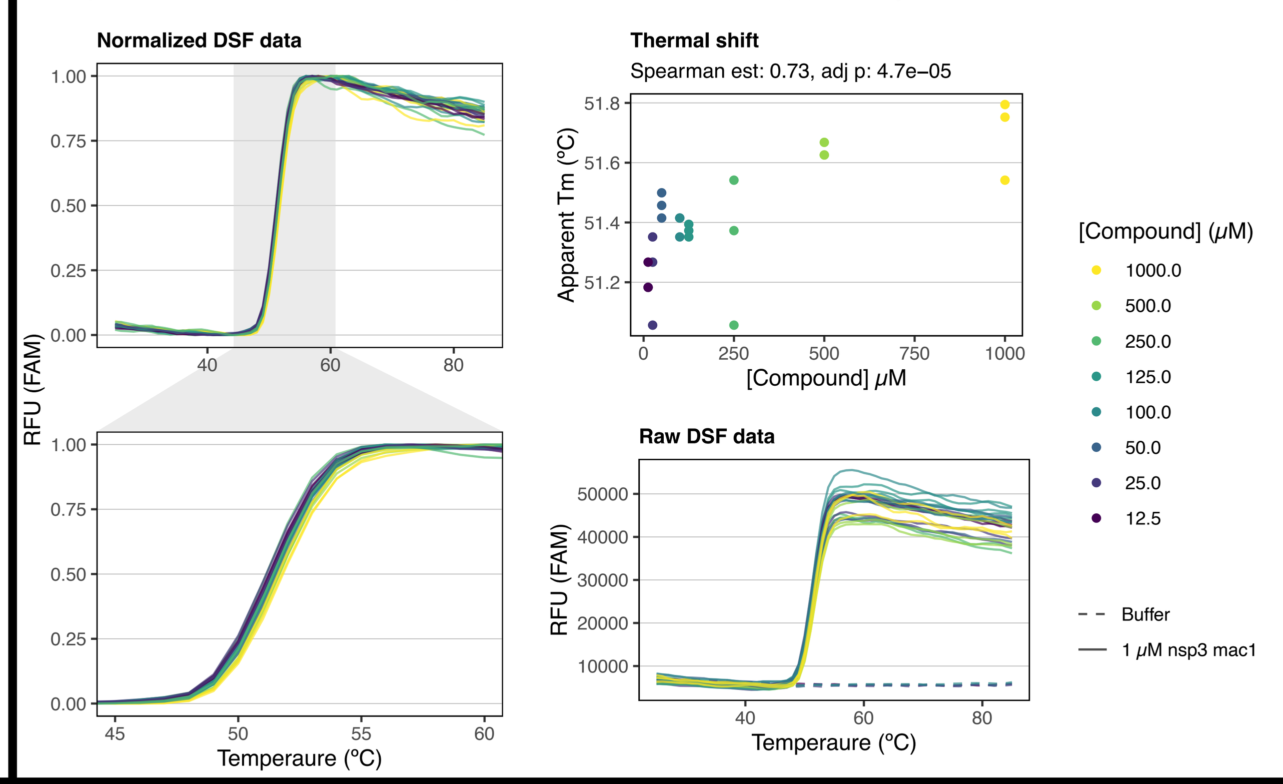

Compound: ZINC001458791015 (a.k.a ZINC001458791015) | Library: Make−on−demand

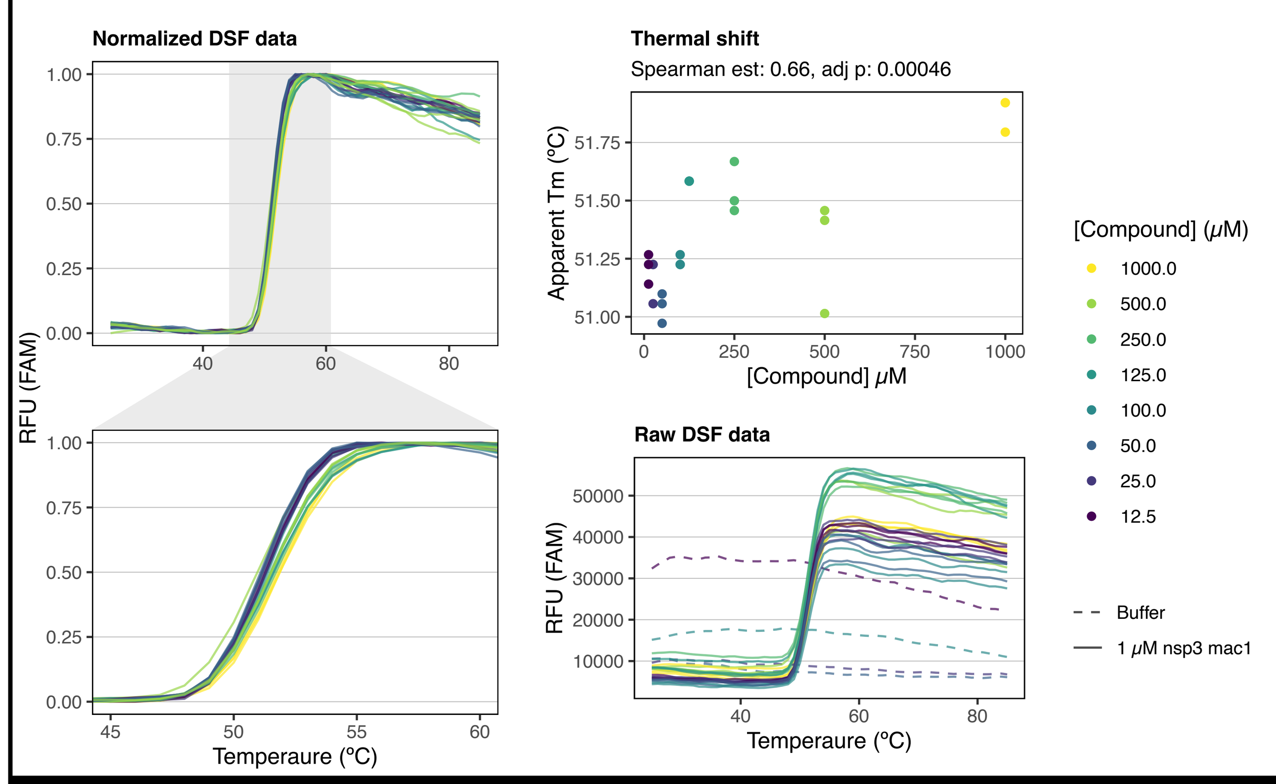

Compound: ZINC001472868186 (a.k.a ZINC001472868186) | Library: Make−on−demand

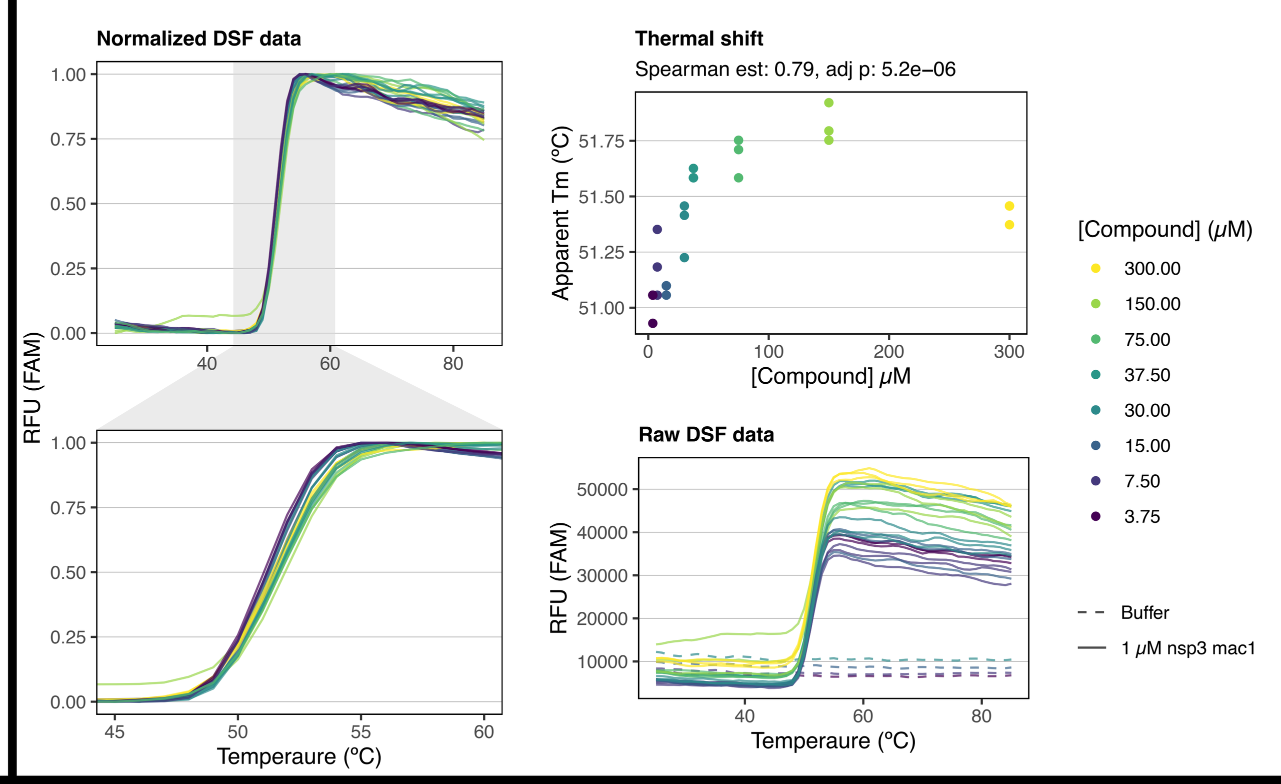

Compound: ZINC001559178387 (a.k.a ZINC001559178387) | Library: Make−on−demand

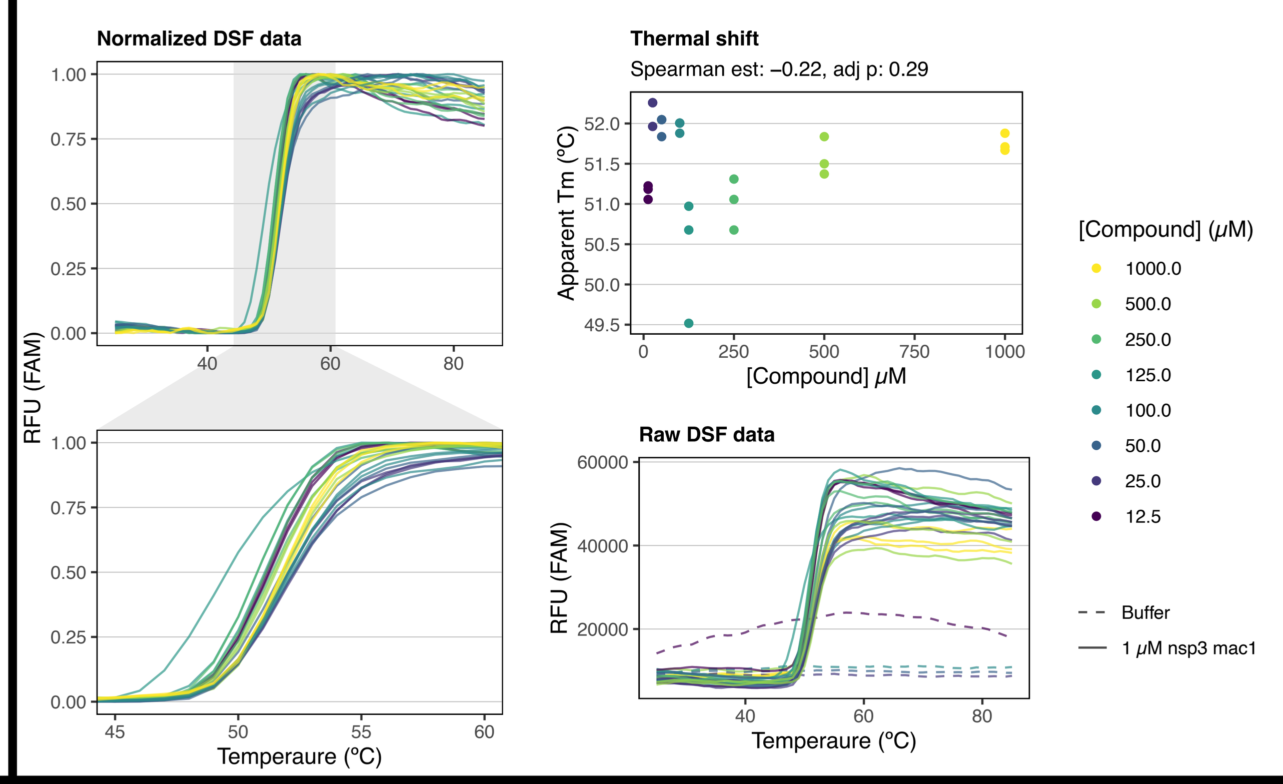

Compound: ZINC001641481086 (a.k.a ZINC001641481086) | Library: Make−on−demand

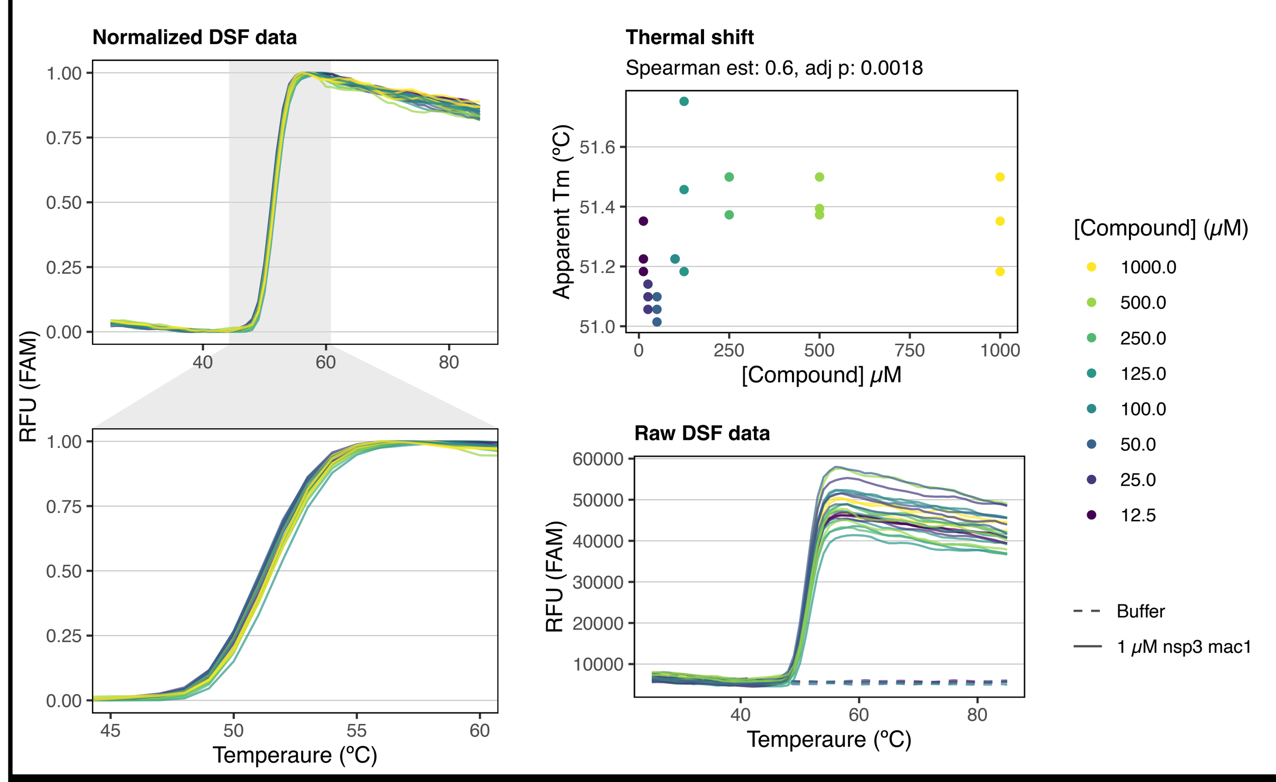

Compound: ZINC001652467570 (a.k.a ZINC001652467570) | Library: Make−on−demand

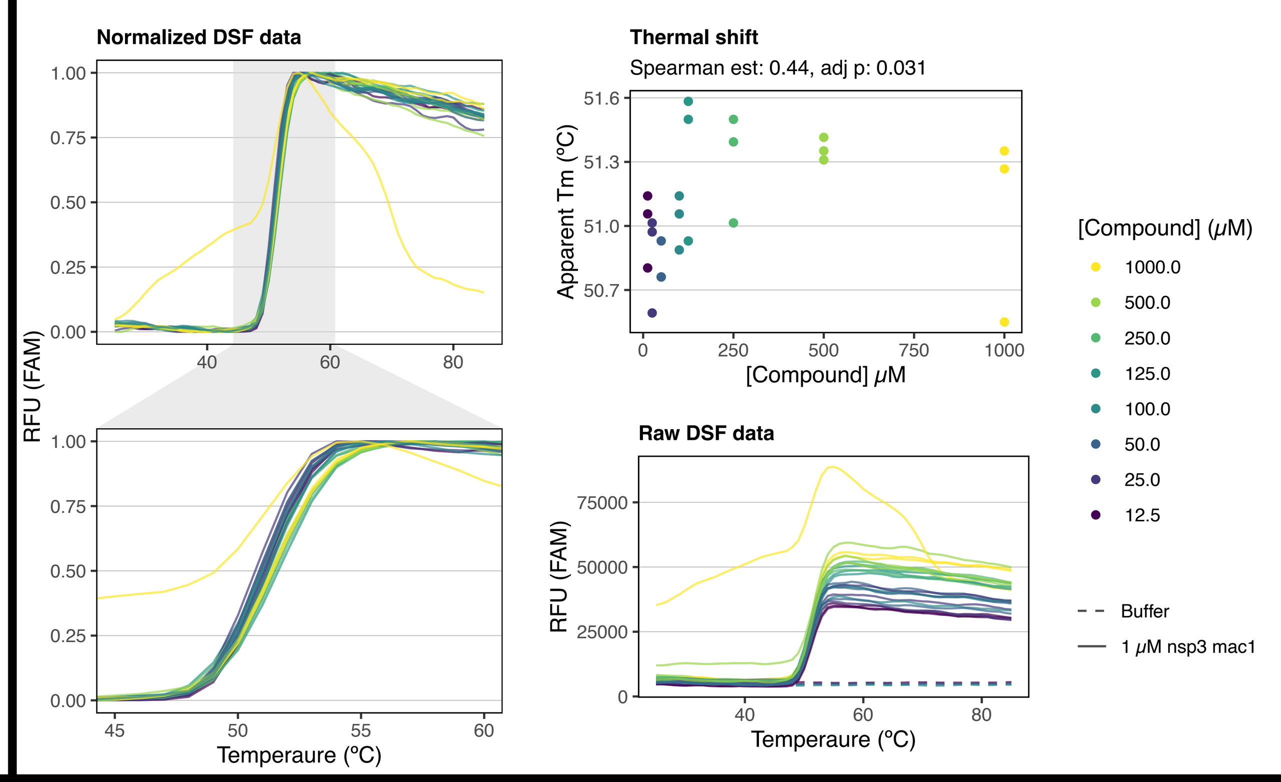

Compound: Z1082933628 (a.k.a Z1082933628) | Library: Merger−order−1

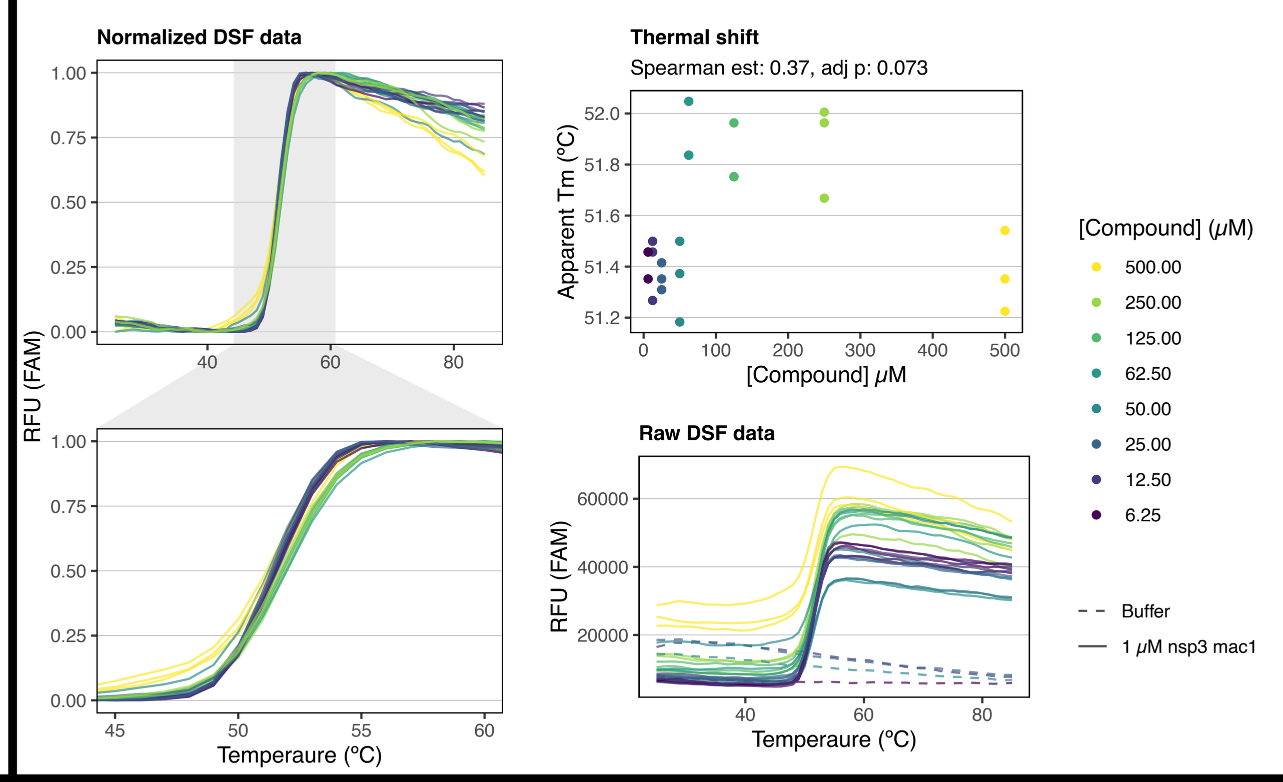

Compound: Z4574659604 (a.k.a Z4574659604) | Library: Merger−order−1

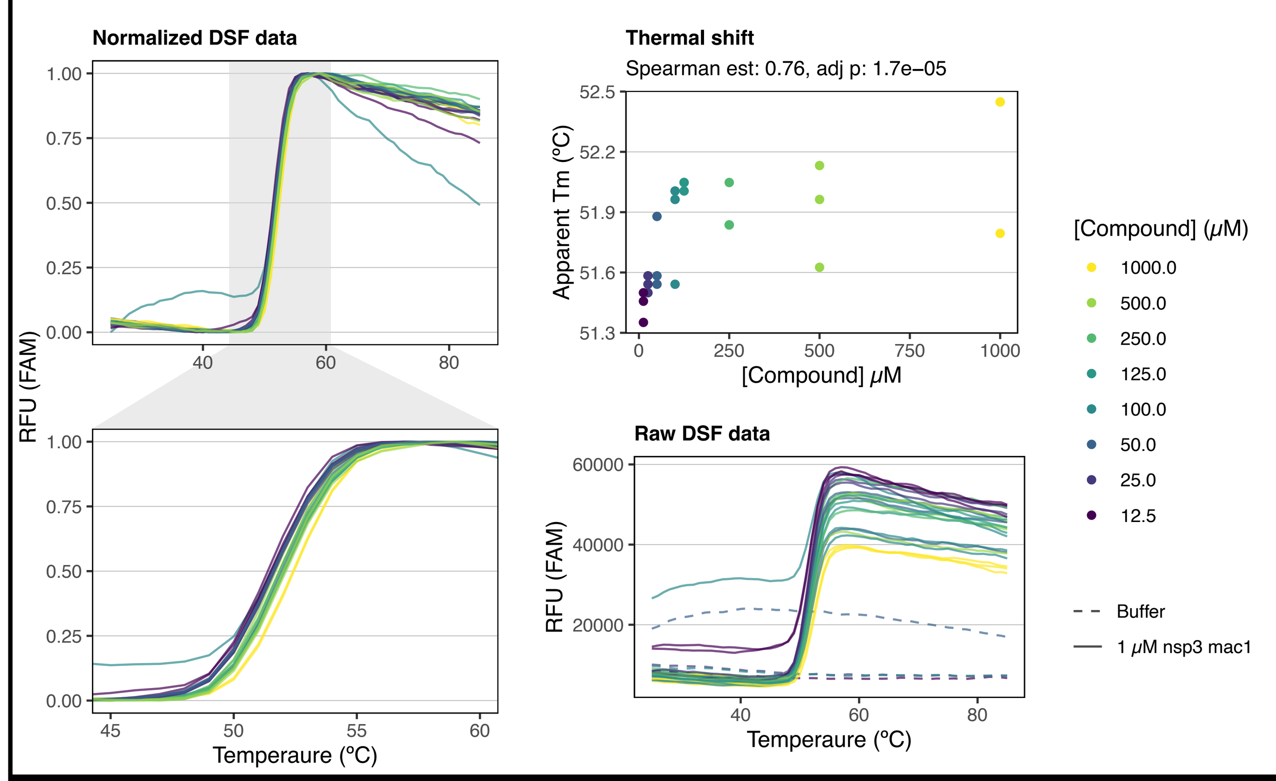

Compound: Z4718398515 (a.k.a Z4718398515) | Library: Merger−order−1

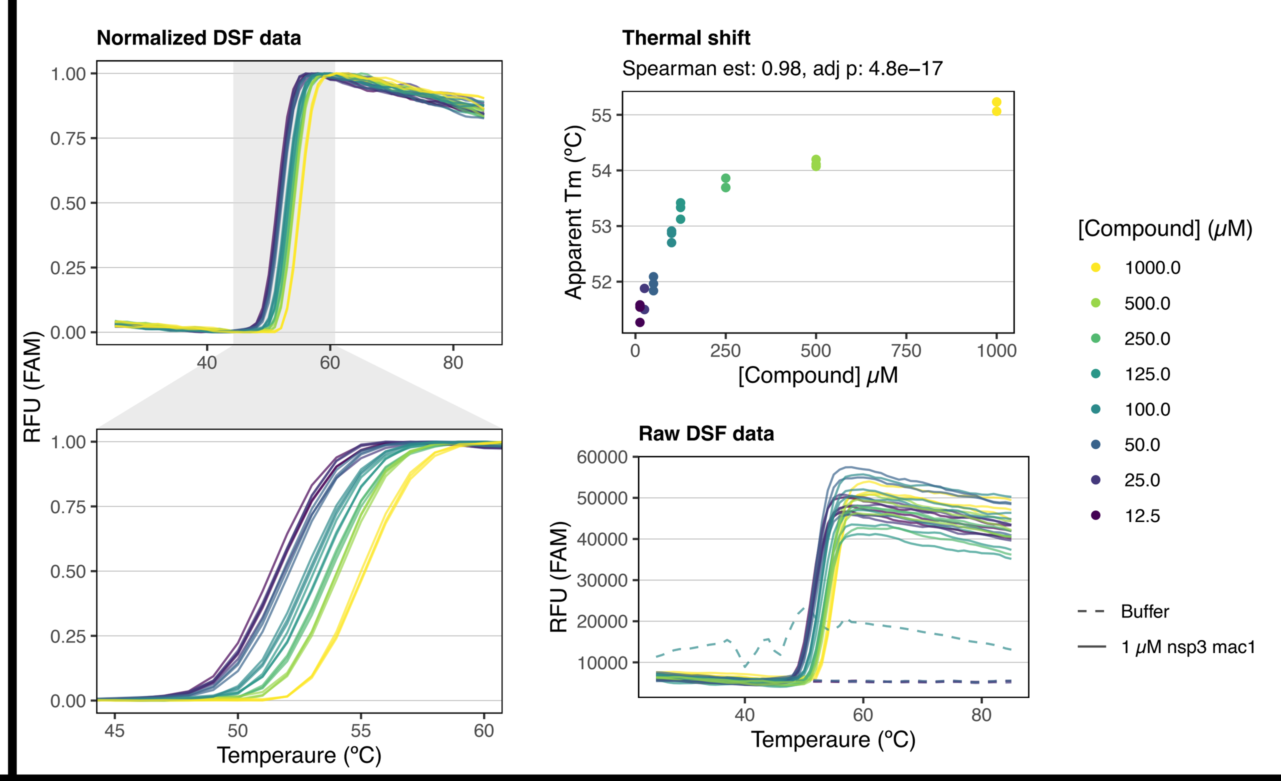

Compound: Z4718398531 (a.k.a Z4718398531) | Library: Merger−order−1

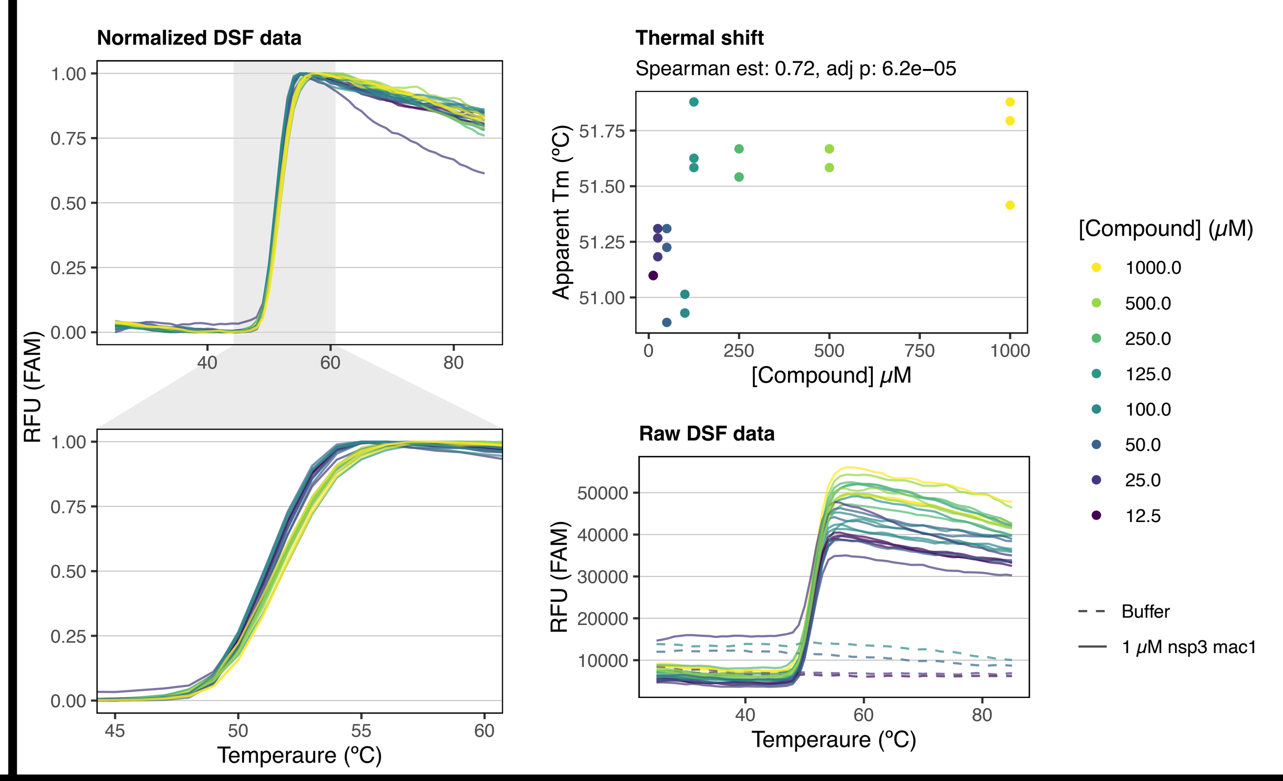

Compound: Z4718398539 (a.k.a Z4718398539) | Library: Merger−order−1

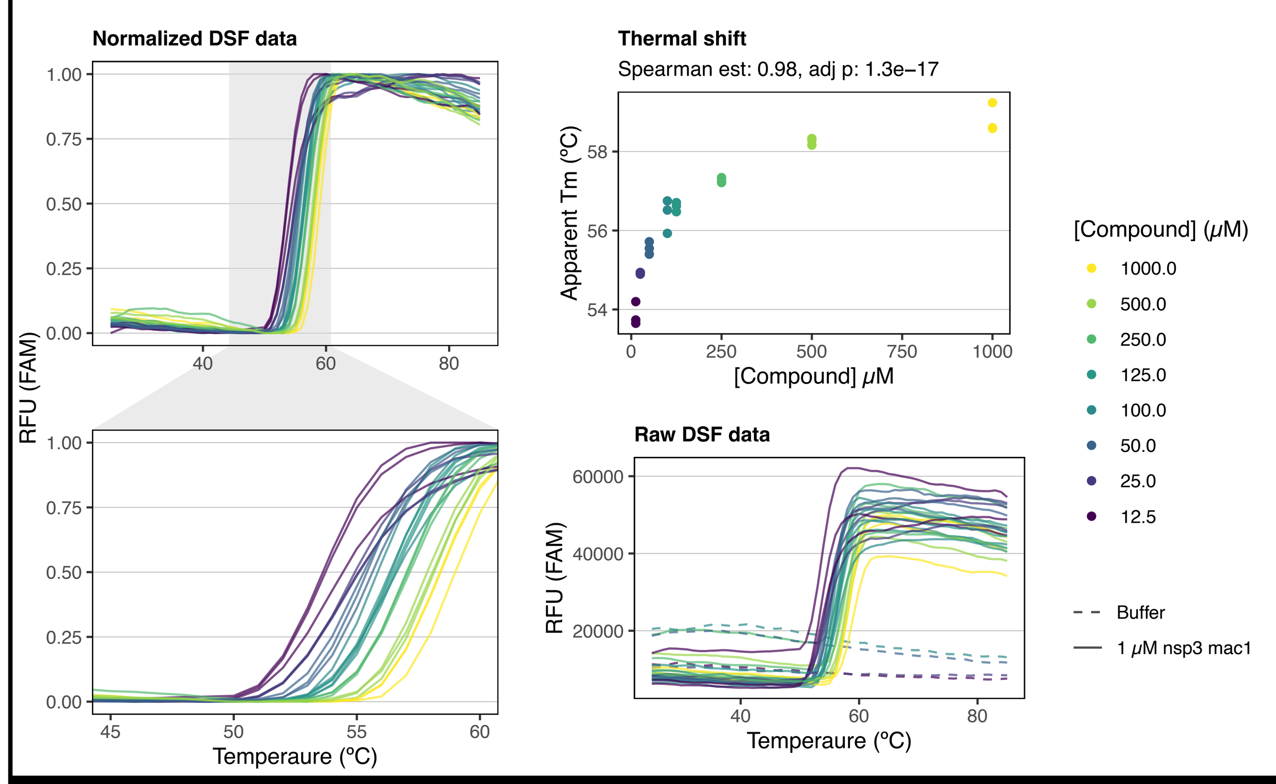

Compound: Z4718398554 (a.k.a Z4718398554) | Library: Merger−order−1

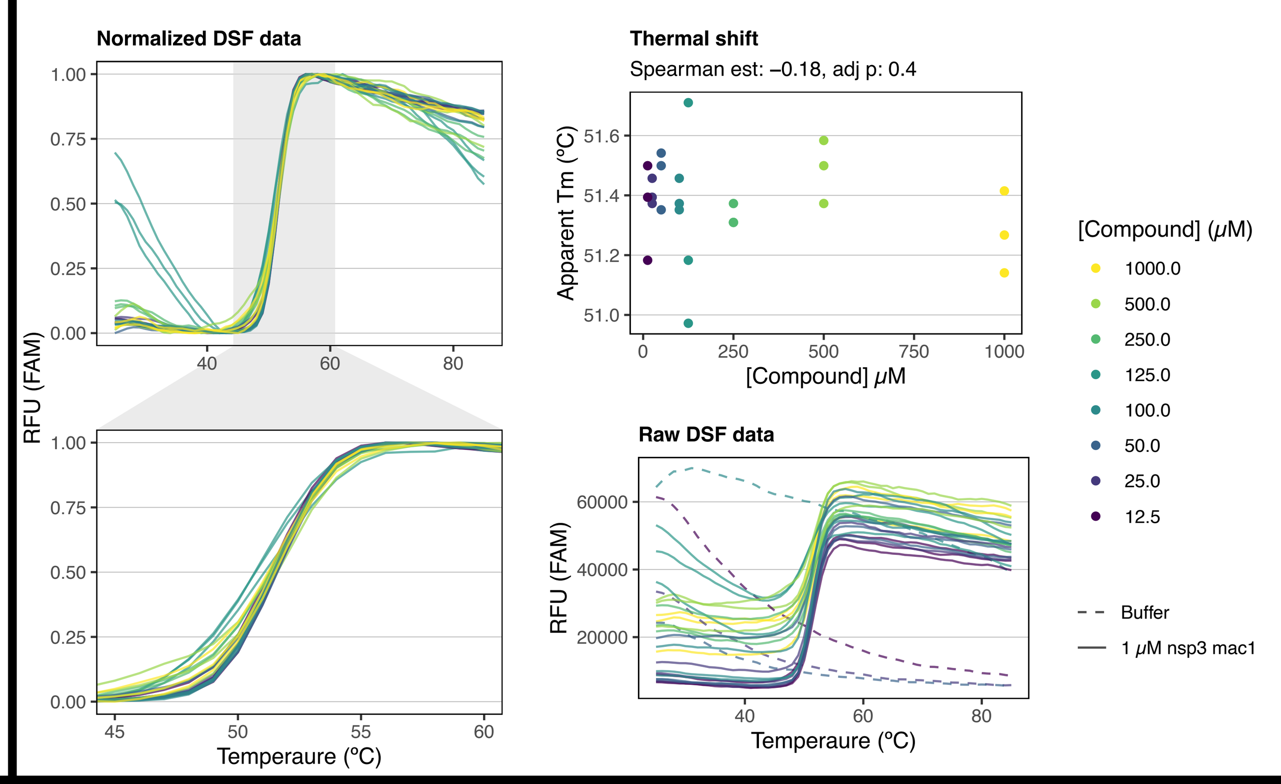

Compound: Z4718398569 (a.k.a Z4718398569) | Library: Merger−order−1

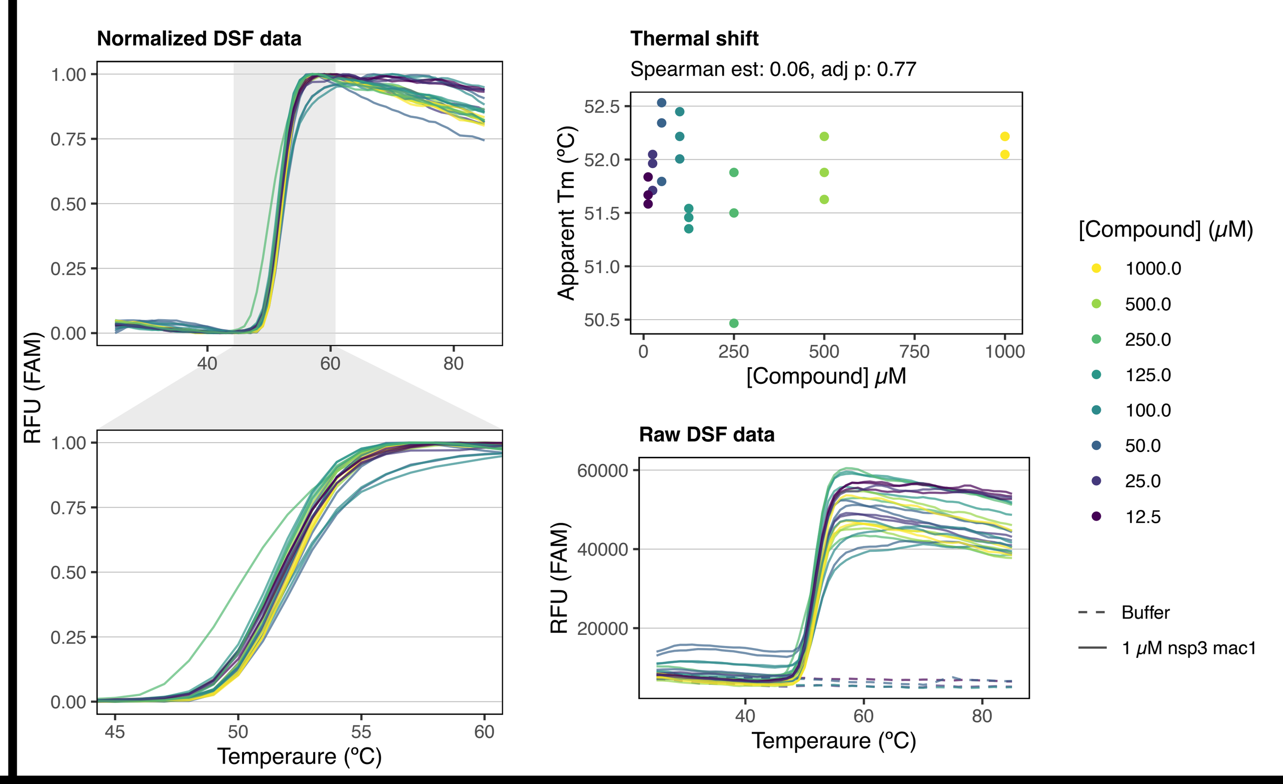

Compound: Z4718398572 (a.k.a Z4718398572) | Library: Merger−order−1

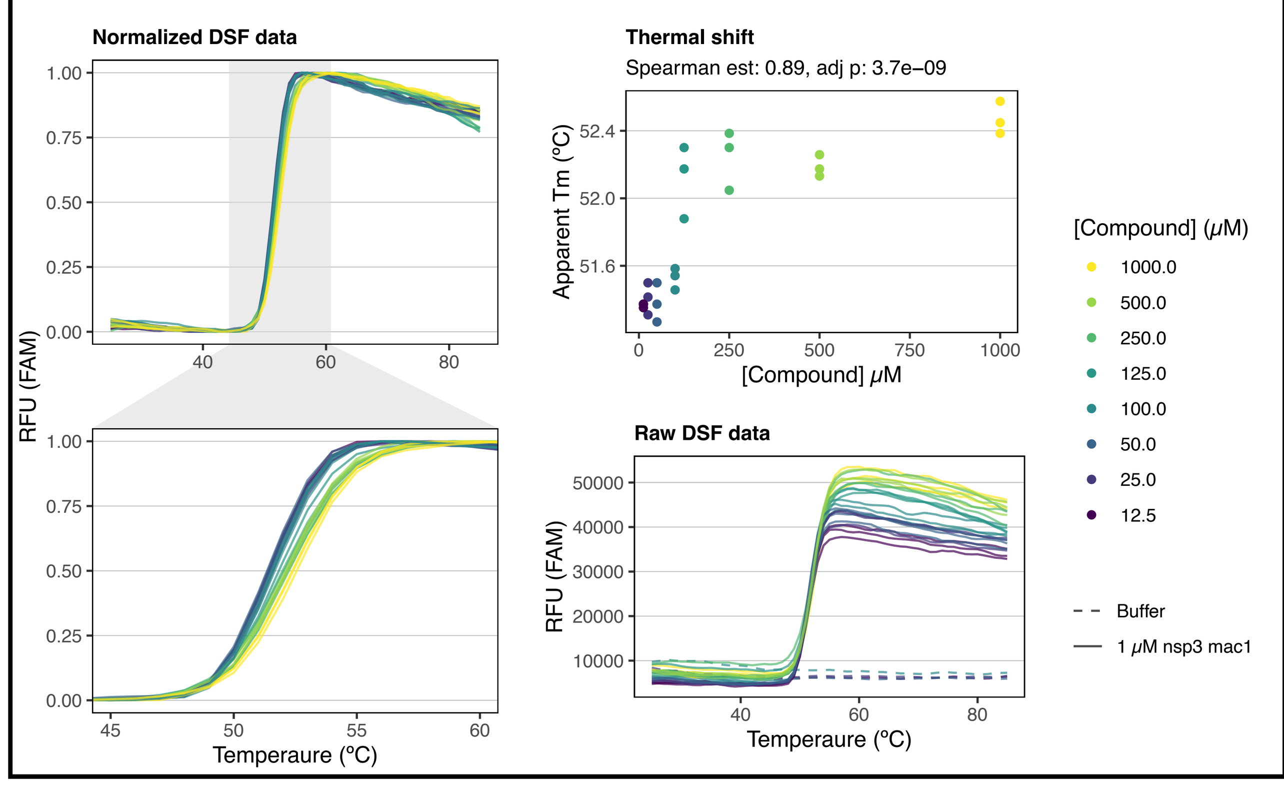

Compound: Z4718398580 (a.k.a Z4718398580) | Library: Merger−order−1

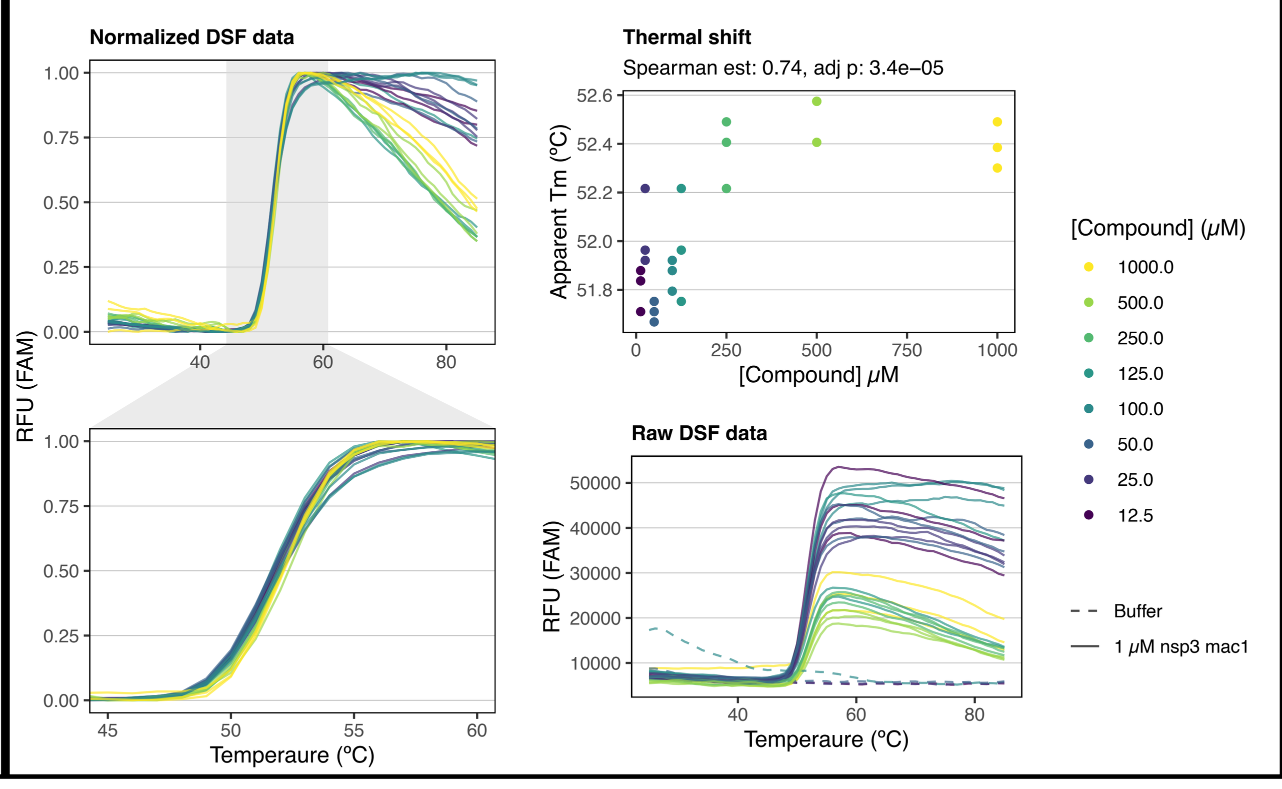

Compound: Z4718398585 (a.k.a Z4718398585) | Library: Merger−order−1

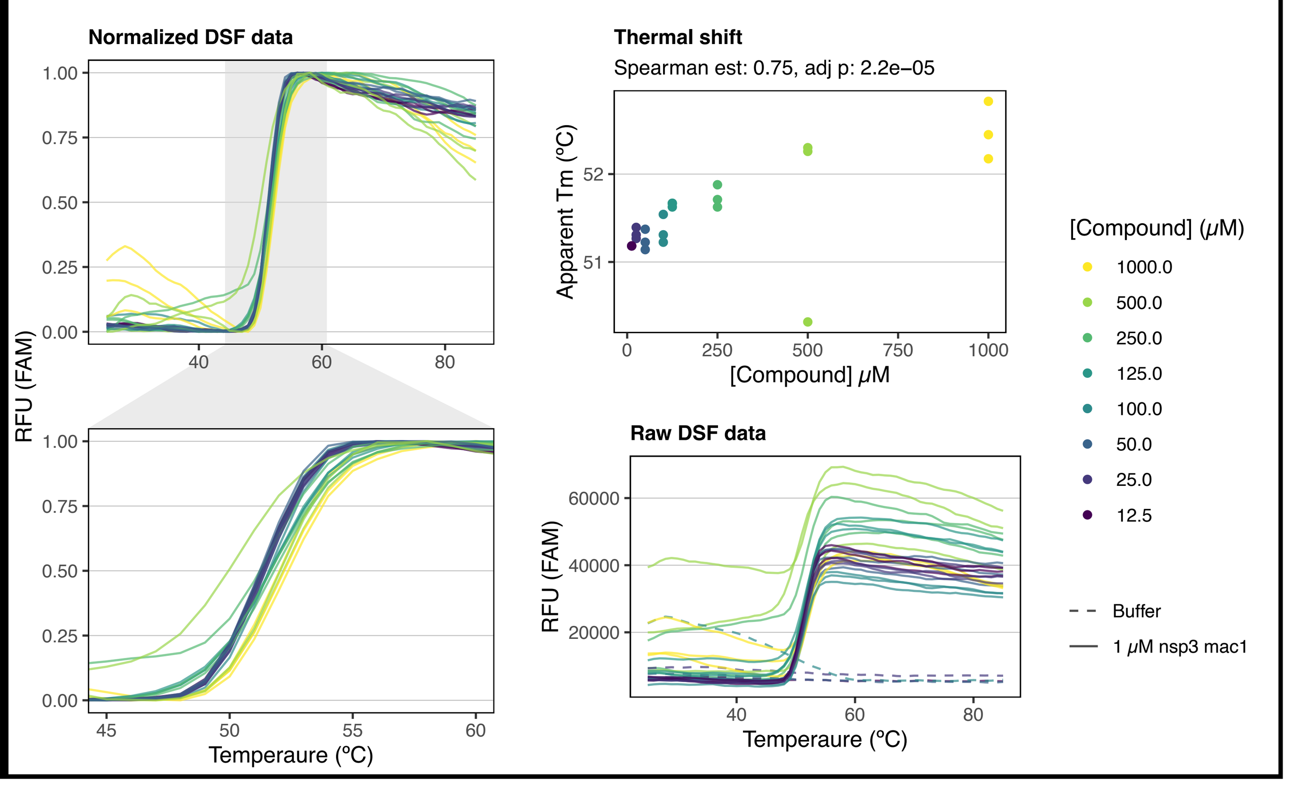

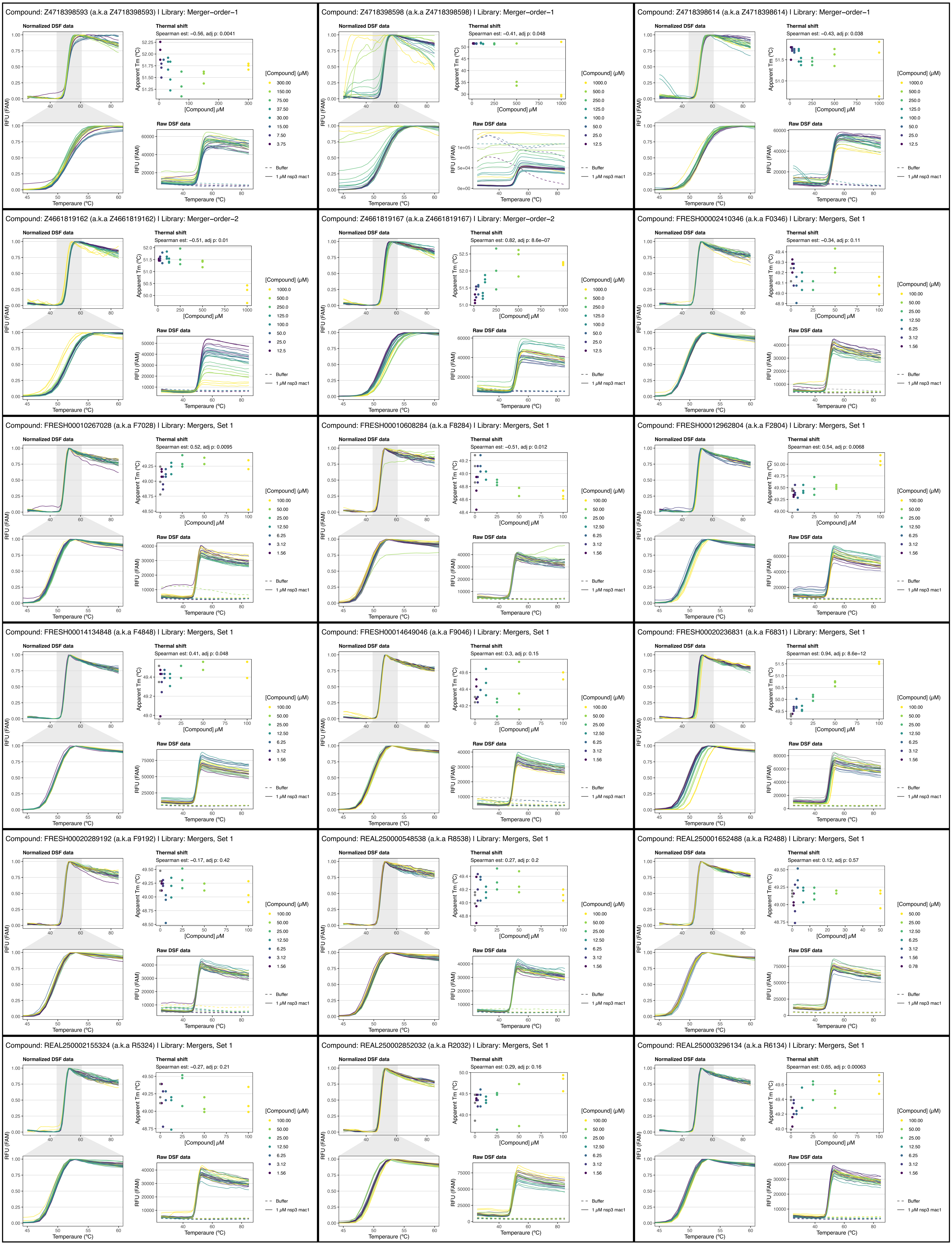
Compound: Z4718398593 (a.k.a Z4718398593) I Library: Merger–order–1
Normalized DSF data
Thermal shift
Spearman est: −0.56, adj p: 0.0041
Raw DSF data
Compound: Z4718398598 (a.k.a Z4718398598) I Library: Merger–order–1
Spearman est: −0.41, adj p: 0.048
Compound: Z4718398614 (a.k.a Z4718398614) I Library: Merger–order–1
Spearman est: −0.43, adj p: 0.038
Compound: Z4661819162 (a.k.a Z4661819162) I Library: Merger–order–2
Spearman est: −0.51, adj p: 0.01
Compound: Z4661819167 (a.k.a Z4661819167) I Library: Merger–order–2
Spearman est: 0.82, adj p: 8.6e−07
Compound: FRESH00002410346 (a.k.a F0346) I Library: Mergers, Set 1
Spearman est: −0.34, adj p: 0.11
Compound: FRESH00010267028 (a.k.a F7028) I Library: Mergers, Set 1
Spearman est: 0.52, adj p: 0.0095
Compound: FRESH00010608284 (a.k.a F8284) I Library: Mergers, Set 1
Spearman est: −0.51, adj p: 0.012
Compound: FRESH00012962804 (a.k.a F2804) I Library: Mergers, Set 1
Spearman est: 0.54, adj p: 0.0068
Compound: FRESH00014134848 (a.k.a F4848) I Library: Mergers, Set 1
Spearman est: 0.41, adj p: 0.048
Compound: FRESH00014649046 (a.k.a F9046) I Library: Mergers, Set 1
Spearman est: 0.3, adj p: 0.15
Compound: FRESH00020236831 (a.k.a F6831) I Library: Mergers, Set 1
Spearman est: 0.94, adj p: 8.6e−12
Compound: FRESH00020289192 (a.k.a F9192) I Library: Mergers, Set 1
Spearman est: −0.17, adj p: 0.42
Compound: REAL250000548538 (a.k.a R8538) I Library: Mergers, Set 1
Spearman est: 0.27, adj p: 0.2
Compound: REAL250001652488 (a.k.a R2488) I Library: Mergers, Set 1
Spearman est: 0.12, adj p: 0.57
Compound: REAL250002155324 (a.k.a R5324) I Library: Mergers, Set 1
Spearman est: −0.27, adj p: 0.21
Compound: REAL250002852032 (a.k.a R2032) I Library: Mergers, Set 1
Spearman est: 0.29, adj p: 0.16
Compound: REAL250003296134 (a.k.a R6134) I Library: Mergers, Set 1
Spearman est: 0.65, adj p: 0.00063
Apparent Tm (°C)
[Compound] μM
RFU (FAM)
Temperaure (°C)
Buffer
1 μM nsp3 mac1

Compound: REAL250003324764 (a.k.a R4764) | Library: Mergers, Set 1

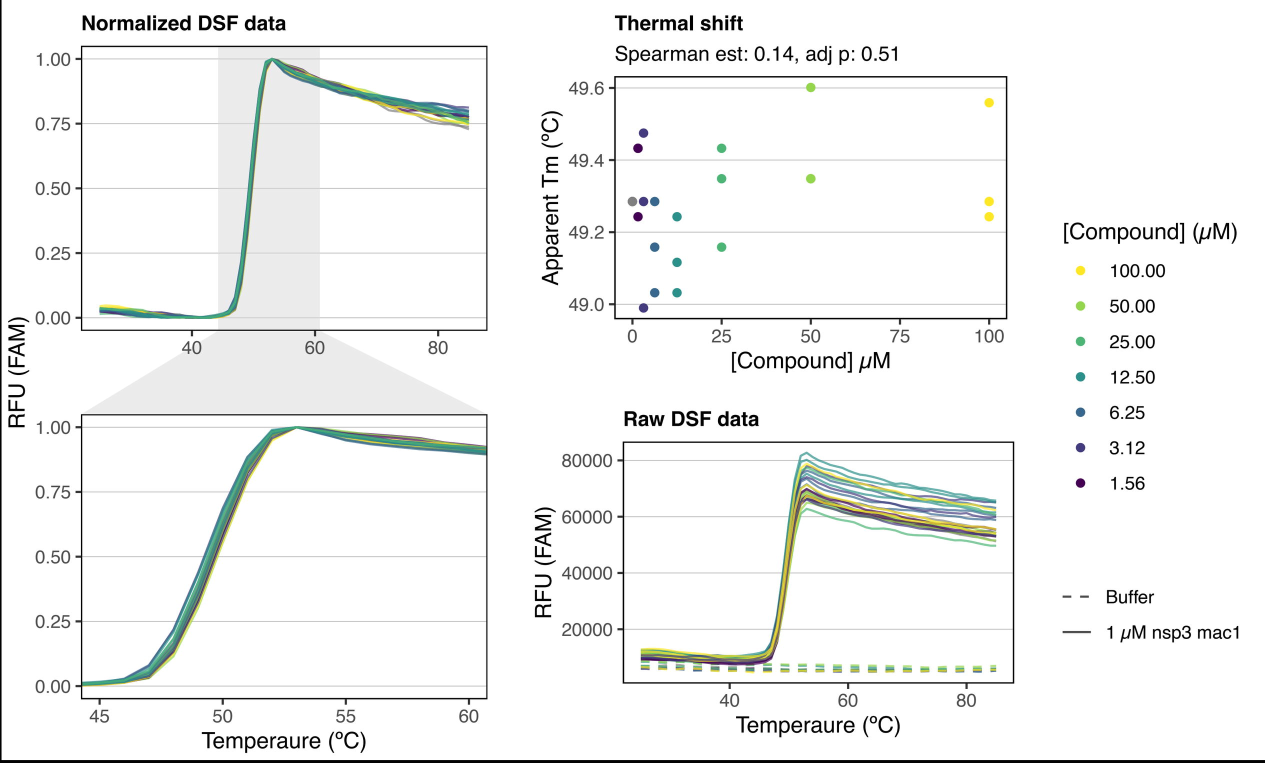

Compound: REAL250004627335 (a.k.a R7335) | Library: Mergers, Set 1

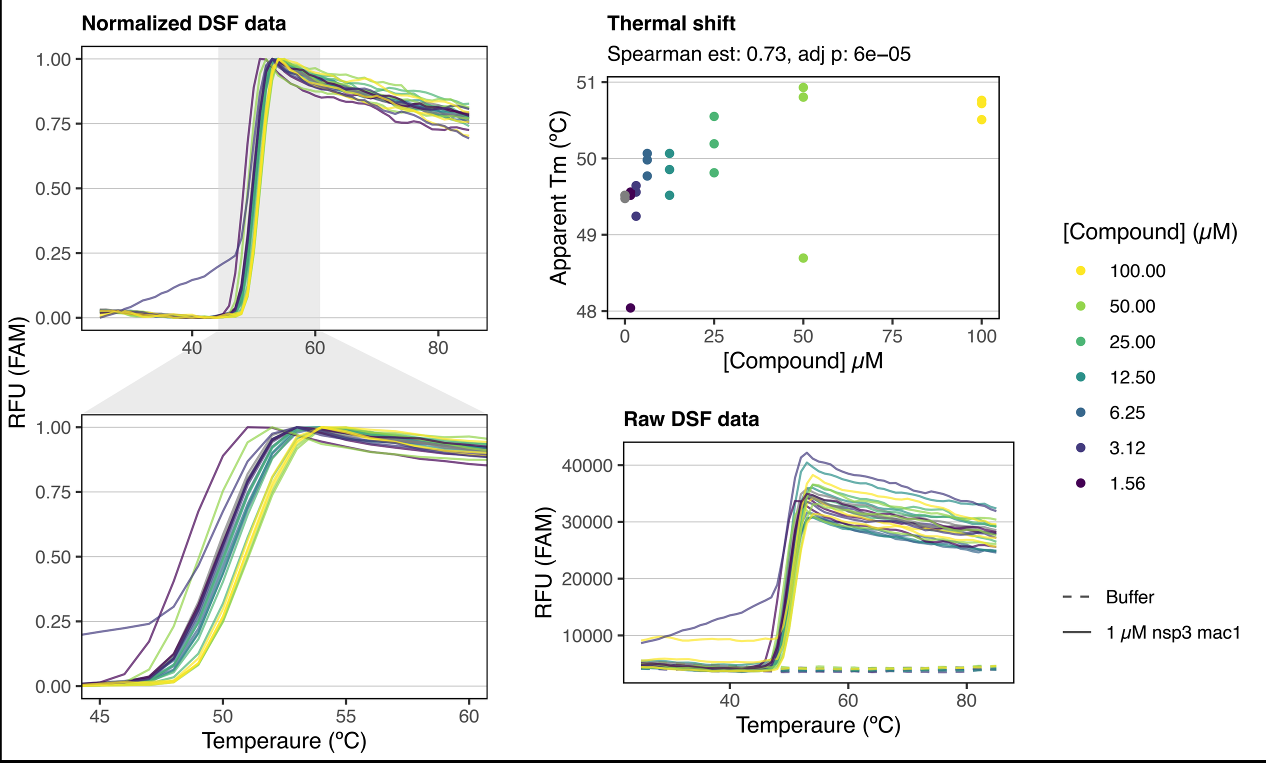

Compound: REAL300007318908 (a.k.a R8908) | Library: Mergers, Set 1

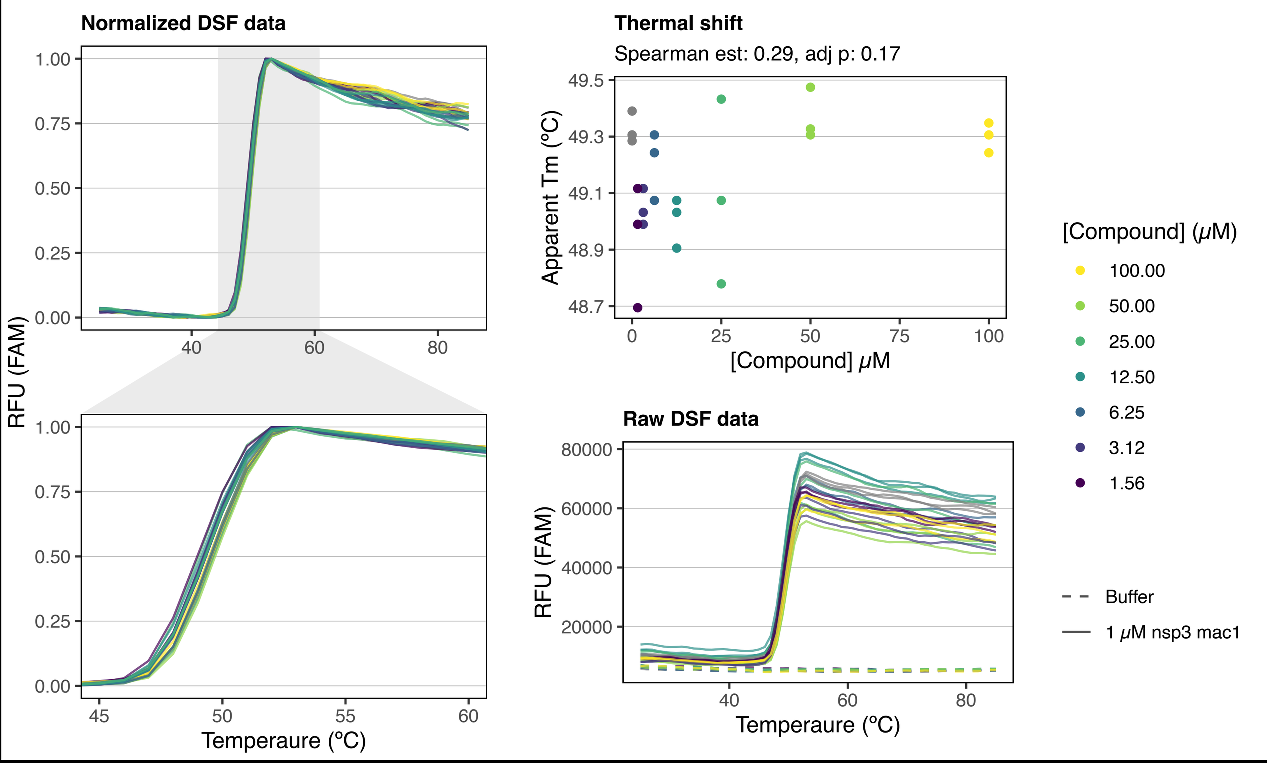

Compound: REAL300016493575 (a.k.a R3575) | Library: Mergers, Set 1

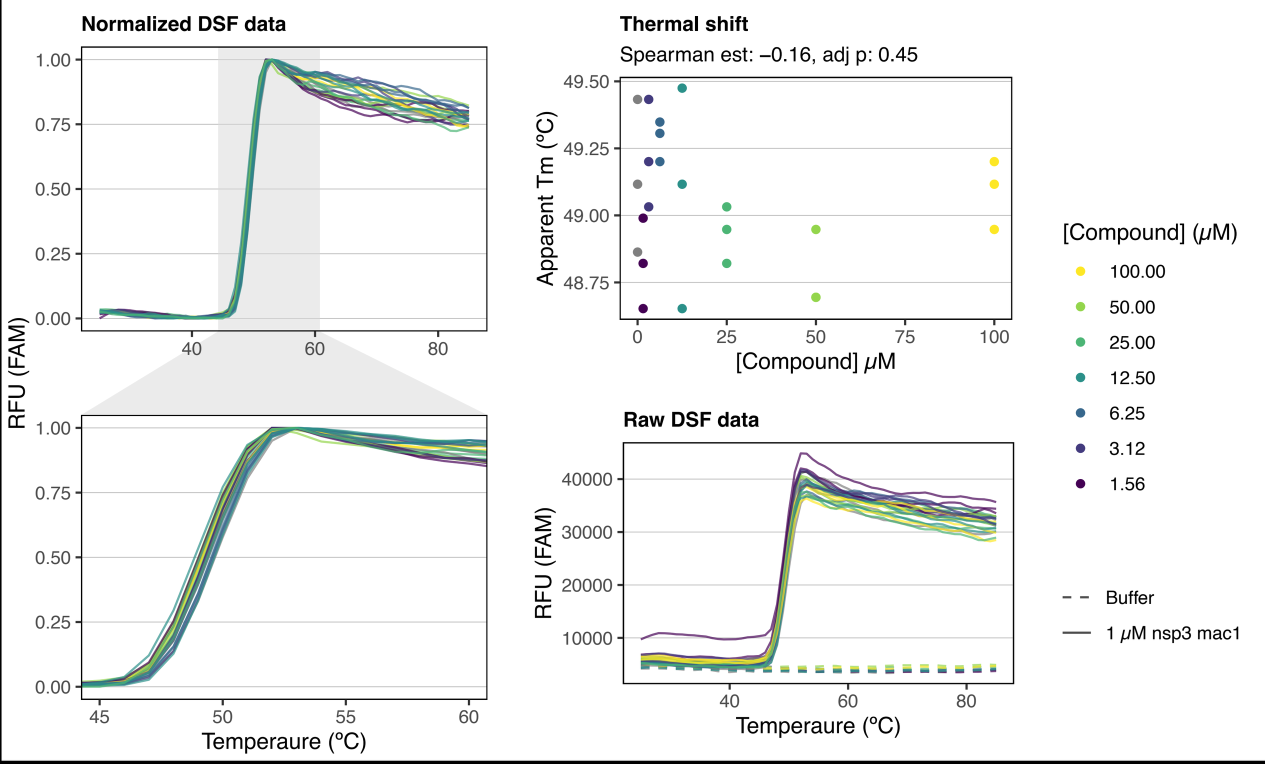

Compound: ZINC000273900063 (a.k.a Z0063) | Library: Mergers, Set 1

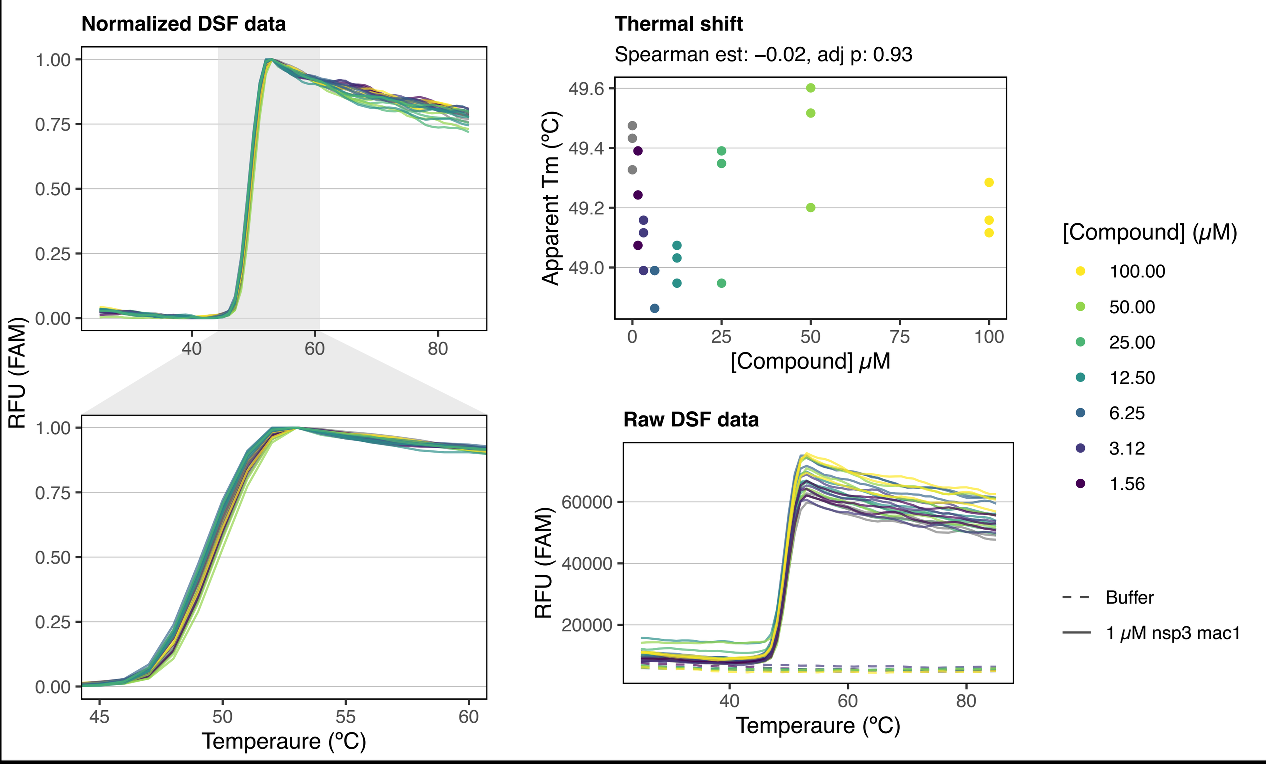

Compound: ZINC000359659274 (a.k.a Z9274) | Library: Mergers, Set 1

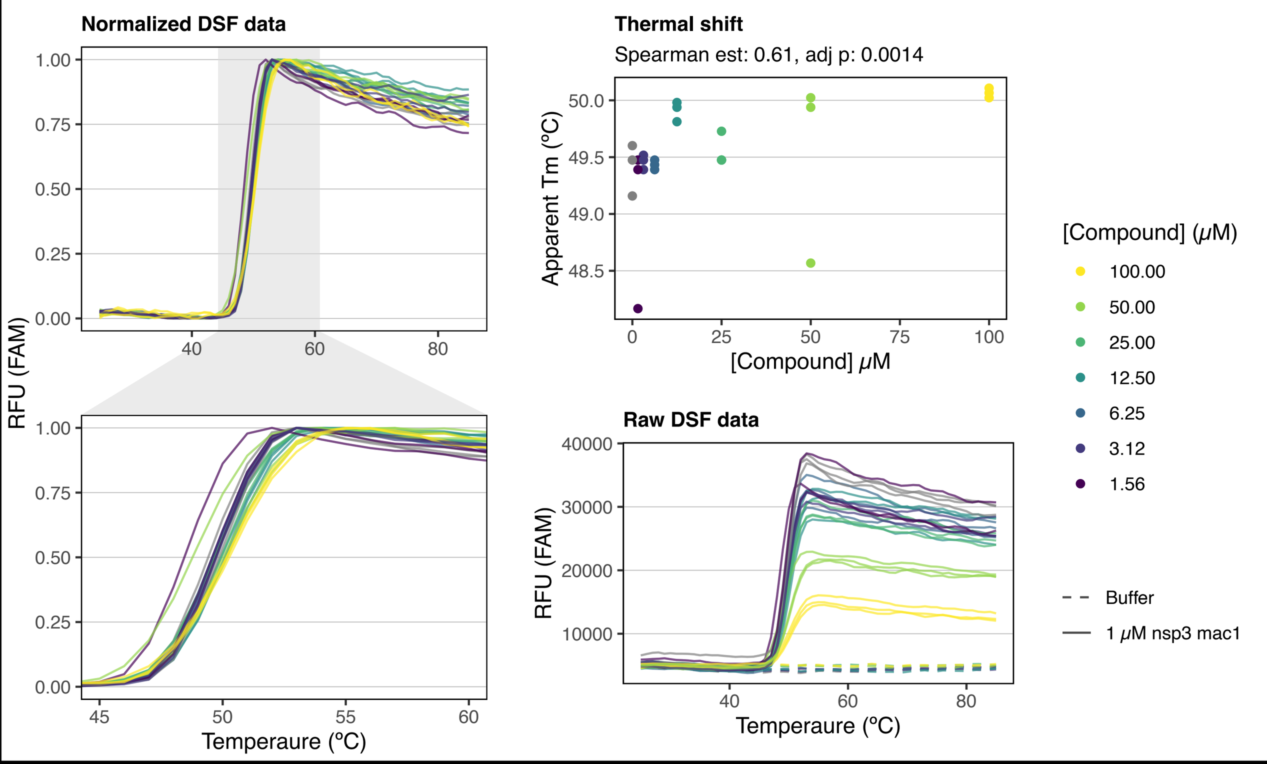

Compound: ZINC000611664196 (a.k.a Z4196) | Library: Mergers, Set 1

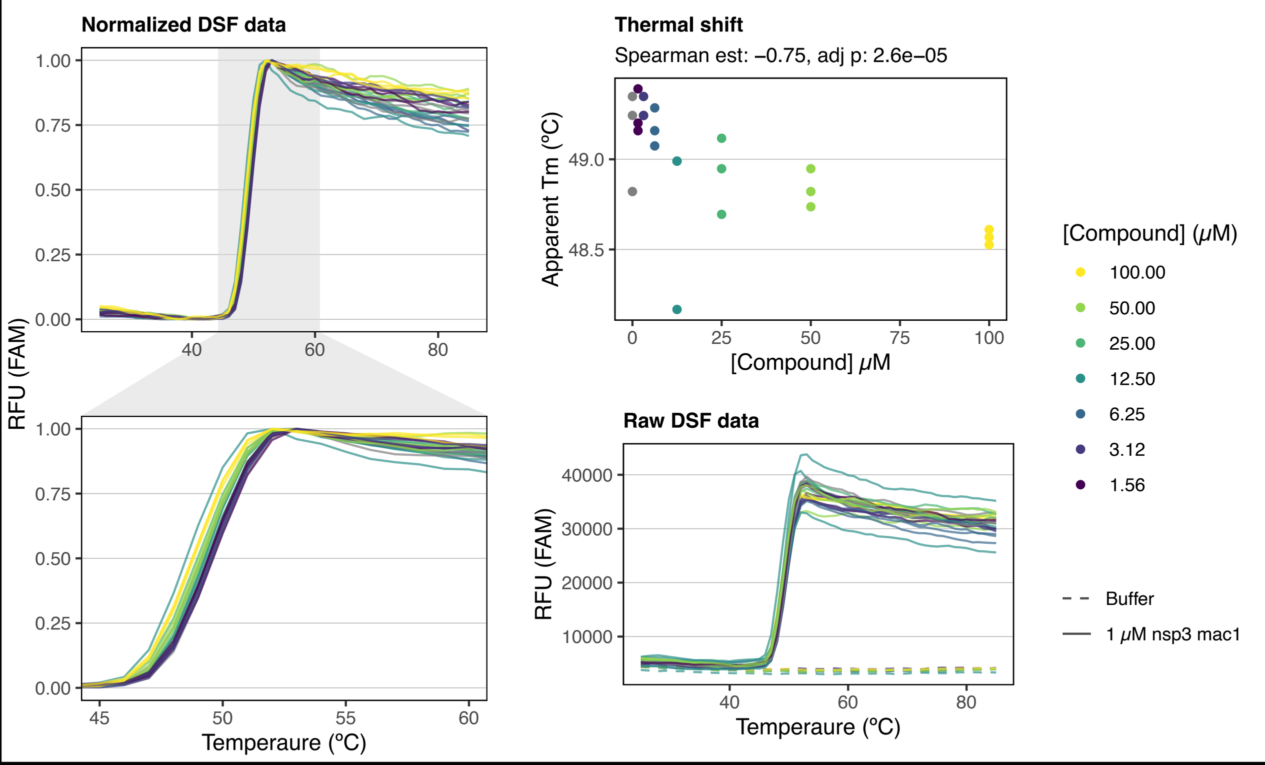

Compound: ZINC000751938095 (a.k.a Z8095) | Library: Mergers, Set 1

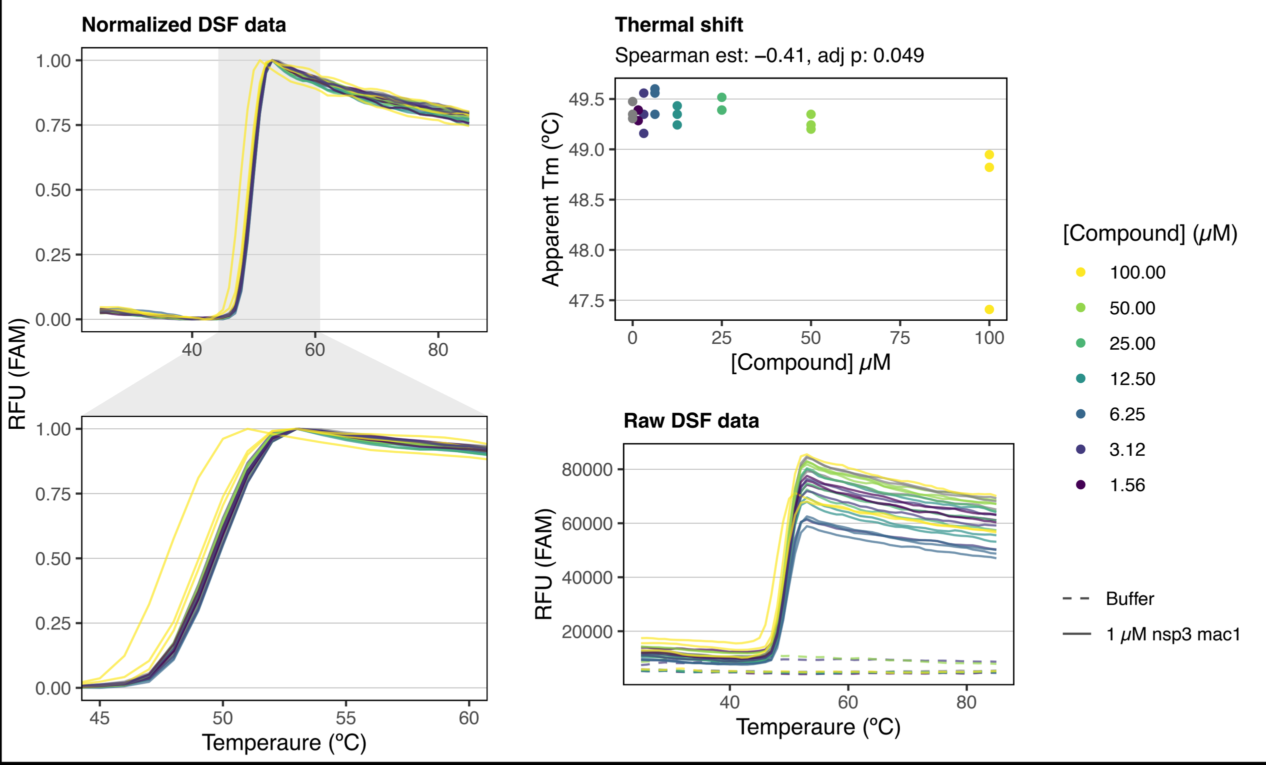

Compound: ZINC000833624464 (a.k.a Z4464) | Library: Mergers, Set 1

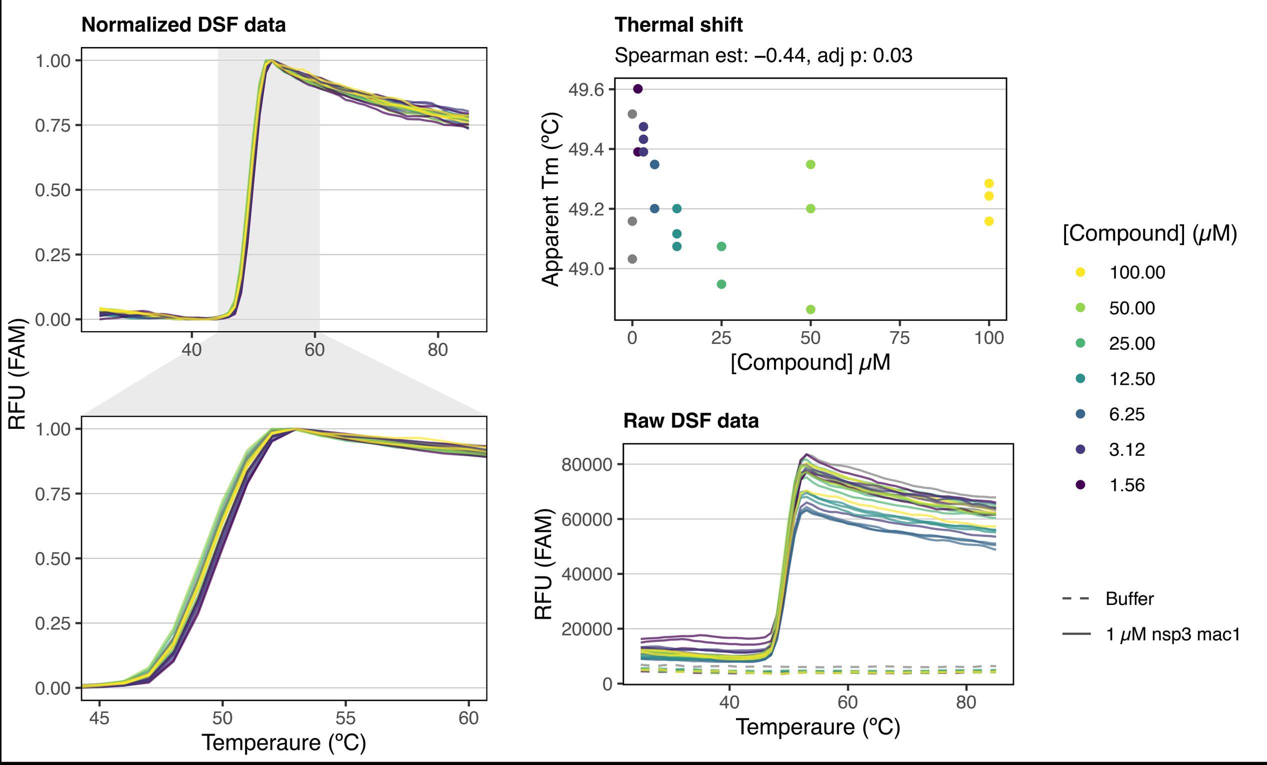

Compound: ZINC000850008207 (a.k.a Z8207) | Library: Mergers, Set 1

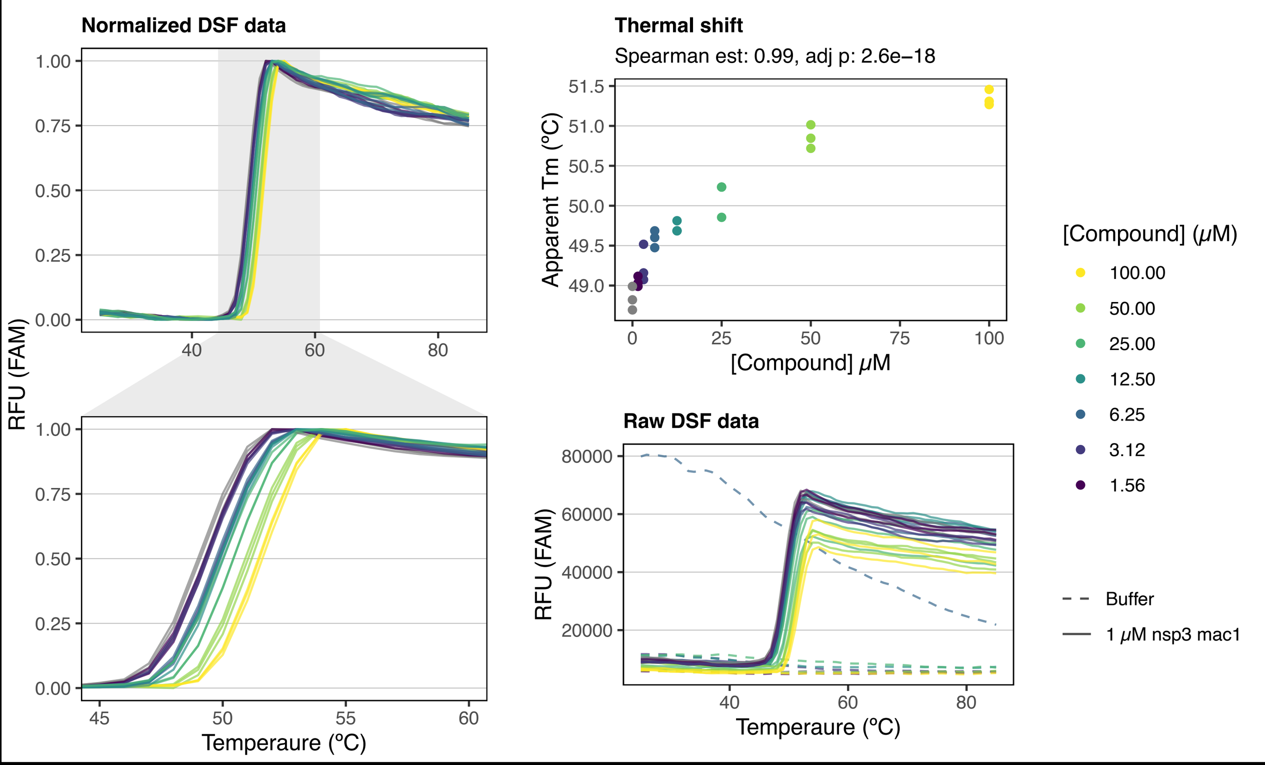

Compound: ZINC000885048835 (a.k.a Z8835) | Library: Mergers, Set 1

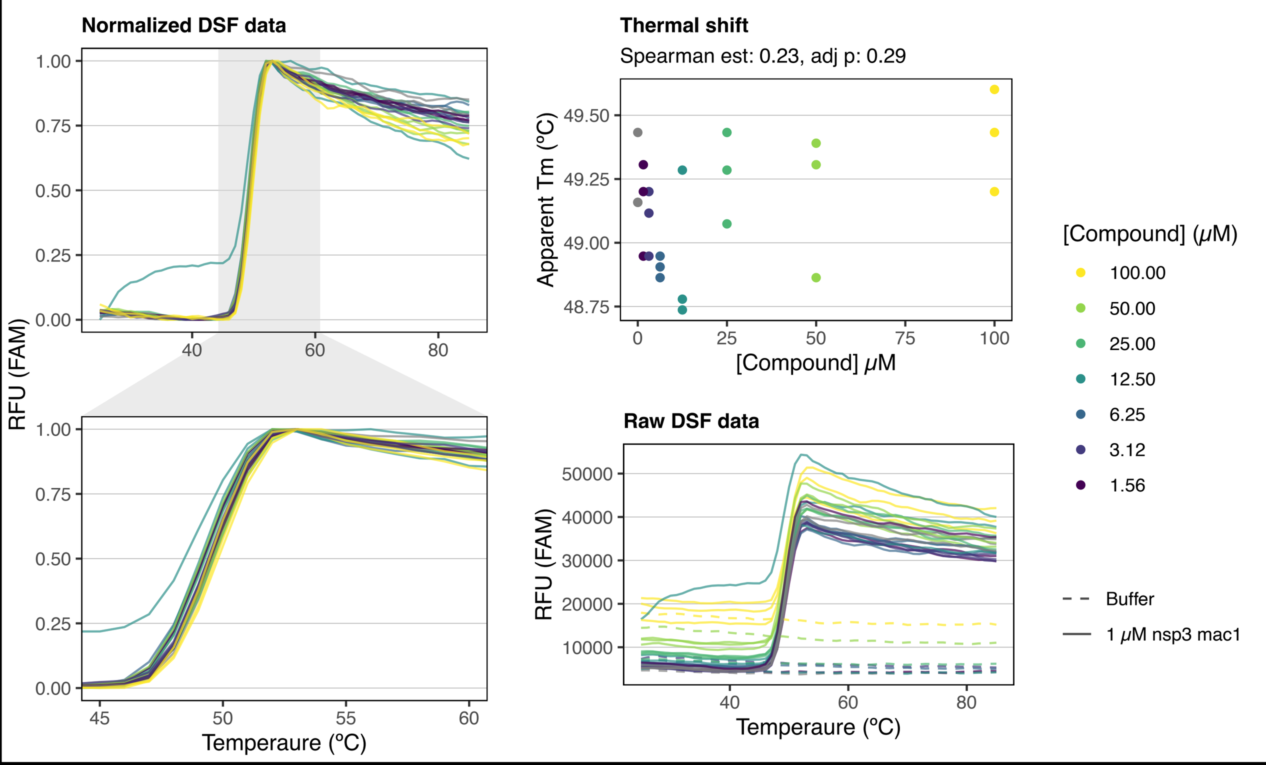

Compound: ZINC000891943247 (a.k.a Z3247) | Library: Mergers, Set 1

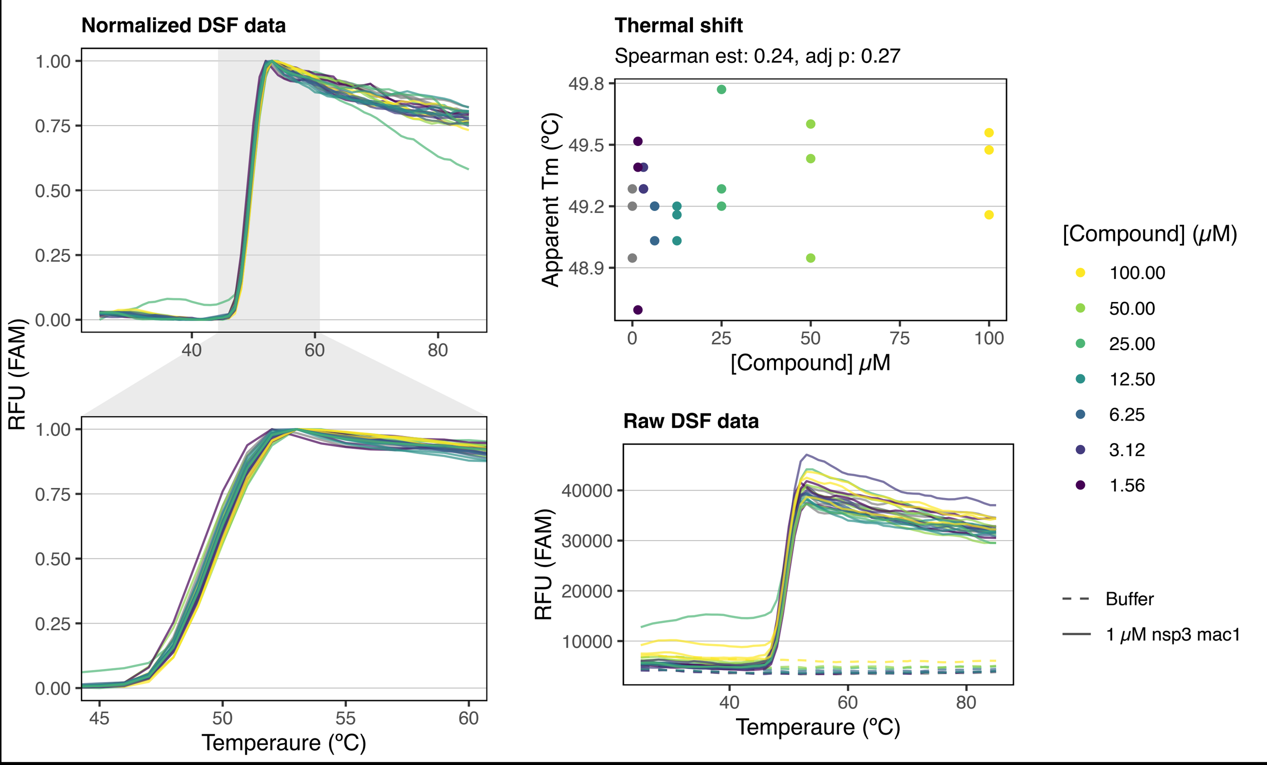

Compound: ZINC000893101964 (a.k.a Z1964) | Library: Mergers, Set 1

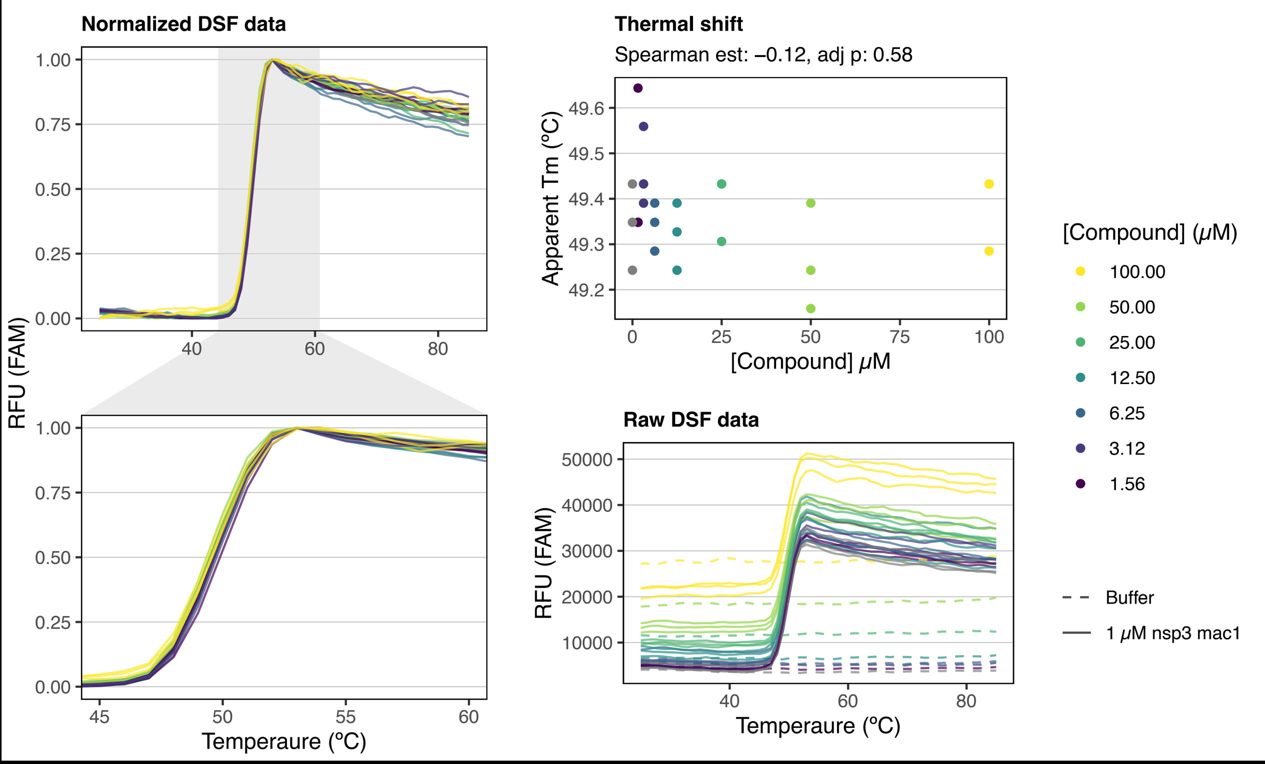

Compound: ZINC000949397098 (a.k.a Z7098) | Library: Mergers, Set 1

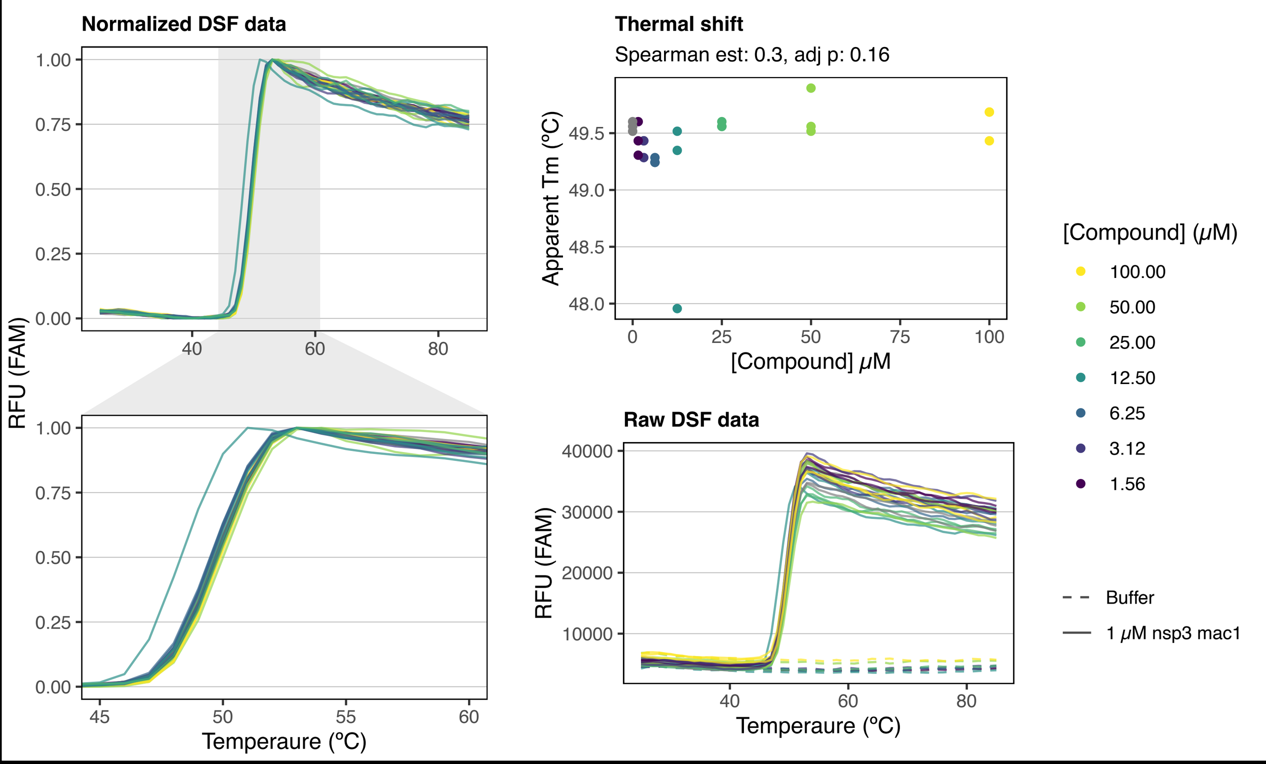

Compound: ZINC001119059028 (a.k.a Z9028) | Library: Mergers, Set 1

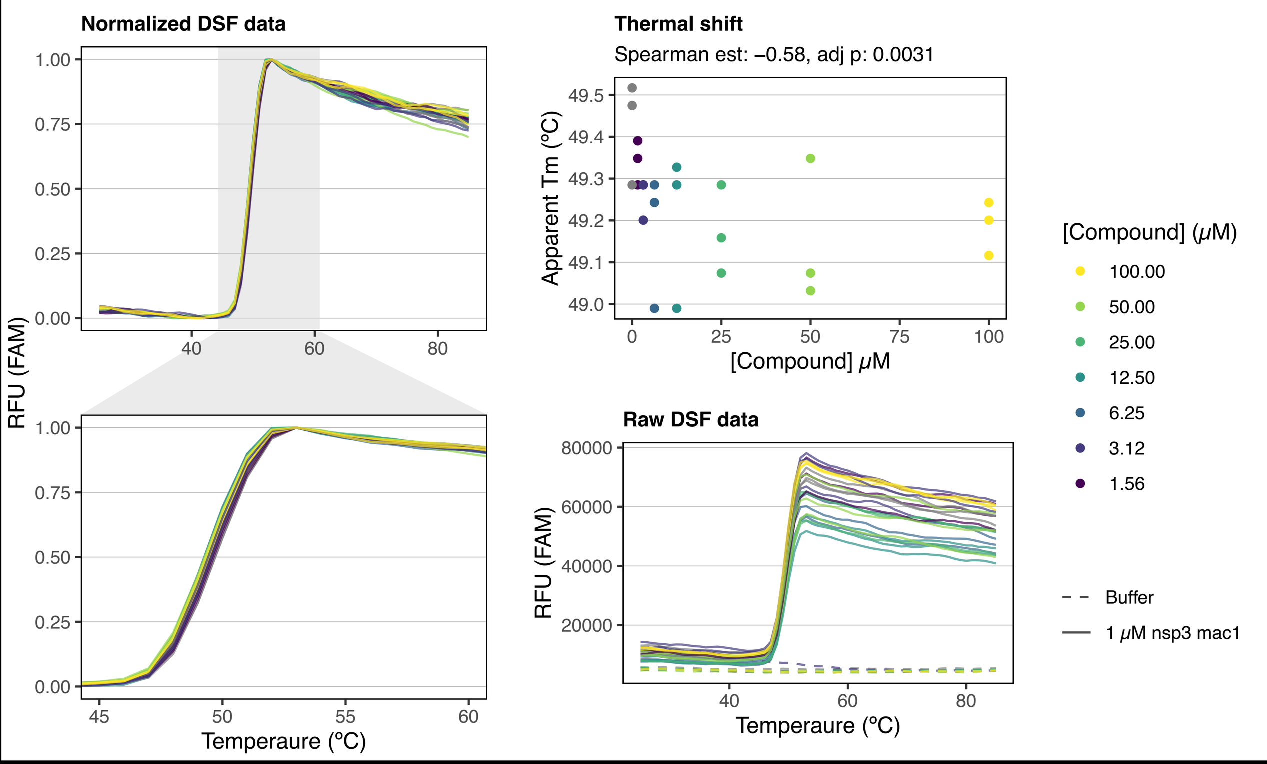

Compound: ZINC001240411747 (a.k.a Z1747) | Library: Mergers, Set 1

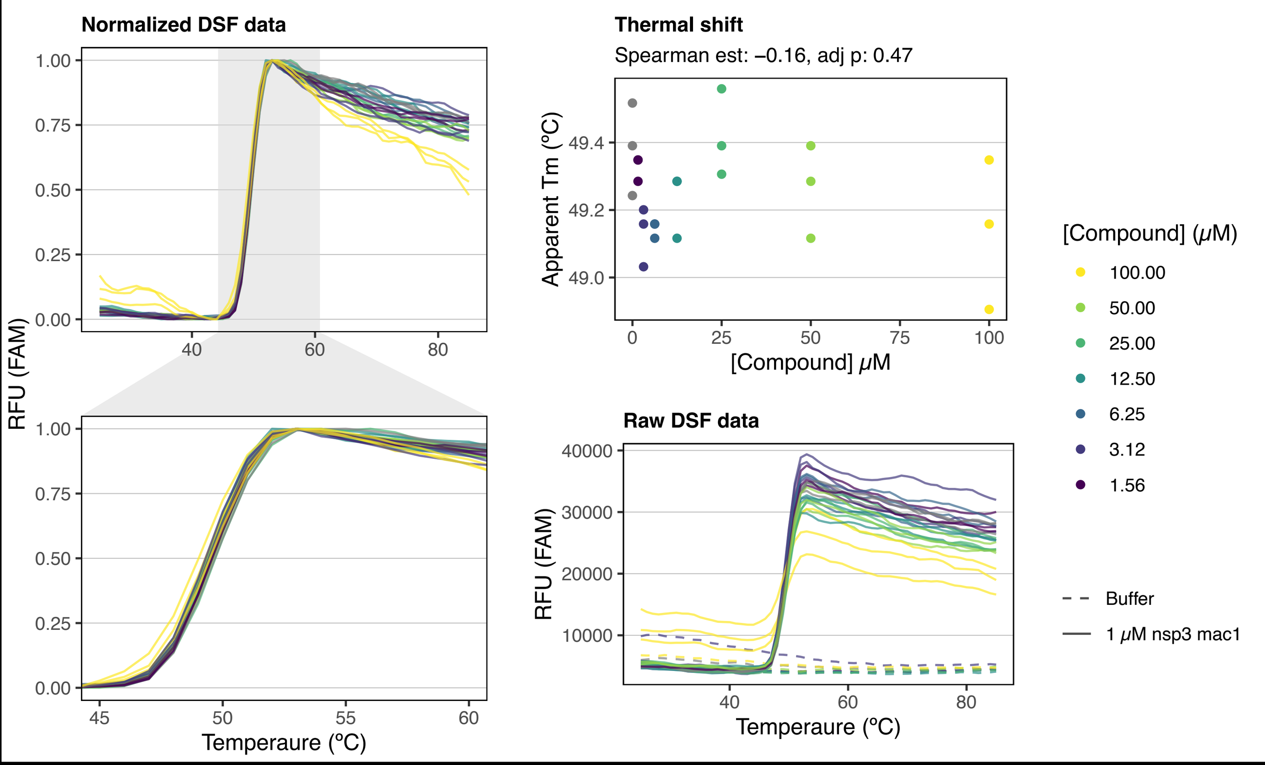

Compound: ZINC001320848619 (a.k.a Z8619) | Library: Mergers, Set 1

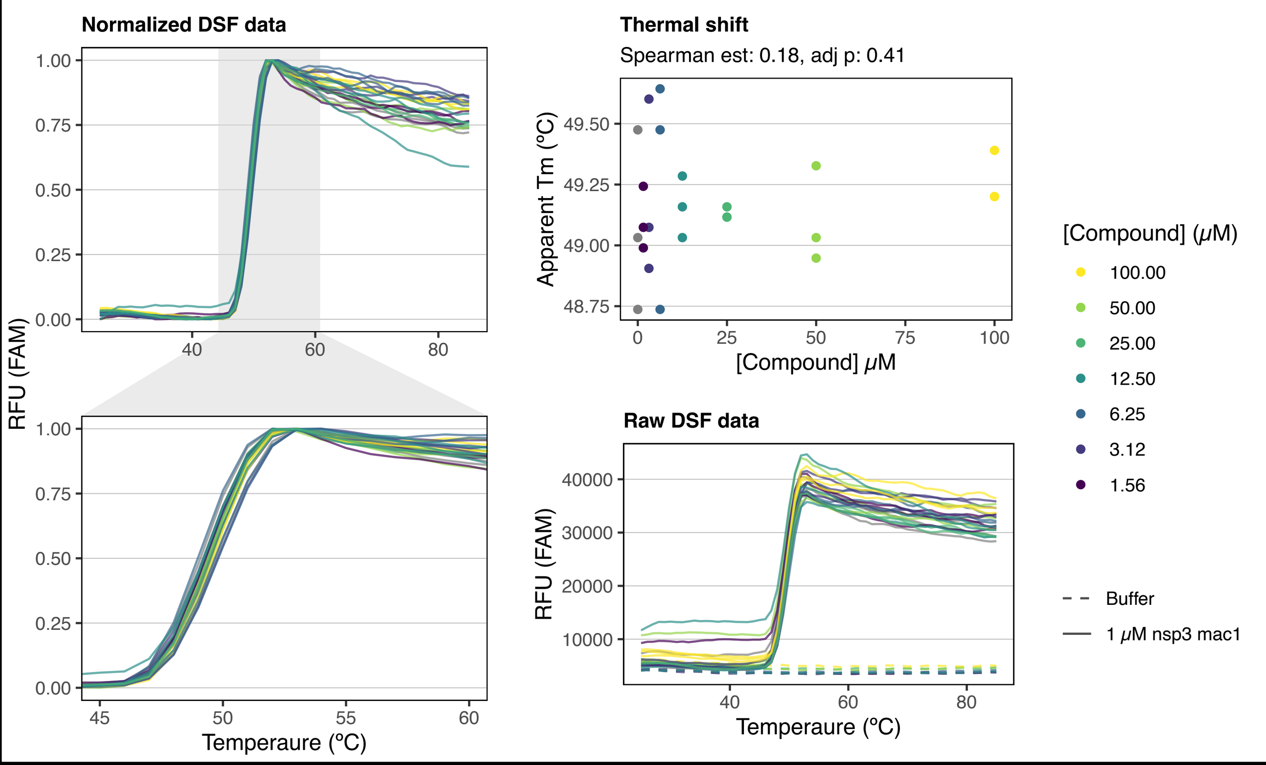

Compound: ZINC001327823432 (a.k.a Z3432) | Library: Mergers, Set 1

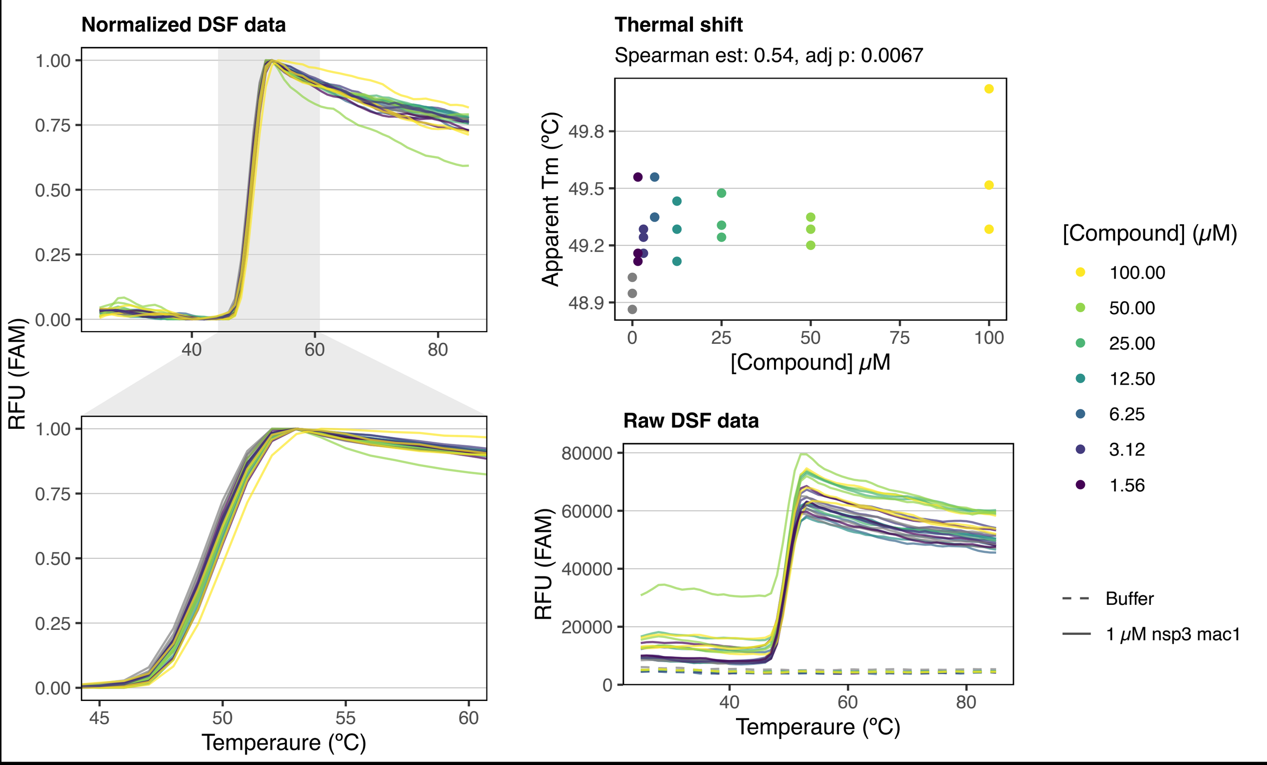

Compound: ZINC001335091110 (a.k.a Z1110) I Library: Mergers, Set 1

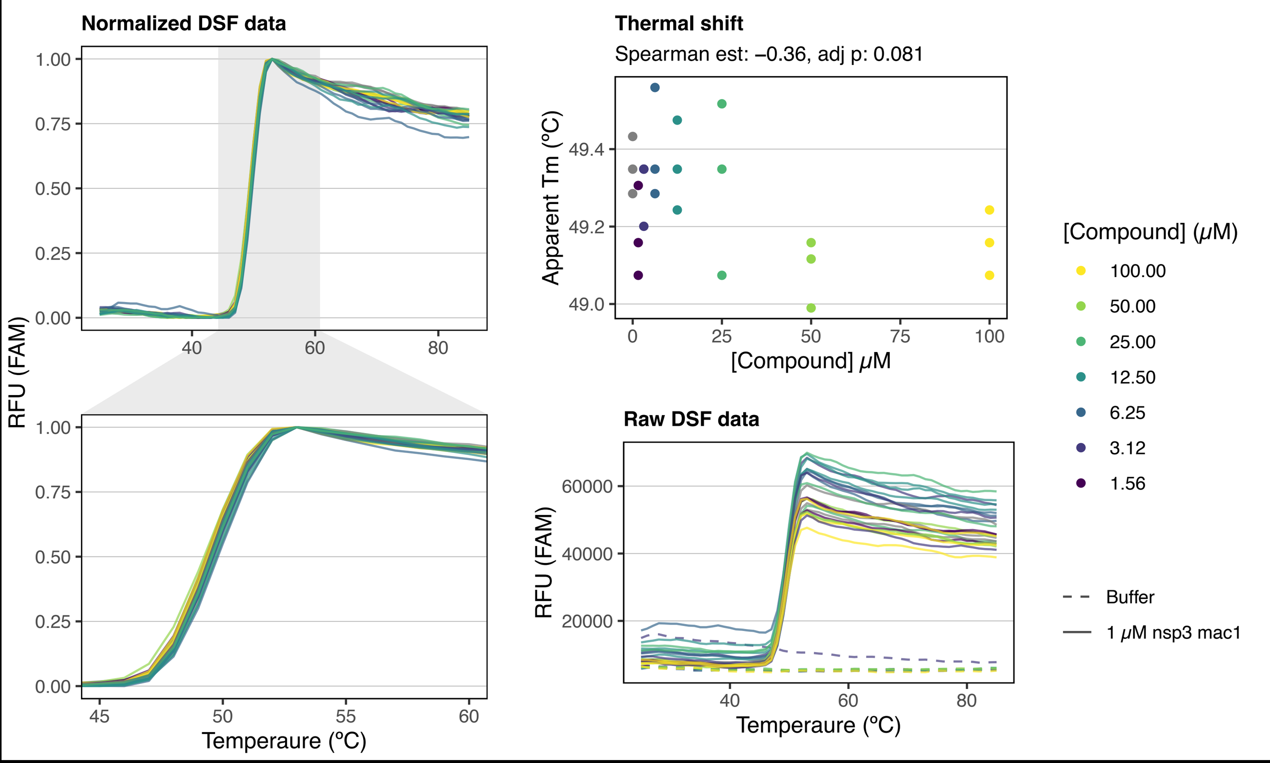

Compound: ZINC001364774273 (a.k.a Z4273) I Library: Mergers, Set 1

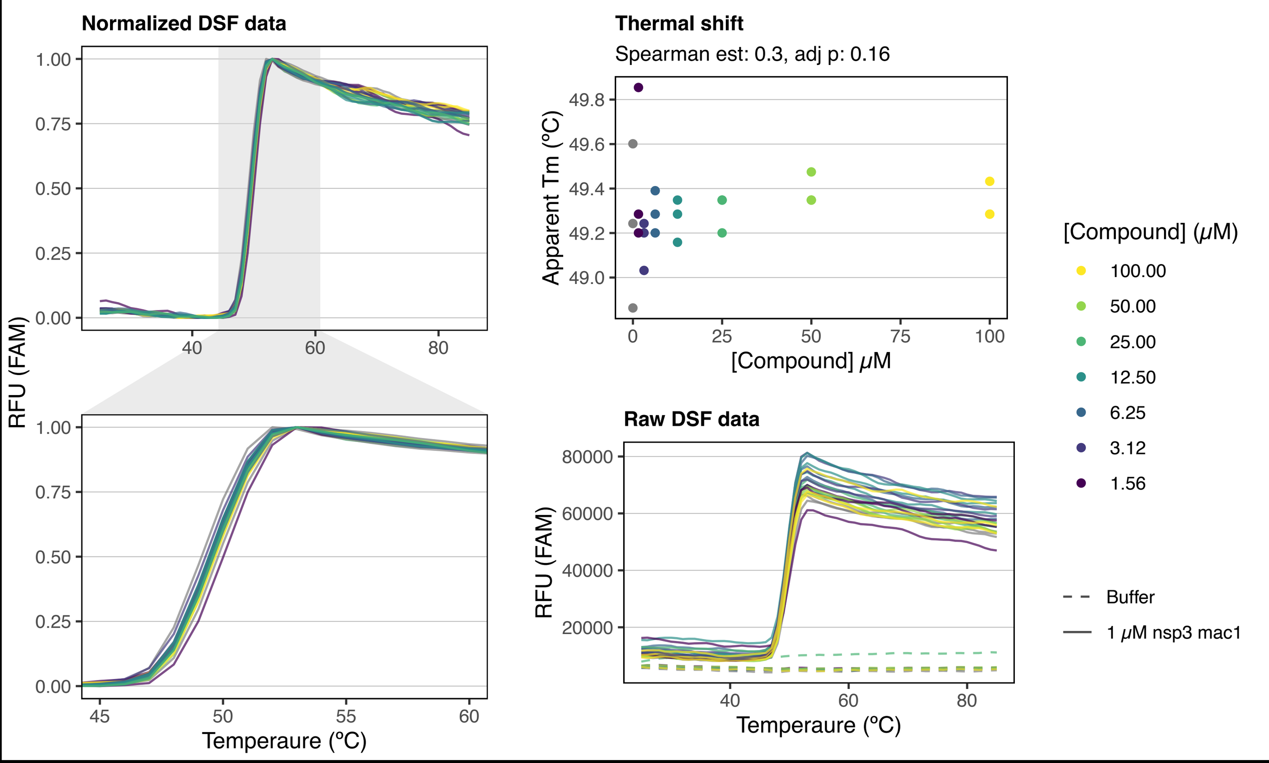

Compound: ZINC001434309614 (a.k.a Z9614) I Library: Mergers, Set 1

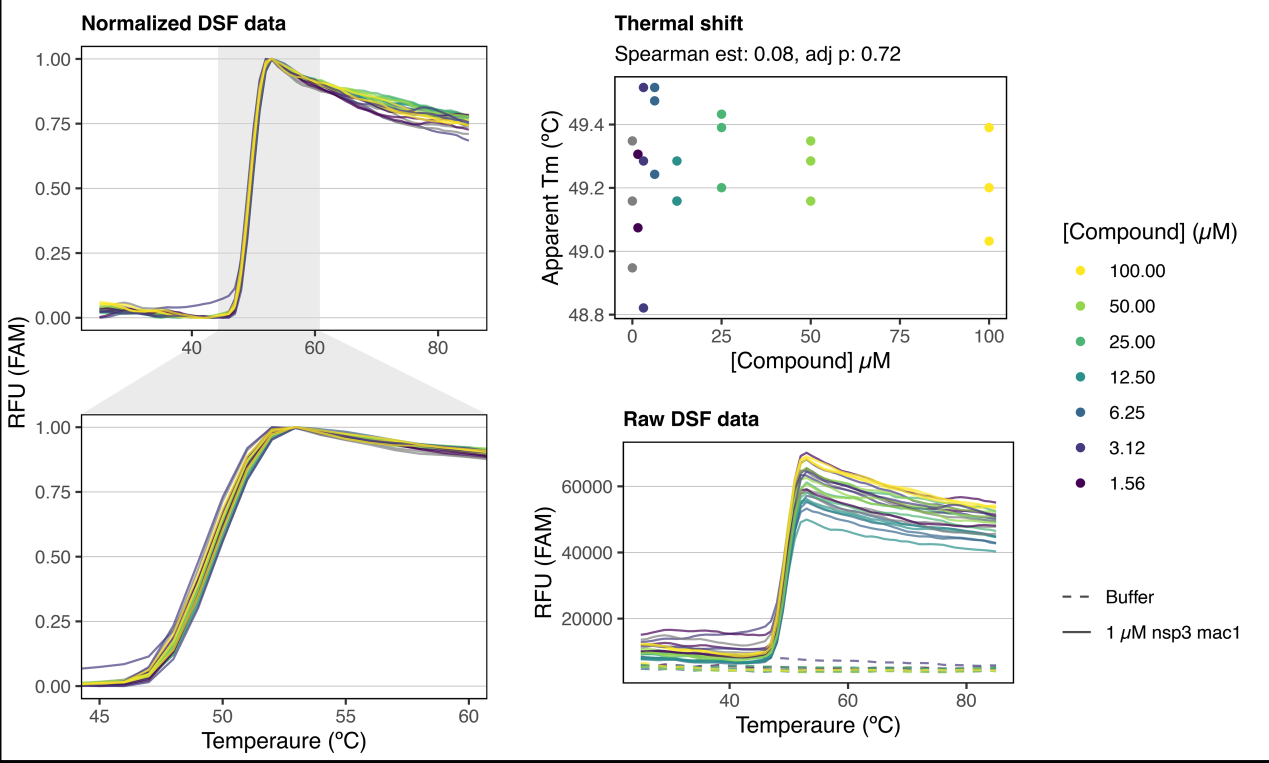

Compound: FRESH00004674769 (a.k.a F4769) I Library: Mergers, Set 2

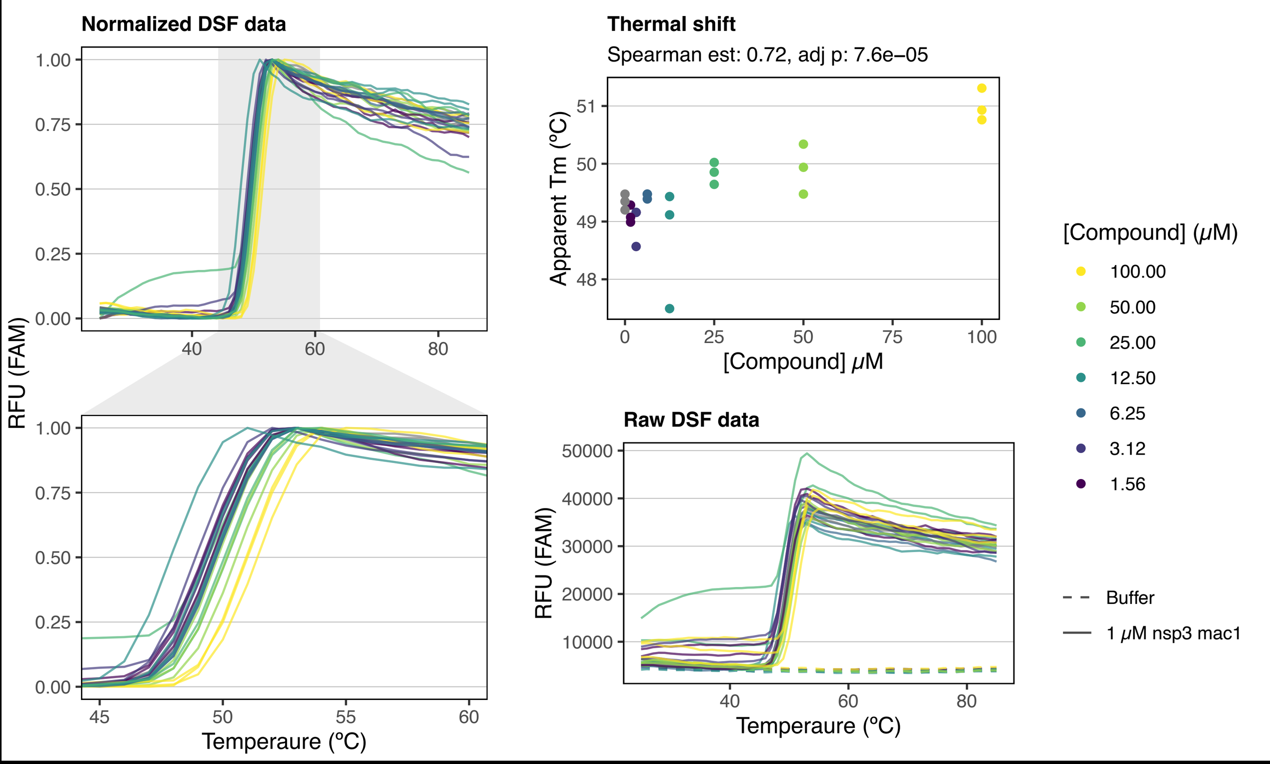

Compound: REAL250001448407 (a.k.a R8407) I Library: Mergers, Set 2

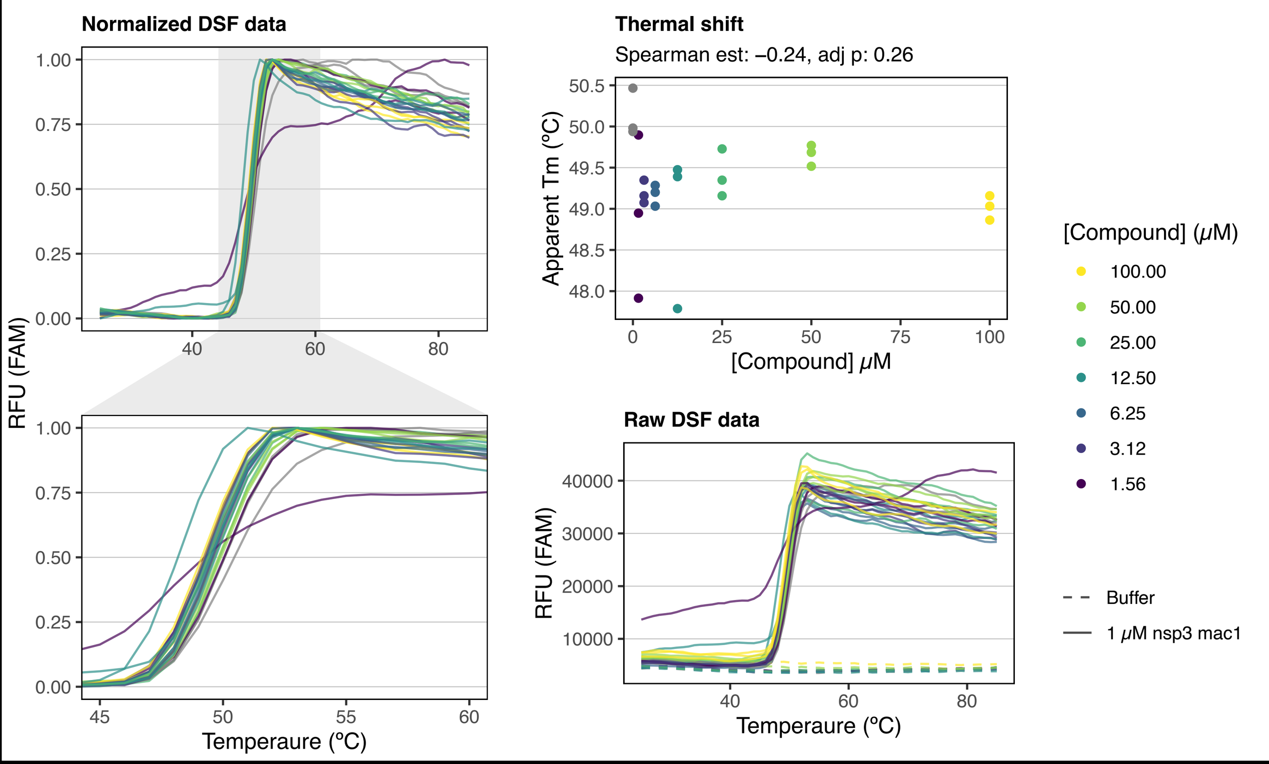

Compound: REAL250003774401 (a.k.a R4401) I Library: Mergers, Set 2

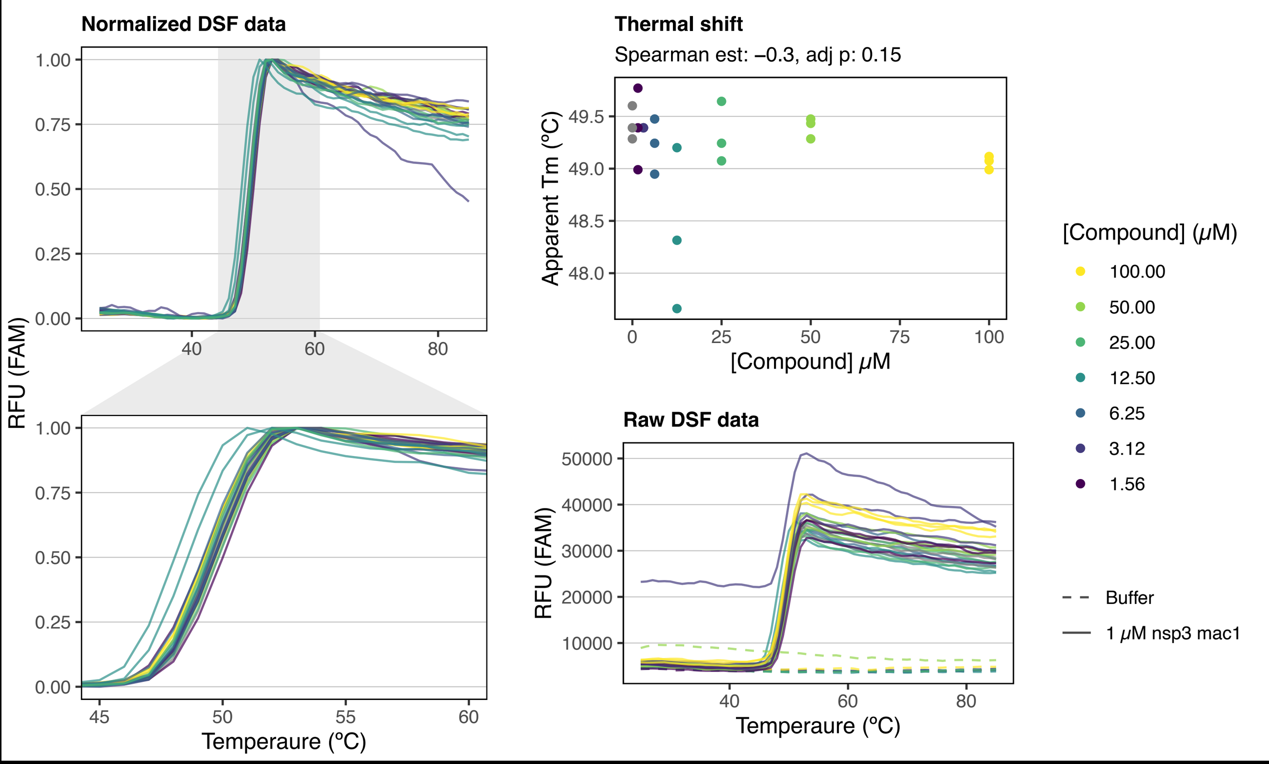

Compound: REAL250003958539 (a.k.a R8539) I Library: Mergers, Set 2

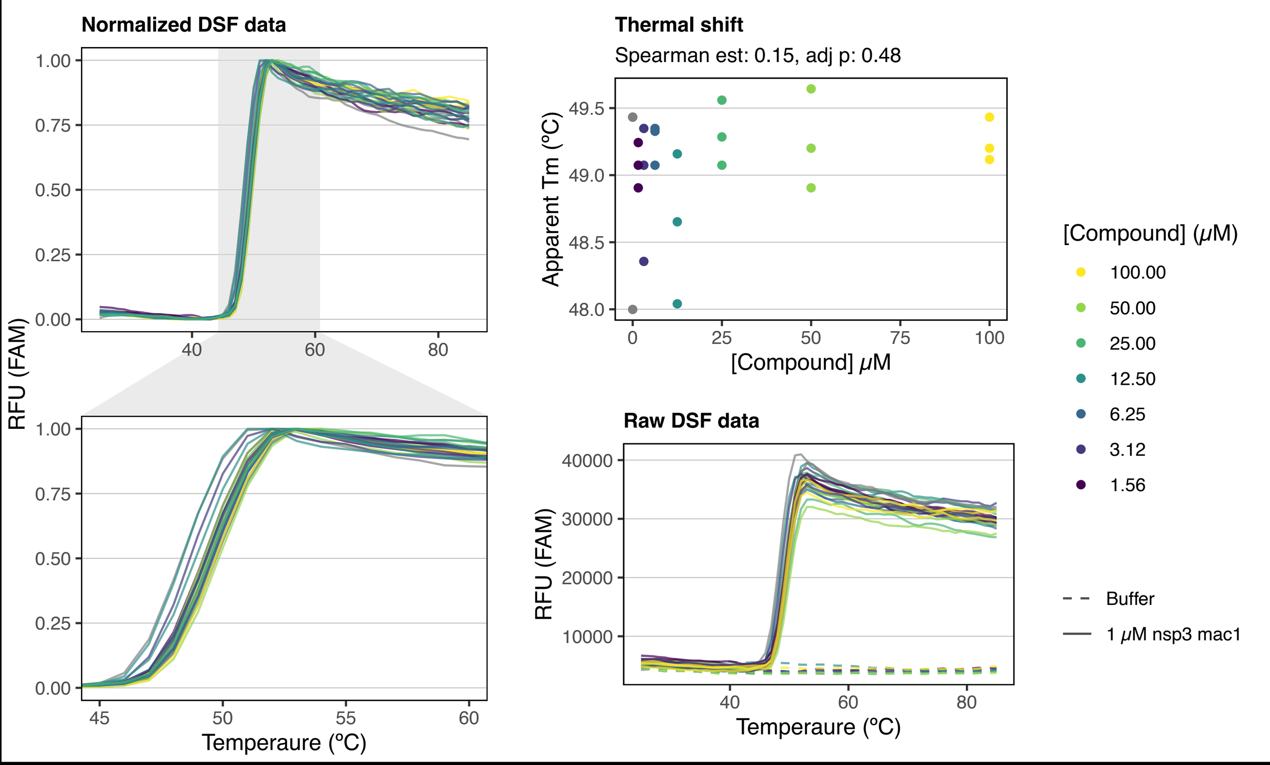

Compound: REAL300007260658 (a.k.a R0658) I Library: Mergers, Set 2

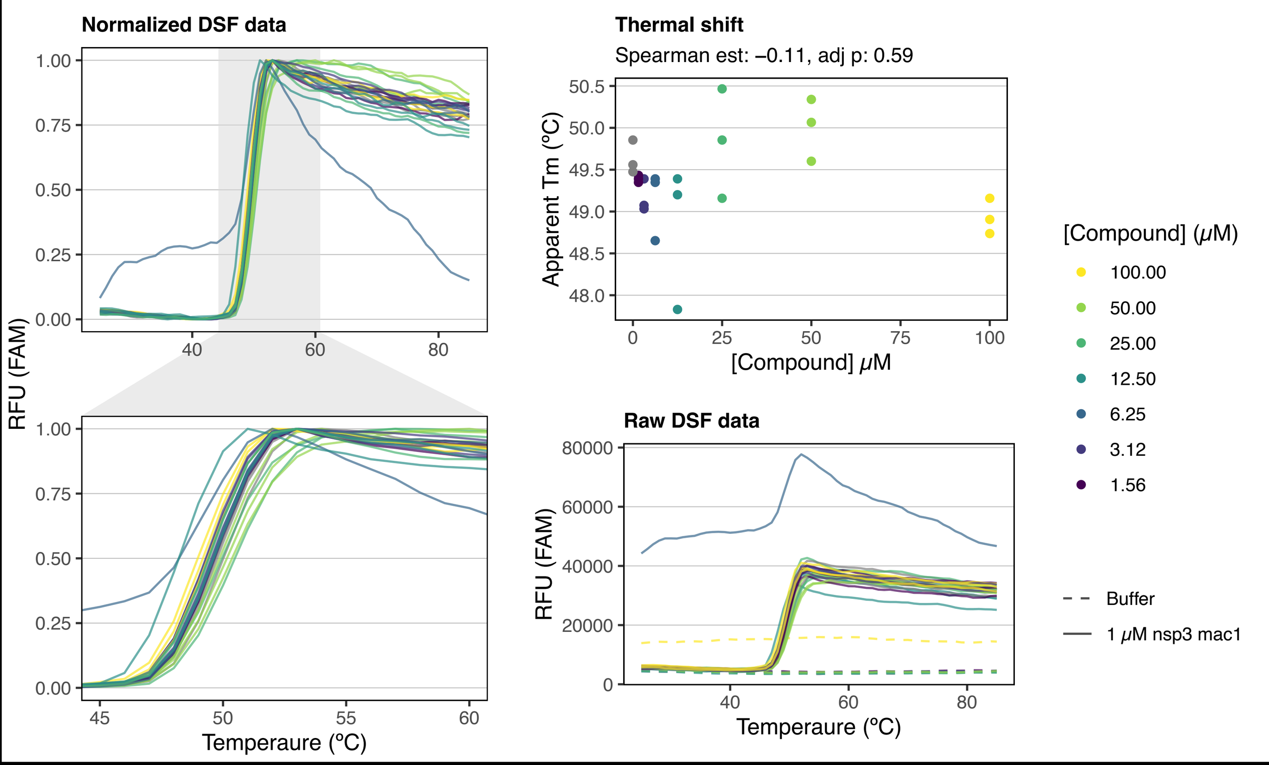

Compound: REAL300019621104 (a.k.a R1104) I Library: Mergers, Set 2

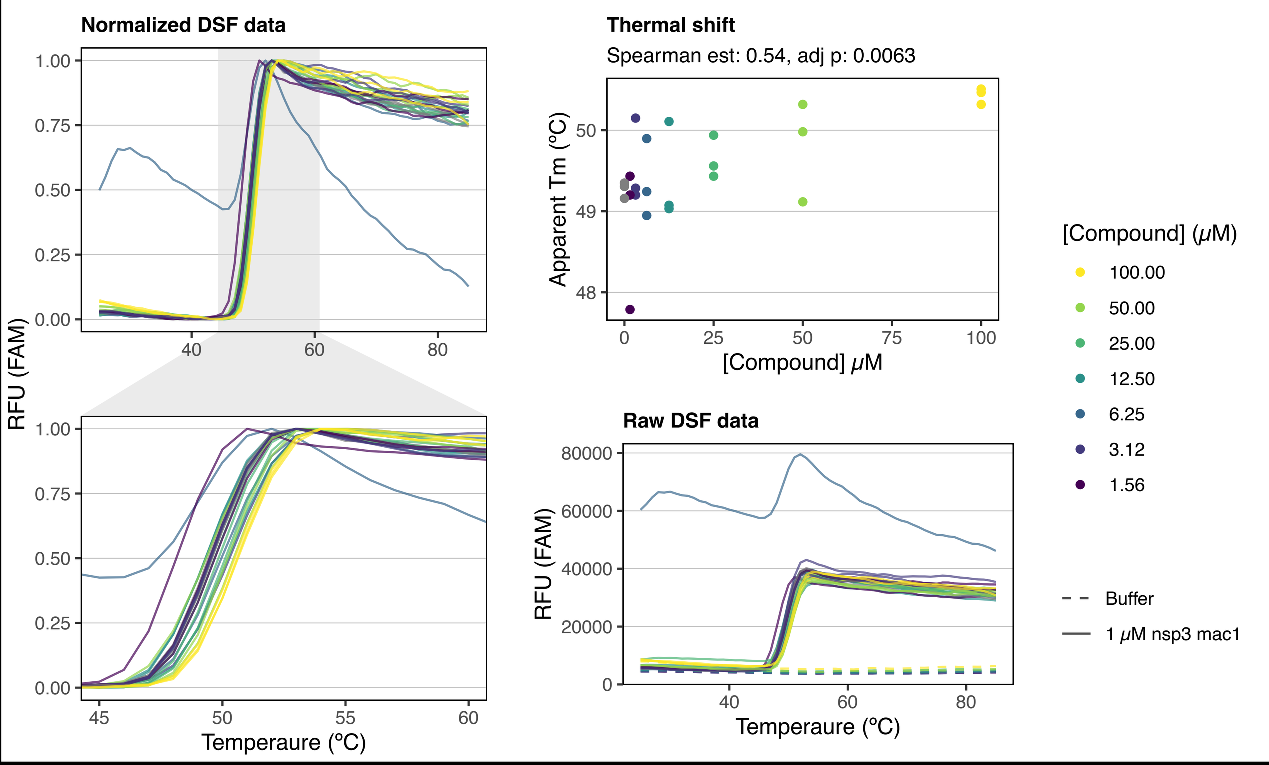

Compound: ZINC000183202483 (a.k.a Z2483) I Library: Mergers, Set 2

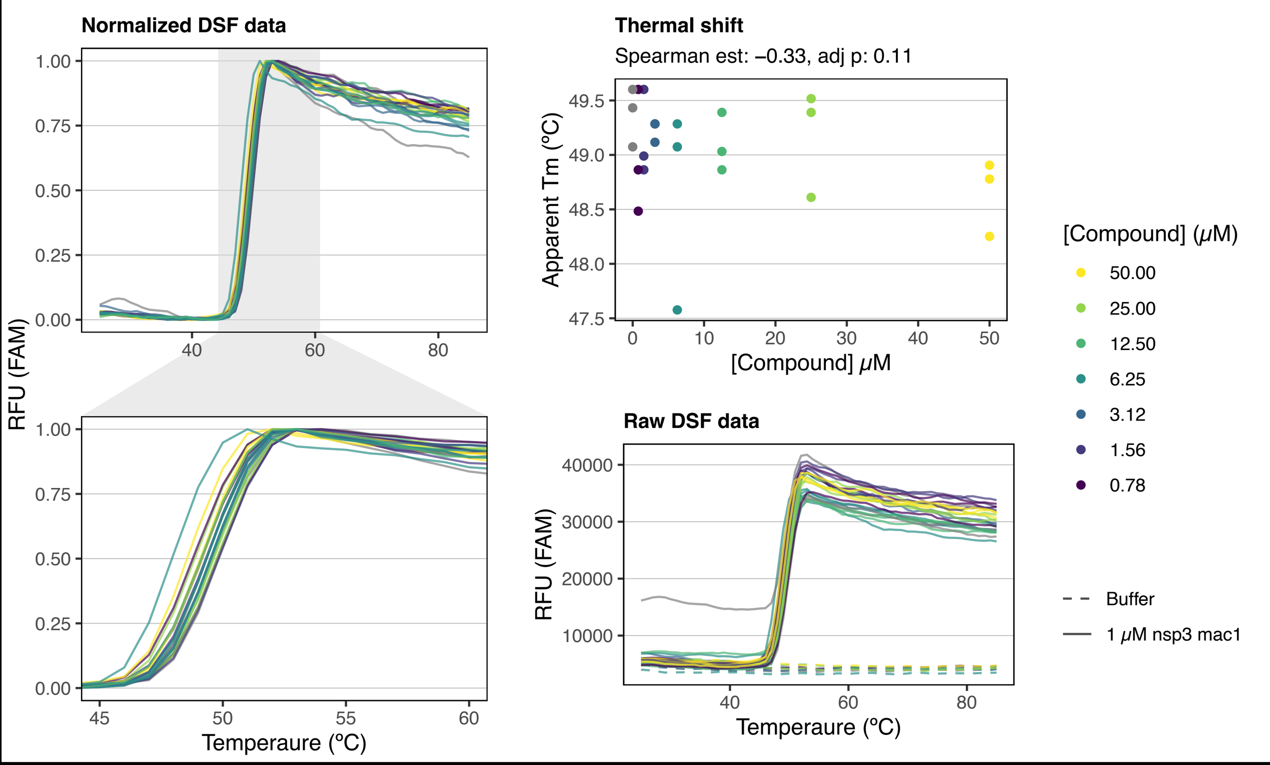

Compound: ZINC000904346960 (a.k.a Z6960) I Library: Mergers, Set 2

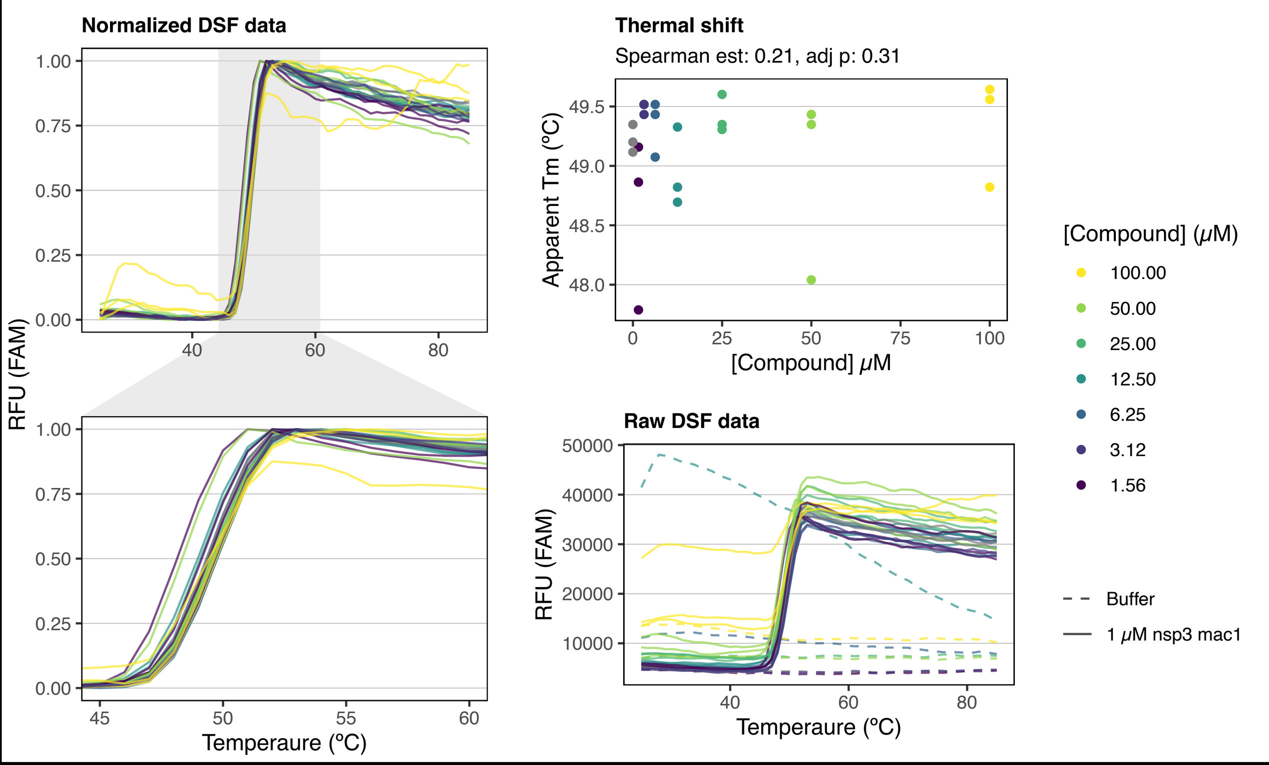

Compound: ZINC001156036512 (a.k.a Z6512) I Library: Mergers, Set 2

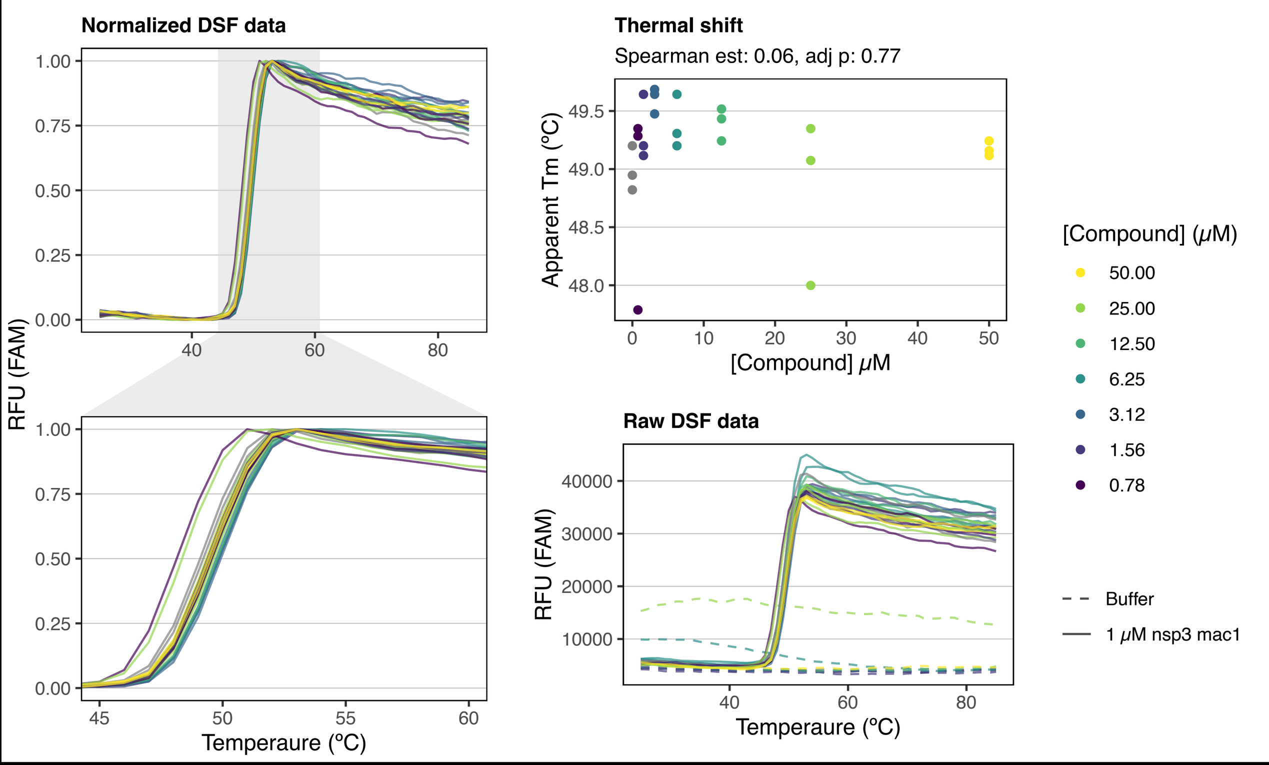

Compound: ZINC001240776402 (a.k.a Z6402) I Library: Mergers, Set 2

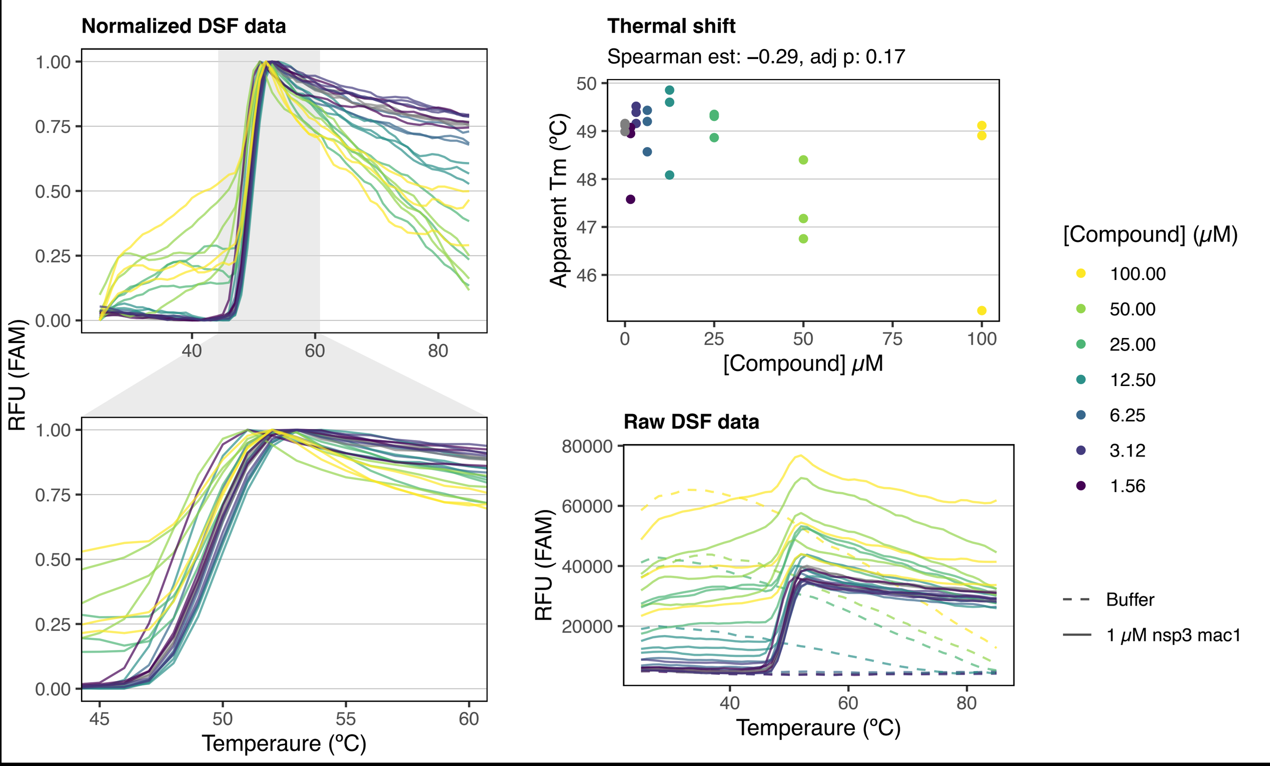

Compound: ZINC001365141527 (a.k.a Z1527) I Library: Mergers, Set 2

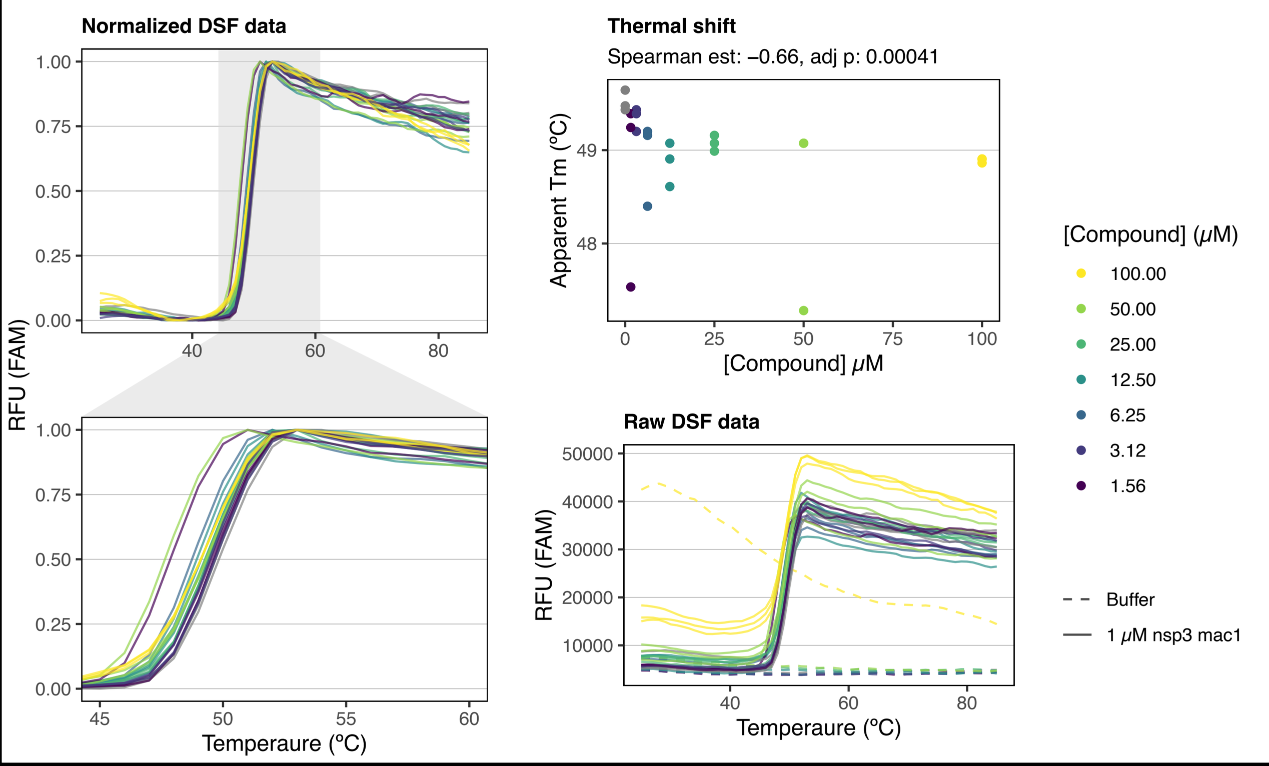

Compound: LL1_0016 (a.k.a LL1_0016) I Library: LL

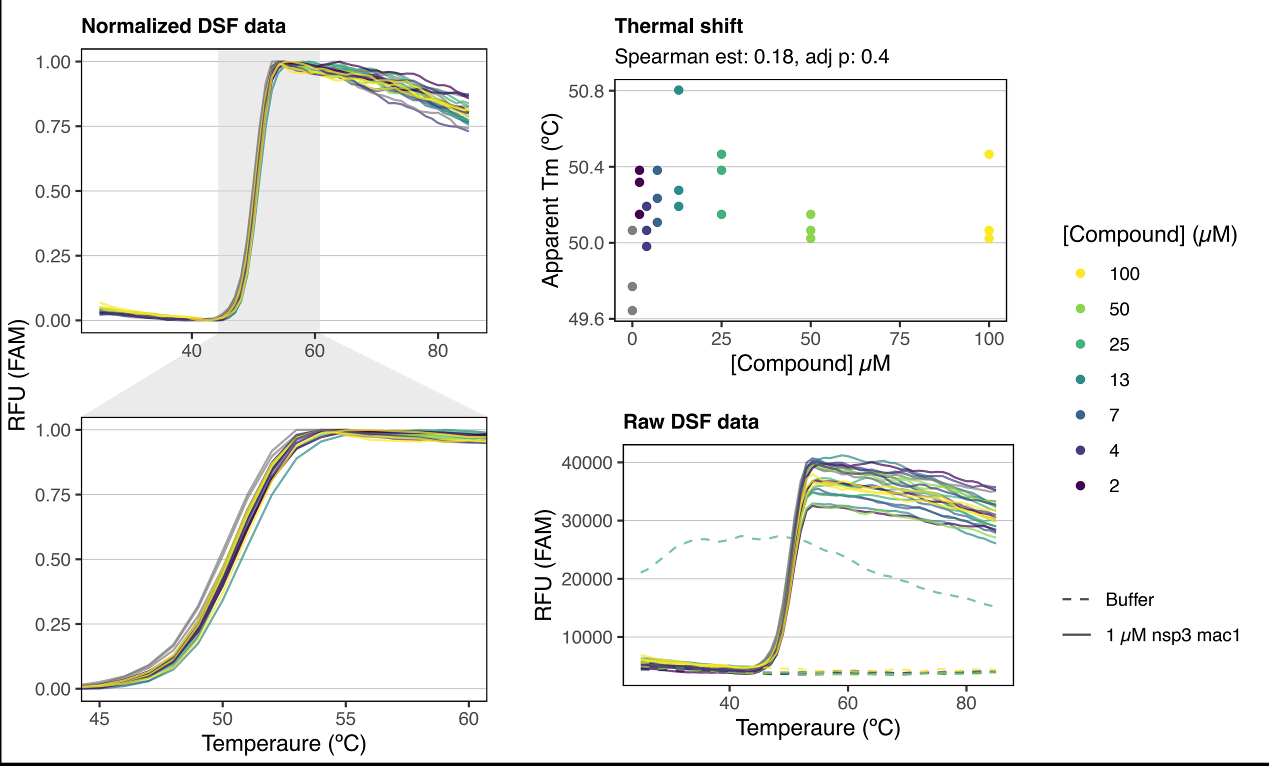

Compound: LL1_0010 (a.k.a LL1_0010) I Library: LL

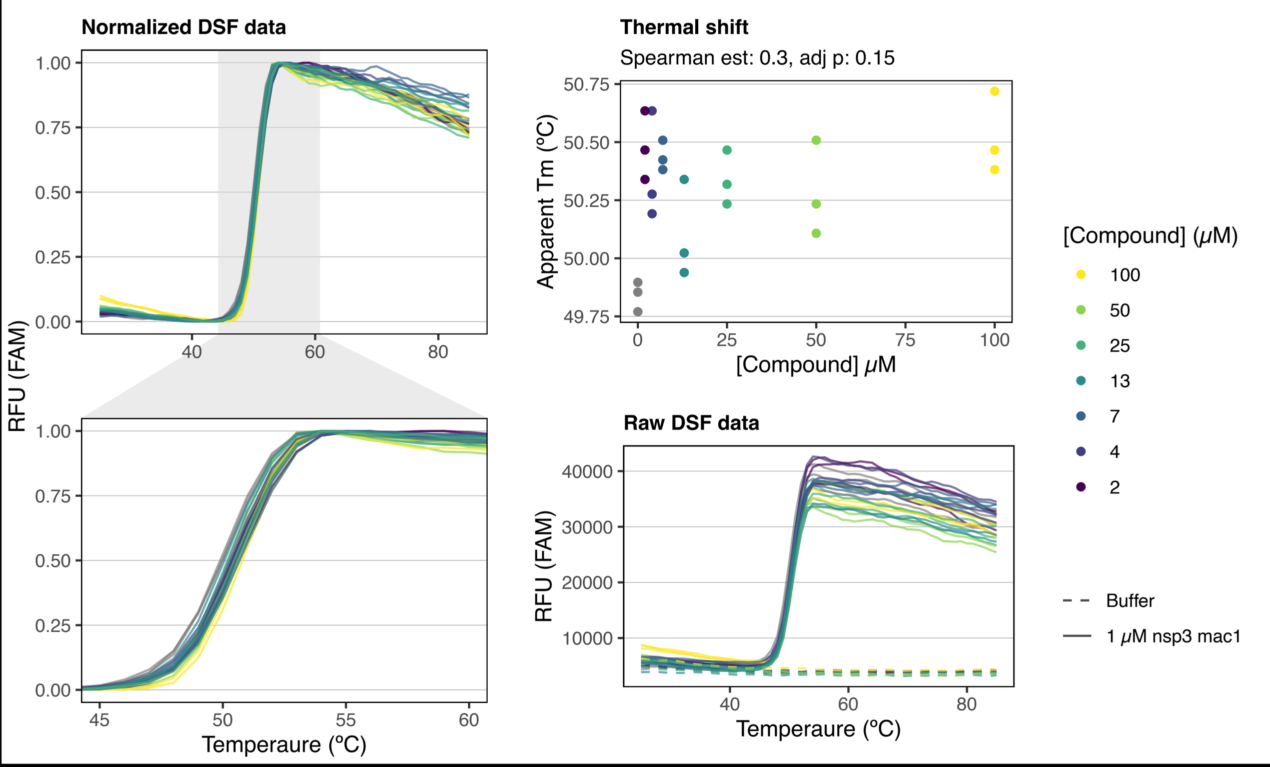

Compound: LL1_0005 (a.k.a LL1_0005) I Library: LL

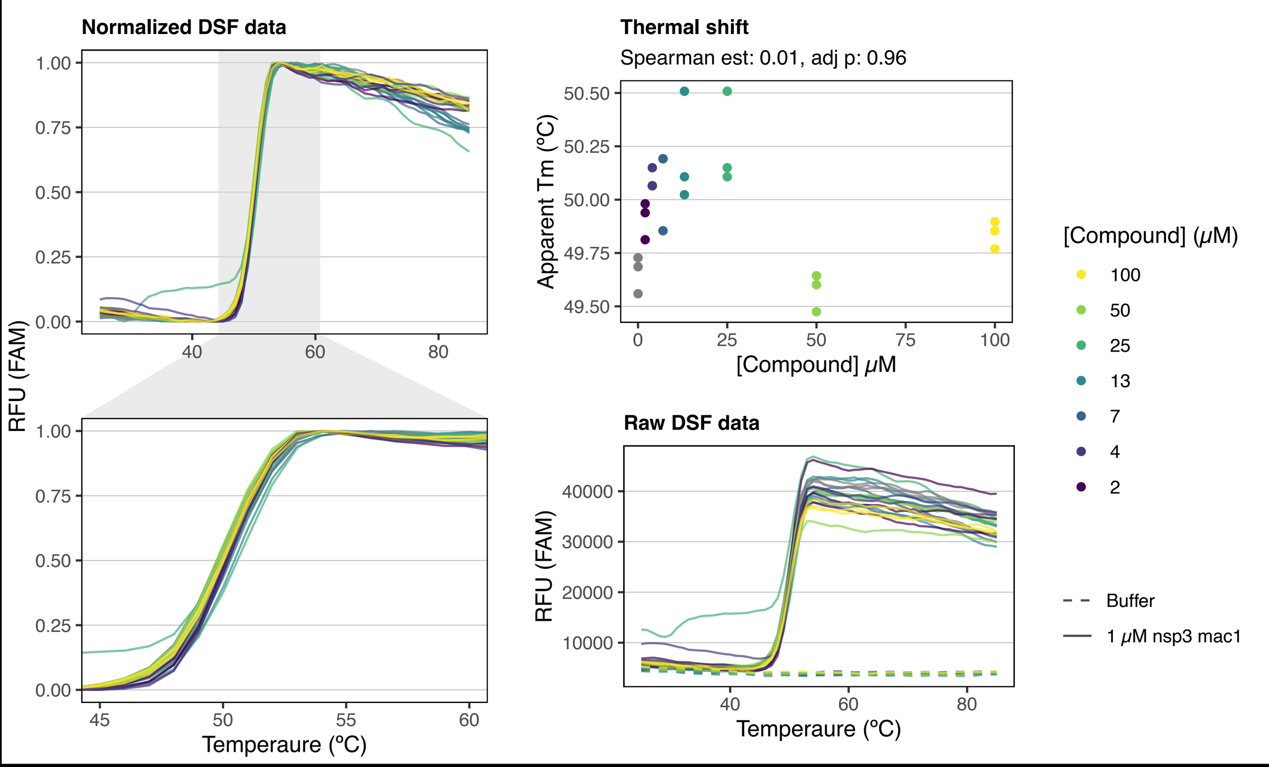

Compound: LL1_0017 (a.k.a LL1_0017) I Library: LL

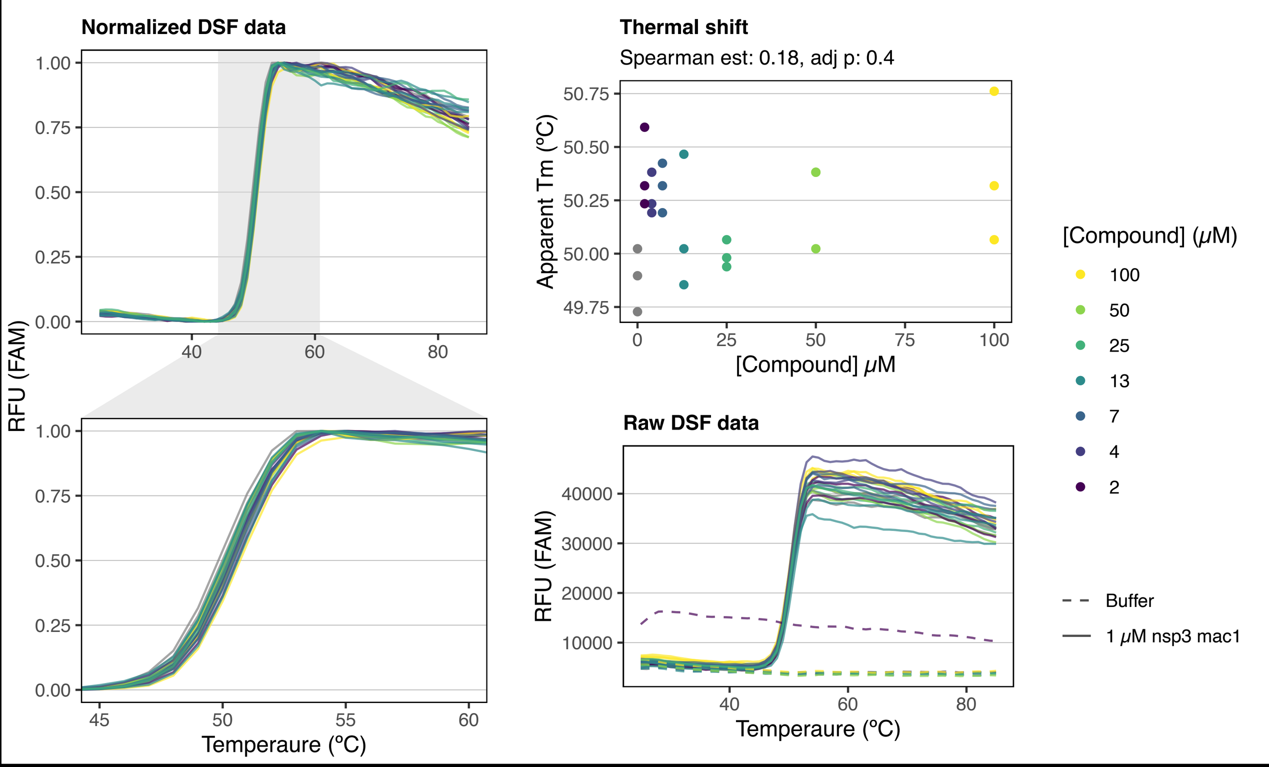

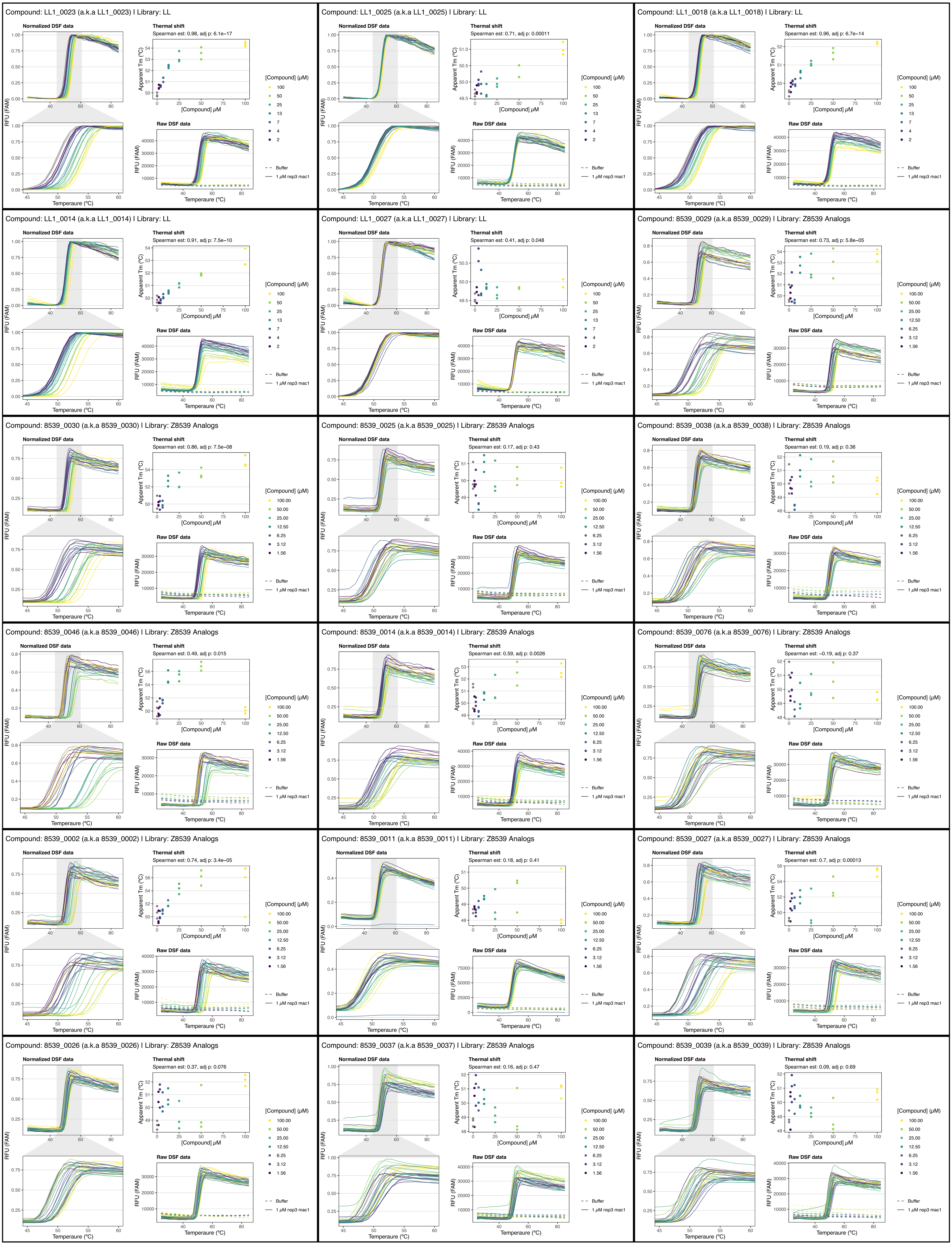
Compound: LL1_0023 (a.k.a LL1_0023) I Library: LL
Spearman est: 0.98, adj p: 6.1e−17
Compound: LL1_0025 (a.k.a LL1_0025) I Library: LL
Spearman est: 0.71, adj p: 0.00011
Compound: LL1_0018 (a.k.a LL1_0018) I Library: LL
Spearman est: 0.96, adj p: 6.7e−14
Compound: LL1_0014 (a.k.a LL1_0014) I Library: LL
Spearman est: 0.91, adj p: 7.5e−10
Compound: LL1_0027 (a.k.a LL1_0027) I Library: LL
Spearman est: 0.41, adj p: 0.048
Compound: 8539_0029 (a.k.a 8539_0029) I Library: Z8539 Analogs
Spearman est: 0.73, adj p: 5.8e−05
Compound: 8539_0030 (a.k.a 8539_0030) I Library: Z8539 Analogs
Spearman est: 0.86, adj p: 7.5e−08
Compound: 8539_0025 (a.k.a 8539_0025) I Library: Z8539 Analogs
Spearman est: 0.17, adj p: 0.43
Compound: 8539_0038 (a.k.a 8539_0038) I Library: Z8539 Analogs
Spearman est: 0.19, adj p: 0.36
Compound: 8539_0046 (a.k.a 8539_0046) I Library: Z8539 Analogs
Spearman est: 0.49, adj p: 0.015
Compound: 8539_0014 (a.k.a 8539_0014) I Library: Z8539 Analogs
Spearman est: 0.59, adj p: 0.0026
Compound: 8539_0076 (a.k.a 8539_0076) I Library: Z8539 Analogs
Spearman est: −0.19, adj p: 0.37
Compound: 8539_0002 (a.k.a 8539_0002) I Library: Z8539 Analogs
Spearman est: 0.74, adj p: 3.4e−05
Compound: 8539_0011 (a.k.a 8539_0011) I Library: Z8539 Analogs
Spearman est: 0.18, adj p: 0.41
Compound: 8539_0027 (a.k.a 8539_0027) I Library: Z8539 Analogs
Spearman est: 0.7, adj p: 0.00013
Compound: 8539_0026 (a.k.a 8539_0026) I Library: Z8539 Analogs
Spearman est: 0.37, adj p: 0.076
Compound: 8539_0037 (a.k.a 8539_0037) I Library: Z8539 Analogs
Spearman est: 0.16, adj p: 0.47
Compound: 8539_0039 (a.k.a 8539_0039) I Library: Z8539 Analogs
Spearman est: 0.09, adj p: 0.69

Compound: 8539_0041 (a.k.a 8539_0041) I Library: Z8539 Analogs

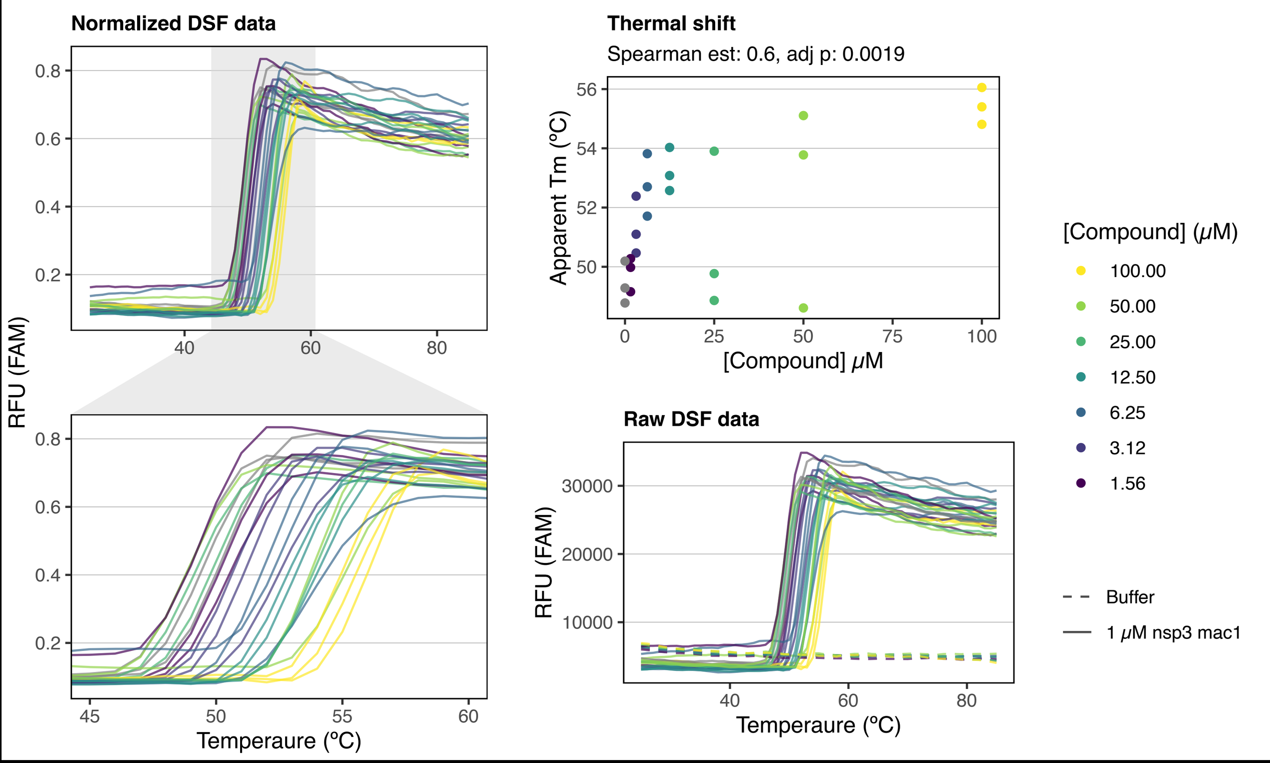

Compound: 8539_0009 (a.k.a 8539_0009) I Library: Z8539 Analogs

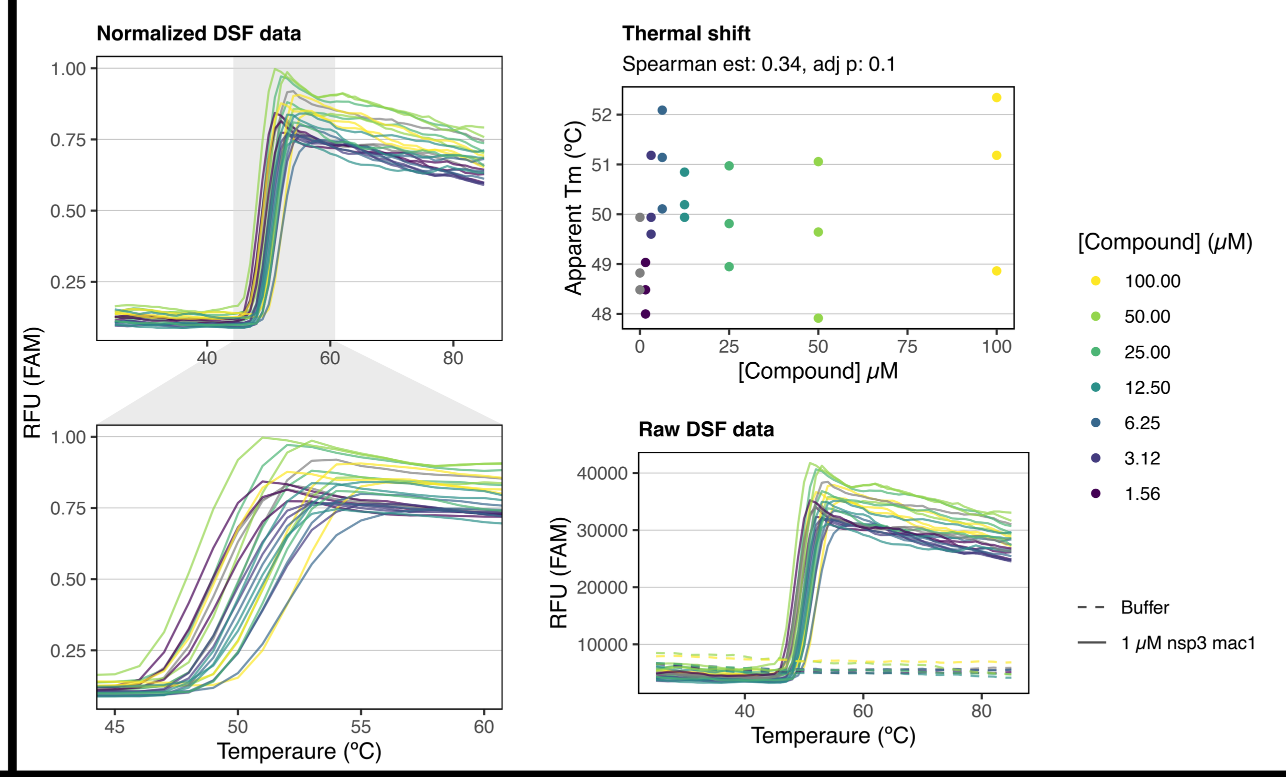

Compound: 8539_0007 (a.k.a 8539_0007) I Library: Z8539 Analogs

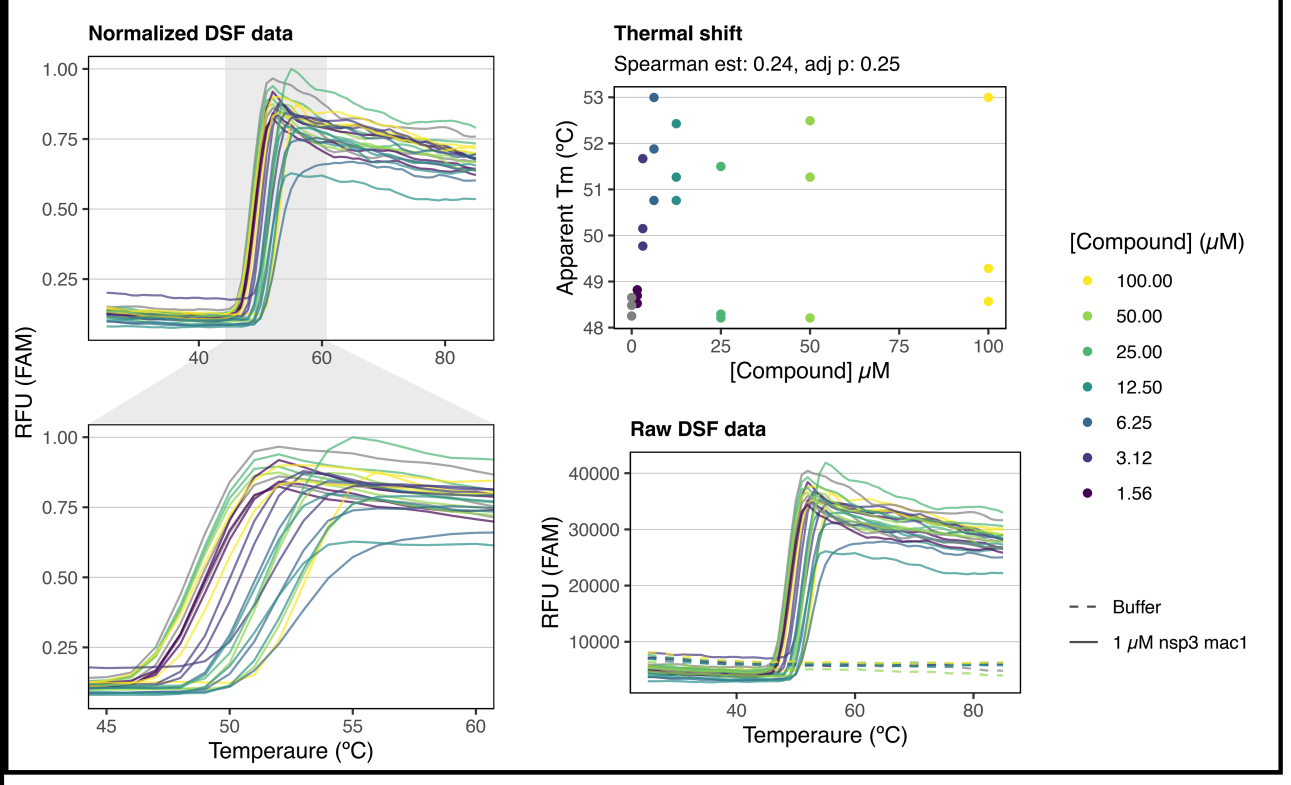

Compound: 8539_0023 (a.k.a 8539_0023) I Library: Z8539 Analogs

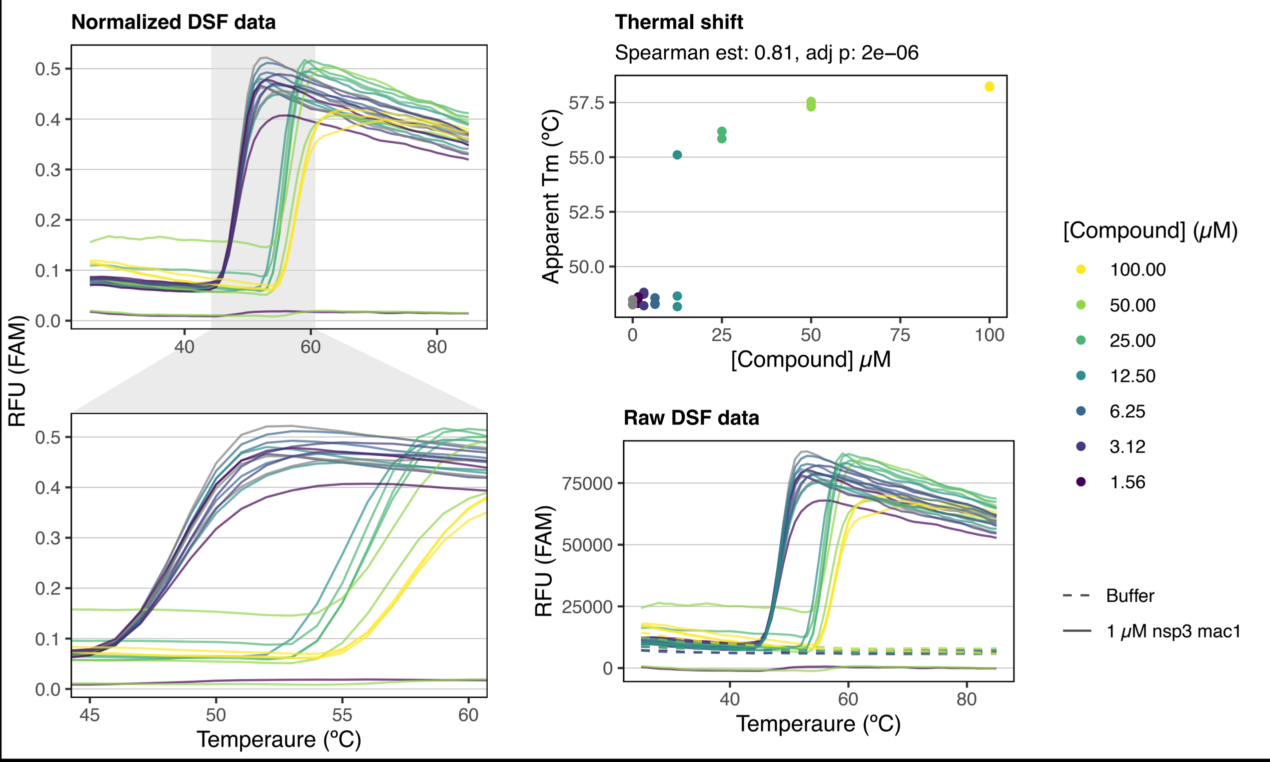

Compound: 8539_0006 (a.k.a 8539_0006) I Library: Z8539 Analogs

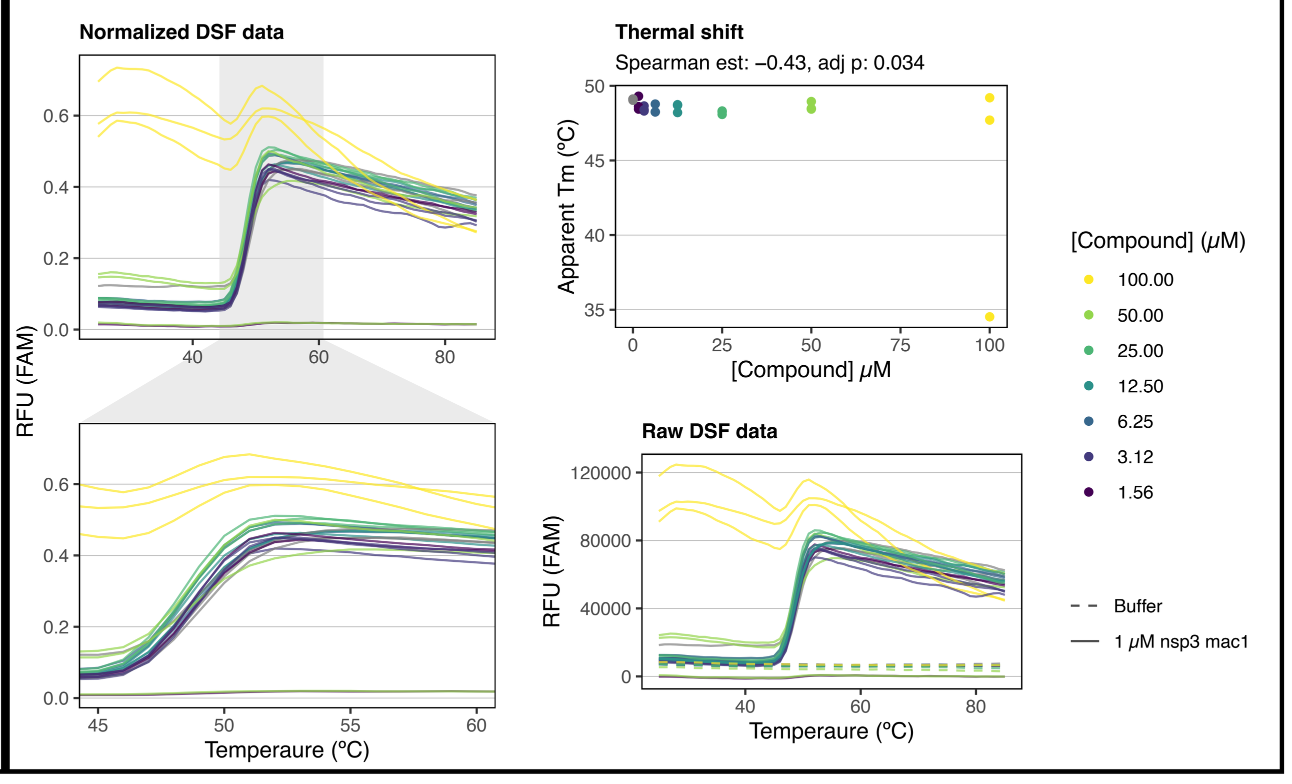

Supplement: Supplementary file 8 — Dataset S07 (PDF) [file pnas.2212931120.sd07.pdf]
